# Supplementary material for: Genome-Wide Identification, Characterization and Expression Patterns of the Pectin Methylesterase Inhibitor Genes in Sorghum bicolor
Source: Genes (Basel). 2019 Sep 26;10(10):755. doi: 10.3390/genes10100755 (PMC6826626; doi:10.3390/genes10100755)
Supplement: Supplementary file 1 [file genes-10-00755-s001.zip › Supplementary Files/Supplementary File 1.docx]

| **Gene name** | **Forward primer** | **Reverse primer** |
| --- | --- | --- |
| *SbPMEI2* | TGTTCCTGTCAGTGTCCCTC | CTTGATGCTGTGGTCGTCC |
| *SbPMEI4* | TGCATGGAAGGGTTCAAGGG | ATCGACTCCCATGGACTTGC |
| *SbPMEI19* | GGTACAATCGAGGCGCTCAAC | GACGACCTCCATGTCGAACAC |
| *SbPMEI20* | TGCGGATTACAGGGGTGAAG | CTCTCCTTGCACGTCTCCAG |
| *SbPMEI28* | AGCTAGTCTCCTCCGTGTTG | CAGTAGTCGTAGCCGATGGA |
| *SbPMEI34* | TTGGTCCCTCAGCAACGTC | ACCCTCCTCTTCACCCTGAC |
| *SbPMEI35* | GCAGCTGATGAGCAACAGAAC | GTCGTAGAAGCTGGTCGAGT |
| *SbPMEI54* | CGAGAACACCTGCCTCGAC | CCCGACCTTGTTGACGAGC |
| *EIF4A1* | CAACTTTGTCACCCGCGATGA | TCCAGAAACCTTAGCAGCCCA |

**Supplementary File 1.** Gene specific primers for 8 *PMEI* genes and actin in sorghum for qRT-PCR
