# Supplementary material for: Genome-Wide Identification, Characterization and Expression Patterns of the Pectin Methylesterase Inhibitor Genes in Sorghum bicolor
Source: Genes (Basel). 2019 Sep 26;10(10):755. doi: 10.3390/genes10100755 (PMC6826626; doi:10.3390/genes10100755)
Supplement: Supplementary file 1 [file genes-10-00755-s001.zip › Supplementary Files/Supplementary File 2.docx]

**Supplemental File 2.** Detail information on sequence similarity of putative paralogous pairs in a 100 kb region within an individual chromosome.

| **Paralogous Pairs** | **Score** | **Identities** | **Similarity** | **Gaps** | **Distance (in Chromosome, kb)** | **Tandem duplicates** |
| --- | --- | --- | --- | --- | --- | --- |
| *SbPMEI1- SbPMEI2* | 92.0 | 44/204 (21.6%) | 80/204 (39.2%) | 26/204 (12.7%) | 4.672 | N |
| *SbPMEI1- SbPMEI3* | 147.0 | 57/248 (23.0%) | 87/248 (35.1%) | 69/248 (27.8%) | 21.31 | N |
| *SbPMEI2- SbPMEI3* | 122.0 | 53/243 (21.8%) | 91/243 (37.4% | 51/243 (21.0%) | 15.37 | N |
| *SbPMEI4-SbPMEI5* | 687.0 | 153/251 (61.0%) | 171/251 (68.1%) | 40/251 (15.9%) | 13.516 | N |
| *SbPMEI9-SbPMEI10* | 668.0 | 151/335 (45.1%) | 177/335 (52.8%) | 100/335 (29.9%) | 3.437 | N |
| *SbPMEI11-SbPMEI12* | 712 | 146/199 (73.4%) | 151/199 (75.9%) | 16/199 (8.0%) | 14.406 | Y |
| *SbPMEI13-SbPMEI14* | 64.0 | 46/226 (20.4%) | 69/226 (30.5%) | 82/226 (36.3%) | 2.266 | N |
| *SbPMEI13-SbPMEI15* | 284.5 | 80/218 (36.7%) | 116/218 (53.2%) | 24/218 (11.0%) | 25.966 | N |
| *SbPMEI14-SbPMEI15* | 90.0 | 43/207 (20.8%) | 64/207 (30.9%) | 56/207 (27.1%) | 22.947 | N |
| *SbPMEI16-SbPMEI17* | 492.5 | 99/183 (54.1%) | 122/183 (66.7%) | 15/183 (8.2%) | 29.379 | N |
| *SbPMEI24-SbPMEI25* | 472.5 | 111/204 (54.4%) | 127/204 (62.3%) | 20/204 (9.8%) | 20.018 | N |
| *SbPMEI24-SbPMEI26* | 451.5 | 106/190 (55.8%) | 122/190 (64.2%) | 11/190 (5.8%) | 35.007 | N |
| *SbPMEI24-SbPMEI27* | 385.0 | 88/189 (46.6%) | 111/189 (58.7%) | 19/189 (10.1%) | 51.743 | N |
| *SbPMEI25-SbPMEI27* | 423.5 | 29/199 (14.6%) | 118/199 (59.3%) | 94/199 (47.2%) | 30.790 | N |
| *SbPMEI25-SbPMEI26* | 403.5 | 96/199 (48.2%) | 115/199 (57.8%) | 19/199 (9.5%) | 14.054 | N |
| *SbPMEI26-SbPMEI27* | 381.0 | 87/183 (47.5%) | 109/183 (59.6%) | 16/183 (8.7%) | 15.42 | N |
| *SbPMEI28-SbPMEI29* | 148.0 | 50/193 (25.9%) | 82/193 (42.5%) | 28/193 (14.5%) | 35.366 | N |
| *SbPMEI29-SbPMEI30* | 379.0 | 85/183 (46.4%) | 110/183 (60.1%) | 5/183 (2.7%) | 2.917 | N |
| *SbPMEI37-SbPMEI38* | 477.0 | 105/188 (55.9%) | 118/188 (62.8%) | 11/188 (5.9%) | 5.26 | N |
| *SbPMEI39-SbPMEI40* | 693.5 | 148/243 (60.9%) | 176/243 (72.4%) | 20/243 (8.2%) | 8.756 | Y |
| *SbPMEI39-SbPMEI41* | 738.5 | 157/244 (64.3%) | 175/244 (71.7%) | 29/244 (11.9%) | 13.945 | Y |
| *SbPMEI40-SbPMEI41* | 652.5 | 139/237 (58.6%) | 168/237 (70.9%) | 29/237 (12.2%) | 3.825 | Y |
| *SbPMEI42-SbPMEI43* | 599.5 | 127/184 (69.0%) | 143/184 (77.7%) | 7/184 (3.8%) | 9.681 | N |
| *SbPMEI45-SbPMEI46* | 91.0 | 49/201 (24.4%) | 77/201 (38.3%) | 38/201 (18.9%) | 2.556 | N |
| **Paralogous Pairs** | **Score** | **Identities** | **Similarity** | **Gaps** | **Distance (in Chromosome, kb)** | **Tandem duplicates** |
| *SbPMEI45-SbPMEI49* | 396.5 | 94/199 (47.2%) | 117/199 (58.8%) | 22/199 (11.1%) | 8.580 | N |
| *SbPMEI45-SbPMEI68* | 123.0 | 59/242 (24.4%) | 81/242 (33.5%) | 68/242 (28.1%) | 9.943 | N |
| *SbPMEI66-SbPMEI68* | 482.5 | 56/238 (23.5%) | 123/238 (51.7%) | 109/238 (45.8%) | 6.833 | N |
| *SbPMEI46-SbPMEI49* | 121.5 | 57/218 (26.1%) | 84/218 (38.5%) | 61/218 (28.0%) | 8.648 | N |
| *SbPMEI46-SbPMEI47* | 108.5 | 48/218 (22.0%) | 76/218 (34.9%) | 56/218 (25.7%) | 5.47 | N |
| *SbPMEI47-SbPMEI48* | 125.5 | 64/249 (25.7%) | 91/249 (36.5%) | 66/249 (26.5%) | 0.497 | N |
| *SbPMEI47-SbPMEI49* | 544.5 | 119/199 (59.8%) | 142/199 (71.4%) | 11/199 (5.5%) | 2.312 | Y |
| *SbPMEI48-SbPMEI49* | 116.5 | 58/259 (22.4%) | 78/259 (30.1%) | 91/259 (35.1%) | 0.423 | N |
| *SbPMEI52-SbPMEI53* | 77.0 | 55/244 (22.5%) | 77/244 (31.6%) | 65/244 (26.6%) | 40.132 | N |
| *SbPMEI54-SbPMEI55* | 886.0 | 179/217 (82.5%) | 187/217 (86.2%) | 15/217 (6.9%) | 4.265 | Y |

Y: The paralogous pairs were tandem genes duplicates; N: The paralogous pairs were not tandem genes duplicates.
