# Supplementary material for: Genome-Wide Identification, Characterization and Expression Patterns of the Pectin Methylesterase Inhibitor Genes in Sorghum bicolor
Source: Genes (Basel). 2019 Sep 26;10(10):755. doi: 10.3390/genes10100755 (PMC6826626; doi:10.3390/genes10100755)
Supplement: Supplementary file 1 [file genes-10-00755-s001.zip › Supplementary Files/Supplementary File 3.docx]

>SbPMEI1

MAASLRVLIVVLAVVSVSVRRAAAATITVDEACKQYTKYPELCVKSLSSAKPEAKAAAEQGGLTGLAELSLAQAAQVGTETVAFVKGLENTPGGMPPVCLNECLAKFQGALADLQRSKVAVQEAKDVGAVNTWLSAAKIDGDTCMNDCQKVEGGGEMQVVDKIGDLGRMCSIAMSLTDASRNHTAAA

>SbPMEI2

MEARSAISWYCGSLLAVVIALFLSVSLGVRAAGVDLKASCAATPHPDVCLRALQDDHSIKGASTQRDLASAAIRAAATAGGAVGDYARDELNVVKDNLMWQCLNECAEDIEEALDHLDDSEGGLDDDKLRDVKEFLDTAEEDTWSCDESCKHAPNTPIKTTLLAKNKDFAAVMRVANALIKRATAGDSPAPRFIN

>SbPMEI3

MASVRTTTTTTSSLVAAVLSLCVVVSLSLRGADAARITPGDSPIVATCMTGPYPELCVGELGKRLLDVQTVIASAAPNKGAAKIAGAPGQVDVKALVSVALEAATEAGTILVSIFEGKLPGFNTSVPDFHKCMGNCSVTMKSAMQKLHGAKAALHAGDRQVAKTLALRAATDVSSCTISCRELNGDVRVIVAQSLTEFAKMLQIAIGFISKMKSEPSEPKPPSEPKPPSEPNPPPTRTTP

>SbPMEI4

MALARTASSSLLLLLVLSCWCGATTARPAPTSDAAGTGAGAGAGFVKSWCAGTEYPALCDATLASYAAEVGTSAARLSWAALTVTLDGARDATAAMKGMAAAGHLAPVAAEAARDCVSMLGDAVDMLRQSVETMARLGEEEEEKQQGQPGSSSRRNVRFQVDSVQTWASAALTDDDMCMEGFKGEAAVVREAVRGHVVGVAHLTADALAIVNAMGKSMGVDICRGSCKSTPTPPPATAP

>SbPMEI5

MARSRALLLFLLALSCCWSWWCGAVVVTARPTPSTTTTAGGGGGGGFIASWCAGTDYPALCNATLAPYAAEVGASPARLSLAALTVTLGGARKATAAMKAMAAGASRSSPVAAEAAEDCVGMLEDAVGLLRQSVEAMERIGKEEEEPSGSSGQQGGSGSSRSVRFQVNSVQTWASAAMTNDDMCVEGGQAAVVREAVRGNVAGAMHLTANALAIVNAMAKQIS

>SbPMEI6

MASAAAAFLLIIAGAAWPSLIDAAPSPSSSSFVPPPPCAPPRSAVEFLRARCASTLYGVACYESLLPYACIFRTSHVKLARAAGDVNAAWISSISKRVKELVARGAAGGTAVAESAALRDCASTVSSAAGLAKQAAAELAKLDAAGGAVGSSNVRWAISNAQTWLSASMTNEATCADGVAATGAAASSPVAREVVMAVVRARELTSIALALVYGIPVPP

>SbPMEI7

MPIVLAFLLVVAAACQPAASASSSAPASSLALPAPAPAPVPDASGHGSRRAAQAEAEAAEFVRASCGRTLYPRLCYAGLAPYAASVRSSHARLALASANLTLAALDALAARIPSPSPGSGSGSGALSDCADAVASAEDQAARAAERLGGVEQAVGGRPELLWRVDDALTWLSAAMTYEDSCADSLGPRKSAPAPVRAELRARVRRAKQFTSISLALVNILVSNPRS

>SbPMEI8

MNIGVMAAATTTTISSTLAVVLICAASLVATGAGAGAGGHHLEPNDLVAKTCATVTRRHYRGPGLTRQFCESALRSDKRSAAARDARDLALVAMDLVQSGAAEAGAKVGGALRSGGAAARWSKYTTLRLQYCRQDHDDVASTAPNCRALVREYNPRAGGGRHGSGNLTPFEYLECAGRLVHAADDCWVHMLDQDGAAKKAVWKEIVEVASRANLAKAMVEQMWENCLGRLGHNQATA

>SbPMEI9

MAMARSVAHLFFLLLLVSTAPAVRTIPDAAAAAAGGNNNNIQEACSRTLFPKVCVQALKDNPECQGGGPAVTPRRLAELLVYVSAEVGMTVAAFAHHELNGIKDDVLYKCLDTCSEDIEEAVAHLSALSRDFSDAKFLEVKSWLSSTLGGTSTCEDACKDAPVSDIKNACVTKSFEFEKLLRVTLDLITEASGSMSAAEVALPPSGGGASAPSSYDAAAPSSGGYGPSAGGAPASGSPAPAPGKSTASDDADATA

>SbPMEI10

MARSVSVVVVLLLSVLVSAASAARTVGDTVQDACSKTQFPKICVDSLAAKPESQKATPRKLAELFVNIAAEKGSGMATFVHGKYNNDAKDSALFKCYDSCSDDVEEAVAHLNGLVREPTDAKFLELKSWLSSTLGGTSTCEDACKDAPKSGDKDAVVNFSLDFEKLQRVTLDLITEASGSMSAGIALPPSDAGAPSSYDAAAPSSSGGGSADAPAGADAGAGAGSEGPAAASGPSSGGDAPADGGSAASGPAAADAPAAAGAASSSDGPSGAPAPSSSDSASGAPGPSSDGGSSSAPAPAGGDDDDDADSDDGSA

>SbPMEI11

MATIPLLVLLMATIFSAAPPVAGEPPNVVPFACKAATAAGGGTFDEAFCLSTLEGSKSSVGAADYADLAVVAVDLATANVTATEAKIDALLASNNISGAAVVQGLQSCRALYGAVVRQYQPECRAAVKGGRYGDGKKCLGRTAQAVAACERWFQQRKVASPVAAEDVVLAMLANLAIALASIAN

>SbPMEI12

MDSTFTDRSTTAIPLLVLLMTTIFLDAPPVAGEPPSEVPFACKGAAAASGGTFTEAFCLSTLHGSKSTVGAADYADLALVAVDLATANATATEAMIDALLAAGTNGATPEGLGLQSCRALYGAVVRQYQPECRAAVKDGRYGDGKACLGRTAQEAAACERWFQQRKVASPVAREDDALAKLASLAIALASIA

>SbPMEI13

MAASTSRTSPGNHKVLLAALLCIAAFFPGSSASTPLVAQTCGRTSNQRLCVSLLESSNRSRSATTVRDLAIIAVRGARRSVLRARLRAWDLSHGARRETTTPAAGRLVARCAALYRDCLHAAAHALARVTHMPAYDDGGRVAADDAVAALRVFPEKCQRLFDAQEIVSPLEQVNRDTEDKLRVASEIVHLLRRSRLEPPSPSRHGDVERTNV

>SbPMEI14

MAATGLYGMVTFLLLLSLPAALADPSFFNRTCYLTKNPGACHSVLGHYFWSLNATTLPQLTSTGLDVAVFKARDIMGAMSELFGEGRYAGTHEGDALAQCARLYDKTMSYDLDQGDMRLVDGKYSDALRFVTHARSAGDACEKAFADRGSRSVVSDLN

>SbPMEI15

MATSRAASLASLKSVSAVAALLCAAAVASSFLPLSSAGVSLLSRTCSKTAHERLCISTLAPDGRSDAAQSVQELAAIALKVARNSTRDAVWRTTVLAGARVRTPLERDRLAQCRALYNECLRETTRTIGLVTAASYDAAARASSTLHWYPEKCQSLLYKQGVESAMEQTNKQVEEQLIASTDLVHLLLVRRHGAGKLNLE

>SbPMEI16

MAGARVLLIVLAAAVALLAARPVAATGGVAGVEEVCRSTPFPDLCTRTAGKHAEKYKVVDAVTVLEMQVDAFKKRVKAARRVAKQEVKTAAPTPLVRRALNLCKSYYLDAGDNLGACKRAIGFRDAVTIRATMSMVAQDMQNCDEEFRKAGSTNPMEDHNRSLIEMSEICRTLSNMVPYEHTH

>SbPMEI17

MAGARTLLLLLVAALCLATVNAATLAEICKGTAFPDICTSTVGPEAASNPVLDPMAVLRMQVDAFNQRTEAARAHVKEAAMTASPKARTVLDLCNNLYLDVEDNLGACRRAIGFKDAVTIRATMGMAAQDMQNCDEQFRQIGEPNPMEQFDASLVEMSENCRSLSNMI

>SbPMEI18

MASEMSYAAVGVVILSVLVVVAAASADPTAAPTAAPSSSKLSLEEACKQTAGHHDLCVATLSADPSSKTADTAGLARLAIQAAQRNASETAAYLSSFYDDDSLENKTAQLQQCLEDCGERYESAVEQLSDATSAVDTGAYSECEALVVASQAEVKLCQRGCQGVPDHRNVLTARNRDVDQLCSIALTITKLVGGPPS

>SbPMEI19

MAARLVSVSVPLLLLVVVLAGSRAALASETVDQTCAAAPRPRRRKEHLASFCVSSLQAALGSEGADARGLAAIATNLTLANYTAAVATIKALERRGGWPERSRRALATSRAAGGTIEALNVVHSAVHALATGKLRGYVFDMEVVRKAASDCEDAFGGAGGNCKLPLRKVDDDADNLTVVAMLIVRSLGN

>SbPMEI20

MFRYLAPLILVAALAASTTNKSVDARVVHPIVGPDTQADLEAAGPSPADDGDDGRRLIGTAGGGHDELVALCQQLHYKTLCTTMTTLPGVTTPEQLLDTALRITGVKAAMAETKLDEAIKSSGGAQAGNPLMSSLETCKESYASLVDSINTSRDTLKSGGSNSDLMTELSAAATYSTDCEDTFEERPELVSPIPGAQRHISRLVSNCLDLAATIKEEP

>SbPMEI21

MSYHTKKKKKKMPPPPCYCSSLITITIILLLQQQNNPWTTAAAASAMATTTTKLGSSPLSDVVKDTCERCRQGNPQVNYTLCVSSLSSDPKSRQADLHELAMISAKLVRSGAVGMEAKMAELSRKERPWSRRRSCLEACMGVYHNSLYDLDASIAAIQERRYADAKTSMSATVDAPITCEDEFKEQGLEPPMKAESKRLFQQAAITLAIISLL

>SbPMEI22

MAMMGATTIRGGGASSSVRPAVLVLGLCLLLLLLLGVAQAVEFELVPDAGAIDPMMMPAMDDVDGGGREPPRECMTPVSVEEACRGASELHAGVDYDHCMASLGADPRSKEAGNKNMHGLAVLATKMAIDHAASTESKIDDLAELDADNQSSSPQARRARFNHCLEQYGGAADLLRDALDNLKAKIYGKAMEQLTAAMGASESCEDAWKGEEEIPVAAHDREYGRMAHIAFGFTHAAAA

>SbPMEI23

MSRALLMVVALAAVHGLITLTGVDATVVATCLAASNSDRRVNYDFCVSELNKHRDSPGADTPGLAKVAANVGVNSAGGAVNDIEALLAAKQQPPPDARTSAALRLCEQLYYDMELAFAGAYDEINALNYTAGKQMAADADSLVRRCTGGFAEAGLVPPEPVARRSAYAVQIAIVCTAITNLIISP

>SbPMEI24

MRPSTARALATAAIVAALALSADVVGGTPETTCAAAAAHDRRVDYGFCVSRLSHHHDSPDADTWGLAKVAADVGVAIAGDAVYDIKALLATSSKPPGEGEGDAQERAVLEQCQRLYDAAESAFAEAYDAINRRDYAAGKGKAAEAASLARRCDDAFARAALRPPPQVARWGEESAKIAVVCTAITDLID

>SbPMEI25

MTMRPLPQTVVHLLPPVAAVLAFALIGCLVGGASATVVTTCRAAADSDARVDYGFCVAELGMHRESPDADVWGLAKVAALTGVNNADNAVYDIKALLLLAADDGAKSRSPPPDGPTRAALEKCGRLYDSVGFAFAEADDEINNRRYAAGKGKVAEAVSLARQCDDALAKAAAVPSPLAQHSSYNVRIANICTAITNLIK

>SbPMEI26

MSPWKTLLVAAAALATLLAADATVESTCKAAAAMDVRIDYGFCVSELSKHRDSPGADTWGLAKVAANLGVNNAGGAVREADALLARPPGTGGADDAKARAALGQCRRLYFDMELAFAGAHDEIDARQYAAGKEMAVEGIPLARRCDAVFAEARIPSLLARRGEYAEQIAVLCIAITDLIK

>SbPMEI27

MSPSSILVTTSAIVAIILVLHGADATVVTTCKAAAESDKRVDYDFCVLELGKHHESPDADIWGLAKVAALVGAANTGNVLVEIRARLAKPGTDAKTTTVLRQCLKLYDAADDAFLNAYERINERNYAAGKEEVRCDDAFTKVASPSPLNQSSTYTTKISIVCIAITNLIK

>SbPMEI28

MKLVSSVLFALLILPMCRSSPLQDTCRSFAAGHPSIGYDYCIRIFQADKASAEATDARGLAAIAARLAEAKANATAARVASMSALEGDARRRDRLSVCAEVYSDAVDQLDQAEEELAHGAEGGIDDAVTQLSAALDAPETCEDAFREADDTSPLAAEDAEFKKLATVALAVAASLTPPPA

>SbPMEI29

MRSFLVQPVSILLLLLFITAIAPVVTAGGSPVINATCAALKSLQPYDYCVGVLSADPAAAAATDVRGVAAAAVNITAQKAASTLLVINYLAGDLNTCRGYYSNMLQSLENSLVHFRDGRFLNASLGIANATGDPTGCDLLLFEGKTHKDPISDENYENMRLVDLADGIVDLFANKRLY

>SbPMEI30

MAYYIKSSAMVPVLLLAVLAIAPVLAIATTSAINATCTALDAQHYDHPYAYCVGVLSGDSAAAAATDERGVAAAAINIAAHKAAATVSVVTYLVDELSLCSKYYGRMVESLTAVLADFHAGRFDDAALAKARSASEVPNDCDVILLQGSAKKNPFSQENIDNGRLSGLARDITALVANKGPSS

>SbPMEI31

MAMAATPTATASILLLALFLAGAHAEPAELPCALPACKTVGGGSQFFDVQFCLAALGSDGRSISHCMDYQVYSVIAADLLAANVTATAAKIDGLLQGSGGGGGGDDAAATARCLRSCQALYGGTVRRQPGCAAAVRGVRKGEATTCLEEAAAAAKQCEDGFQSSKVASPVTAENQNAFMLAKLAVALLREVYANK

>SbPMEI32

MAAPQPRALTTHHHHLLLLLLIVVFTMASAHTTAAPAPRAAAAAAESPSPAAMSFLRARCATTLYPALCYDSLLPYASEVQDNPARLARVAADVAAARLRALSARVKDILRHVGGDPAEGAAALRDCASTVSAAASLARQSSAELTKLEPDAGRVVTTSAGDGMSSSRQARWEVSNAKTWLSAAMANEGTCADGLVEAGAAAAAGKEVTAGVAAVKQYTSNALALVNGIPL

>SbPMEI33

MRVPLLPFVLIVAVVATTVSLVPAVCNGQEAATAGEEHGGSIKPLSLDGYGPLEKAAKKPKEQTLNAQASPAVPADTYDQKPDKYVASSLVPAKEEEETPAEVKKEKKEKSDYLDESTSSKKEKKEKSDDSDVSTSSKKKKKKAKTDDSDEDASLTKKEKKEKKHKKHKSDDDDLDSTSPKKHKKEKSIDSDASSYLQKEEEKSGGDSDEATSLKKHKKEKKNKKKKEEKSGENADEDDAAPVDVSTDGQYVSPSSKEEKSDEDDAMPVDVSTTTGQYVSSPKSKGGERQVSTPTDAYTSPDELPPAAKSSTTSDAYAPPKQQVVSSSSSSSQPIAGGSPDELPPAAKSSATADPYLSSKHQVVSSQPMAGGAPDEVPPNVAANGQPNLPAAGNKPKLSMGTLSGMIKKPIAKFLSPVIKSVCAKTEYPVDCEASIGGLPGAASAAATDSVGVLKLAMEAVRQKVIVAMNAATDRMNAPGVDGTTKDALDSCTSSYSDIKTSLDSVDDALKRGDVDTAHTNLDSVETDLTTCDDGFQEHGIPSVMTDHDQELQKLASNLLSIGAAIHR

>SbPMEI34

MTLPRRRHLVLLAGLLVLVVVAATATASTDSSSTTAVAVDFVRRSCRSTEYPRVCETTLVPCAASVGRSPRRLARAALVVGADRARNCSAYIHGSRSGSGGAMKDCAELARDAEDRLRQSAAEMERMGRAGTPRFAWSLSNVQTWASAALTDTSTCLDSLAQHKDRGRGGKGSDGDDDAVRVKRRVVAVAQATSNALALVNRLQPATHRQRLLL

>SbPMEI35

MAATRASSLLILLLIIQLNLLFHLPAGSSSVTAVAQAADEQQNTKQQHRQPALVQSTCNSTSFYDVCIAALAADPSSSTADVPGLCAIAVSAAAANASGTAAFLGNASSDAAAAAAGTPEAADYRALLRACAGKYAAARDALLEARASLAQQAYDYAFVHVSAAGEYPAVCRTLFRRRQQRGGSSRPYPPELAKREEALRRLCTIALDIISLLQNQEPK

>SbPMEI36

MKENPLSKVSTSQHGYKNPSKACRQIKRQRHTNLALAVAAHAPSQQATMAARLVSVSVSVPLLLLVVVLAGSRAALASETVDQTCAKATSGAQHKEQLASFCVSSLQAAPGSEGADARGLAAIATNLTLANYTAAVATIKELERRGGWPERSRRALATCRQRYIEALNVVHSAVHALATGRFRDYVSDMEVVRKAASDCEDAFGGAGGNGMSPLRKVDDDADNLTVVAMLIVRSLGN

>SbPMEI37

MKLLQATVSLVFLLACSTSNASVLHDACQSFAAIRHKDADYNYCVRFFQADKESATADHRGLAVIGAKLIEATAKSTGSLIATMLTSEKDKEKLGCLVACGKGYLDAMDEIGKAAKGIVSRKDGGVEDAVTALGGALDAPLDCEDGFQKLHKPSPLAAEDARFRKEASITLFVTGTLLPQINSSKLGI

>SbPMEI38

MKLLQALCPLVFLLACSTSNASVLQDACKSFAAKHPETGYYAYCIKFFQADKGSGSADKRGLAAIAVKITGAAAKSTAQHIAALRASEKDTKRLAGLKDCSEVYSQAVDQTGVAAKGIASATPRGRADAVTALSAVEDAPGTCEQGFQDLGVPSPLASEDAEFRKEASIALSVTEAL

>SbPMEI39

MAAAATTKNVMVLLLLALLPLATLSSRAGPSSAYKSHGHSHRSSPSAKHPPPSPSPPSSPPSAPSPAPAATAALVRATCNSTAYYDLCVSALGADPSSATADVRGLSAIAVSVAAANASGGAATAAALAANGTAPTGTAAAASSSTVVDGTVQALLHACAAKYASARDALAAAGDSIALEDYDFASVHVSAAAEYPQVCRTLFRRQRPGQYPAELAAREETLKQLCSVALDIIGLLSNSS

>SbPMEI40

MAASTRKAALVLTLTMALLAPSILGARTRPPSSPHHSQGHKRSPPPASPPPPPAPAAPTAAAGLVQSTCNATAYYDLCMSTLGADASSATADVRGLSSIAVSAAAVNASGGAATAVALLATAGAGGGNTTTAVDGTTQALLRTCATKYGEARDALSAARDSIAQENYDYASVHVSAAAEYPQVCRVLFQRQRPGEYPPELAAREEALRRLCTVALDIITLLTNNTN

>SbPMEI41

MAMVVLLLLSLLPLSTLGSRSGPTPAVPHHGHAGHGTPKHSSPPPQPTTAELVRSTCNSTAYYDLCVSALGADPSSATADVRGLSTIAVSAAAANASGGAATATALANGNGTATSSNAQAAAPAATAATALLRTCAAKYGQARDALAAAGDSIAQQDYDFASVHVSAAAEYPQVCKALFRRQKPAGGQYPTELAAREEALRQLCSVALDIIALASNTSS

>SbPMEI42

MAYVAAAVLAAVSLTALLFAGGEACANVPSMTSTEACQQTNKWEQLCQQTLQTAPDTAEVTVFALVATRLAKSAYEDTLSALDQMLGPGNLPGAERLAIDNCKETYSTALSKMAGVVDHMSACDFSLASKEYIDAEAGVRSCLEGLQPYQFLPLFGKVSADHDLTLVAYLLGAIIVGR

>SbPMEI43

MAYIAAAVLAVLVSLTALFAGGEACNNVASMTWTAACQQTDRWEKLCQQTLQDTAPDTAEVTVFALIATRLAKLRYENTLSEVDTLLRPGNAPAESRAALDNCKVKYGSARRLLAGVSDQMFACDFSLARQEYIDAEVGVRSCQDGLLRLPNQYQSWPLFRKVSDDHELTVVAYLLGAIFLGR

>SbPMEI44

MTPPSPPPPLFLACILLTLLLAAAVAPPAVAVCVPKGHSKPGAPAKAKAKAKPKPKPAPPKPTAIPIAPGADIVRSLCVKTDYPDLCTSAITKQPQPQLPAGKRLDGAGVLRLAMSAVRAKAAEAKAAAGALAKDPKTQPLARNPLQDCVESFDDIAYSLDQAQKALAGGDRDTTGTMLDTVRTDVDTCDQGFEERKQLTPVMSKHDAELAKLSSNCLAIATAAGLR

>SbPMEI45

MAVVCGGSSAAVGAANSALDQVCESVGGSYVTPELCASALCYDAASPCRDARDYAAVATLAAGLLVRNGTATRDTVAVAAAAANATAGLKSCLQLYDGLLPALEWAAGSVAAGRAYGAARELMQATQFAQRACAGMVAGAEMPRENGGFVTMARVAHAVLSTSVPKTD

>SbPMEI46

MRTLLVAAAAAALILSAAAGALGVAPADTVADSCHAISDFVDMDFCTSRLRSVPGAAAADRFGHLLMATDLAVASGAKAQDLAAAAARDDGSVVVDPGERDAMQACAFLYGAASVPGLRLLRQYAAARNWAPAHALVMLTMDAGDGCDAAVGGSNGRMAGPNHEFDQLSAMVTALLNSINLYDE

>SbPMEI47

MAAGSSSCSPVLLLALLLAVSAGAGAGAGEASAPALDQVCGRLGSYYVTPSLCISALCADASTSTSTCRAARDAPAVAALAARLAADNATAARDSIQAAVFSPSSSSSSSATAAAAAARSCLQLYAGAVPALRWAARAVAAGRYRGAREVLQATQYVAAGCEGIAGDAAAALPRENDGFADMAFVAHAVVASMSAD

>SbPMEI48

MMHSLLVQPPNGHRYCDENATPVMHAGVALAAAGRTANVHQQARAMGRTTATTLLAVAGAALCFFSCCFYGGAAAGDTVAESCDAIRDFVDVSFCASRLGSVPGAASADRHGHLLMAADLAAASGASARDAAAGMARRRRDGEGEGEGDTDALEACGILYGAASVPALRLMRGYAAARAWGAARALLPLTGQAGIGCDAALEGSATAKARMAAANREFDQLSTMATAFLNKLTLVT

>SbPMEI49

MAASASTPTLSATLVVFVSVVIAVGATTALDQVCGGLGGYYVTPELCVSALCPDPSPSSPCRAARDAPAVAAVAARLAAANATAARDSVQAALSFYAAAAGDDDAAAGKKAALRSCLQLYGGVVQALQWAAGSVAAGRFPGAREVMQAAQYVPAGCDGMVGGGVALPSENEGFATMAFVAHAVLATLSNGY

>SbPMEI50

MGLAYYYHQRLVLLAVVAFLCAGLFPQALGKGHGGAVNPAVAGICSRTPFPEVCKSTAGRHASKYPVIDNLAVLNMQVEAFSKRTAQARQHVAKSARTIPPAQKQALTFCDTMYMNTQDTIGAAQRAITFKDTSTAKIMLQLAVQDFDSCDRPFTQAGIPNPMGKFDKELNQMANNCMTLANMI

>SbPMEI51

MESSRIIVASLLLLLLAFAATAEARVVRELIGENACQQTCNQVHFKKMCQSLTKLPKVTTPRELLLASMRVAAEKAKEAKSRVDEYAARSHEGRPMESILSSCSSGYDNVVQTLEETEKIVATQGTQVDLNTKLSDAVTSAGDCDNAFQDFPEMKDPFLAMQRNVWRLVDNVLNIAVVVKQSGDAHAH

>SbPMEI52

MGGVTVTTPLCYLAVAAASALLLLSTAVAPAQVVVTIEEACRMATSGGAGKVSYDHCVASLASDARSRDAADLHHLAALAARIAVEHAAATEAKIEDLGEVEESPHARARLHHCLDLYNAAADVLRDALDNLHARVYGKASQQLAAALGAAESCEDVWKGEEHVPVAAHDREYGRMALVALGLTSGIA

>SbPMEI53

MAARSMAMAFLTTALAVVLLLGACAASAPCLTTDAAAAAVCQNQQRHDDDDGDLVATACERAKGHEAHHFRGLGLTALTKDFCETTLRSDNRSAAANDTRELALVAMDLASTAAASASTKARSALRSSGGRGGKDRDTEFSLRYCVMDYGTVAAVLPACRVIVEEYSPGDFQAPFDYLECAGRVMDAAGDCWQRVSYEDGELKRALWKDAVDVANRANLAQALVEQMVDFPDDHH

>SbPMEI54

MARLLLLLAAAAAAAFLAVEAASPVASDFIRKSCRATQYPSVCEQSLASYGGTPPPRSPRELARAALSVSADRARAASAYVGRLCGAGTGAKKGSGSRPAAAGPVRDCLENLADSVGHLRDAAQEMGGAGMSRSGTPAFKWHLSNVQTWCSAALTDENTCLDGLSSRGVDAGTRAAIRGKVVEVAQVTSNALALVNKVGPGY

>SbPMEI55

MARPGSSAAAPLLLLLAAAAASILAAAAASPAPSDFVRKSCRATQYPSVCEQSLASYGGSPAPRSPRELARAALSVSADRARAASAYVGRLCGGSSSSAGHKKGAAARKGGAPGSAAGPVRDCLENLADSVGHLRDAAQEMGGAGMSRSGTPAFKWHLSNVQTWCSAALTDENTCLDGLSSRGVDAGTRAAIRGKVVDVAQVTSNALALVNKVGPGY
