# Supplementary material for: Genome-Wide Identification, Characterization and Expression Patterns of the Pectin Methylesterase Inhibitor Genes in Sorghum bicolor
Source: Genes (Basel). 2019 Sep 26;10(10):755. doi: 10.3390/genes10100755 (PMC6826626; doi:10.3390/genes10100755)
Supplement: Supplementary file 1 [file genes-10-00755-s001.zip › Supplementary Files/Supplementary File 4.docx]

>SbPMEI1

ATGGCTGCCAGCCTTCGCGTCCTCATCGTCGTCCTCGCCGTCGTCTCGGTCTCCGTCCGGCGCGCCGCTGCCGCCACGATCACCGTGGACGAGGCGTGCAAGCAGTACACCAAGTACCCGGAGCTGTGCGTGAAGTCCCTGTCGTCCGCGAAGCCGGAGGCGAAGGCGGCGGCGGAGCAGGGCGGCCTGACGGGGCTGGCGGAGCTGTCGCTGGCGCAGGCGGCGCAGGTGGGCACGGAGACGGTGGCGTTCGTCAAGGGCCTGGAGAACACGCCGGGGGGCATGCCGCCGGTGTGCCTGAACGAGTGTTTGGCCAAGTTCCAGGGGGCGCTGGCCGACCTGCAGCGGTCCAAGGTGGCGGTGCAGGAGGCCAAGGACGTGGGCGCCGTGAACACGTGGCTCTCGGCGGCGAAGATCGACGGCGACACGTGCATGAACGACTGCCAGAAGGTGGAGGGCGGCGGCGAGATGCAGGTCGTCGACAAGATCGGTGACCTCGGAAGGATGTGCTCCATCGCCATGTCCCTCACCGATGCTTCTCGTAATCACACCGCGGCCGCCTGA

>SbPMEI2

ATGGAGGCGAGGTCGGCCATTAGCTGGTACTGCGGCTCCCTCCTGGCCGTGGTCATCGCGCTGTTCCTGTCAGTGTCCCTCGGCGTGCGCGCCGCCGGCGTGGACCTGAAGGCGTCGTGCGCCGCGACGCCGCACCCGGACGTGTGCCTGCGCGCGCTCCAGGACGACCACAGCATCAAGGGGGCCTCGACCCAGCGGGACCTGGCGTCGGCGGCGATCCGCGCGGCGGCGACCGCTGGCGGCGCGGTGGGCGACTACGCGCGGGACGAGCTGAACGTGGTCAAGGACAACCTGATGTGGCAGTGCCTGAACGAGTGCGCCGAGGACATCGAGGAGGCGCTGGACCACCTGGACGACTCCGAGGGCGGCCTCGACGACGACAAGCTCCGCGACGTCAAGGAGTTCCTCGACACGGCGGAGGAGGACACCTGGTCCTGCGACGAGTCCTGCAAGCACGCCCCCAACACGCCCATCAAGACCACCCTGCTAGCCAAGAACAAGGACTTCGCCGCCGTCATGCGCGTCGCCAACGCGCTCATCAAGCGCGCCACCGCCGGTGACTCGCCGGCGCCAAGATTCATCAATTGA

>SbPMEI3

ATGGCGTCGGTGCGCACGACGACGACGACGACGTCGTCCCTGGTAGCAGCAGTCCTGTCCCTCTGCGTCGTCGTCTCCCTCTCCCTCCGCGGCGCCGACGCCGCGCGGATCACCCCCGGCGACAGCCCGATCGTGGCCACCTGCATGACGGGCCCGTACCCGGAGCTGTGCGTGGGCGAGCTGGGCAAGCGGCTGCTGGACGTGCAGACGGTCATCGCGTCCGCGGCGCCCAACAAGGGCGCGGCCAAGATCGCCGGCGCCCCGGGGCAGGTGGACGTCAAGGCGCTGGTGTCCGTGGCGCTGGAGGCGGCCACGGAGGCCGGCACCATCTTGGTGTCCATCTTCGAGGGCAAGCTCCCGGGGTTCAACACCAGCGTGCCGGACTTCCACAAATGCATGGGCAACTGCAGCGTCACCATGAAAAGCGCCATGCAGAAGCTCCACGGCGCCAAGGCCGCGCTGCACGCGGGCGACAGGCAGGTGGCCAAGACGCTCGCGCTGCGCGCCGCCACCGACGTCTCGTCCTGCACCATCAGCTGCAGGGAGCTCAACGGCGACGTGCGCGTCATCGTCGCGCAGAGCCTCACCGAGTTCGCCAAGATGCTCCAGATCGCCATCGGGTTCATCAGCAAGATGAAGTCGGAGCCGTCGGAGCCAAAGCCACCGTCGGAGCCCAAGCCACCGTCGGAGCCAAACCCACCGCCGACGCGGACCACGCCATGA

>SbPMEI4

ATGGCACTGGCACGTACCGCCTCGTCCTCGTTGCTGCTTCTCTTGGTGCTCTCGTGCTGGTGCGGCGCCACGACGGCGCGCCCGGCGCCGACCTCTGACGCCGCGGGCACGGGCGCGGGCGCGGGCGCGGGCTTCGTCAAGTCGTGGTGCGCGGGGACGGAGTACCCAGCGCTATGCGACGCGACGCTGGCCTCGTACGCGGCGGAGGTGGGCACCAGCGCGGCGCGCCTGTCGTGGGCCGCGCTGACGGTGACGCTCGACGGCGCGCGTGACGCGACGGCCGCGATGAAGGGGATGGCGGCGGCGGGCCACCTGGCGCCCGTGGCGGCCGAGGCGGCGCGGGACTGCGTGAGCATGCTCGGGGACGCCGTGGACATGCTGCGACAGTCGGTGGAAACCATGGCGCGCCTCGGCGAGGAGGAGGAGGAGAAGCAGCAAGGGCAGCCGGGGAGCAGCAGCAGGAGGAACGTGAGGTTCCAGGTGGACAGCGTGCAGACGTGGGCGAGCGCGGCGCTGACGGACGACGACATGTGCATGGAAGGGTTCAAGGGCGAGGCCGCGGTGGTGAGGGAGGCCGTGCGGGGGCACGTCGTCGGCGTCGCGCACCTCACGGCCGACGCGCTTGCCATTGTCAACGCCATGGGCAAGTCCATGGGAGTCGATATATGCCGCGGTTCTTGCAAATCCACACCGACGCCGCCGCCTGCTACTGCCCCATAG

>SbPMEI5

ATGGCACGCAGCAGGGCCTTGCTGTTGTTTCTTCTCGCGCTCTCGTGCTGCTGGTCGTGGTGGTGCGGCGCCGTCGTCGTCACGGCGCGGCCGACCCCGAGCACCACCACCACAGCCGGCGGCGGCGGCGGCGGCGGCTTCATCGCGTCGTGGTGCGCGGGGACGGACTACCCGGCCCTGTGCAACGCGACGCTGGCCCCGTACGCGGCGGAGGTCGGGGCCAGCCCGGCGCGCCTGTCGCTGGCCGCGCTGACGGTCACGCTGGGCGGCGCGCGGAAGGCGACGGCCGCGATGAAGGCGATGGCGGCGGGCGCGAGCAGGAGCAGCCCCGTGGCGGCCGAGGCGGCGGAGGACTGCGTGGGCATGCTCGAGGACGCCGTGGGCCTGCTGCGGCAGTCGGTGGAGGCCATGGAGCGCATCGGGAAGGAGGAGGAGGAGCCGAGCGGCAGCAGCGGGCAGCAGGGCGGGAGCGGCAGCAGCAGGAGCGTCAGGTTCCAGGTGAACAGCGTGCAGACGTGGGCGAGCGCCGCGATGACGAACGACGACATGTGCGTGGAGGGTGGCCAGGCCGCCGTCGTCAGGGAGGCCGTGCGGGGGAACGTCGCCGGCGCCATGCACCTCACGGCCAACGCGCTCGCCATCGTCAACGCCATGGCCAAGCAAATATCGTAG

>SbPMEI6

ATGGCGTCAGCAGCAGCCGCCTTCCTCCTGATCATCGCTGGAGCCGCATGGCCTTCGTTAATCGACGCCGCTCCGTCTCCGTCGTCGTCGTCGTTCGTCCCGCCGCCGCCATGCGCACCACCGCGATCGGCGGTGGAGTTTCTCCGCGCGCGCTGTGCCAGCACGCTGTACGGCGTGGCCTGCTACGAGTCCCTCCTCCCGTACGCGTGCATATTCCGTACCAGCCACGTCAAGCTCGCCCGCGCCGCCGGCGACGTCAACGCCGCCTGGATCAGCTCGATCTCCAAGCGCGTCAAGGAGCTCGTCGCCCGCGGCGCTGCTGGTGGCACCGCCGTGGCCGAGTCGGCTGCGCTCCGCGACTGCGCCAGCACAGTGTCATCGGCCGCCGGCTTGGCCAAGCAGGCGGCGGCCGAGCTGGCCAAGCTCGACGCCGCCGGAGGCGCCGTCGGGAGCAGCAATGTCCGGTGGGCGATTTCCAACGCCCAAACGTGGCTCAGCGCGTCCATGACGAACGAGGCGACATGCGCCGACGGGGTCGCGGCTACAGGCGCTGCAGCGTCGTCGCCGGTGGCCAGGGAGGTAGTGATGGCAGTTGTGAGAGCCAGGGAGCTCACGAGCATCGCTCTCGCACTCGTTTATGGGATACCAGTTCCTCCGTGA

>SbPMEI7

ATGCCCATCGTTCTCGCGTTCCTCCTCGTCGTGGCTGCAGCATGCCAGCCGGCAGCCTCCGCATCGTCGTCGGCGCCGGCGAGTAGTCTTGCTCTACCAGCACCAGCGCCAGCGCCGGTGCCGGACGCCAGCGGCCACGGAAGCCGCAGAGCGGCGCAGGCGGAGGCGGAGGCGGCGGAGTTCGTGCGCGCCAGCTGCGGGCGCACGCTGTACCCGCGGCTCTGCTACGCGGGGCTGGCCCCGTACGCGGCCTCCGTCCGCTCCAGCCACGCGCGCCTCGCGCTCGCCTCCGCCAACCTCACGCTGGCCGCCCTCGACGCGCTGGCCGCGCGCATACCGTCTCCCTCTCCCGGCTCCGGCTCCGGCTCCGGCGCGCTCAGCGACTGCGCGGACGCCGTGGCGTCGGCGGAGGACCAGGCGGCGCGCGCGGCCGAGCGGCTGGGCGGCGTCGAGCAGGCCGTCGGCGGGCGGCCCGAGCTGCTCTGGCGCGTGGACGACGCGCTCACGTGGCTCAGCGCCGCCATGACCTACGAGGACTCGTGCGCCGACAGCCTCGGCCCCCGCAAGTCCGCGCCGGCTCCGGTGAGGGCCGAGCTCCGCGCAAGGGTGCGCCGCGCCAAGCAGTTTACCAGCATCTCTCTCGCGCTCGTCAACATCCTTGTCAGTAACCCGAGAAGTTAG

>SbPMEI8

ATGAACATTGGCGTTATGGCGGCAGCAACAACGACGACGATTTCCTCCACGCTCGCCGTGGTGCTCATCTGTGCCGCGTCCTTGGTGGCAACCGGCGCCGGCGCCGGCGCCGGTGGCCACCATCTCGAGCCCAACGACCTGGTGGCCAAAACGTGCGCCACTGTGACTAGACGCCACTACAGAGGGCCCGGGCTCACCAGACAGTTCTGCGAGTCGGCGCTGCGGTCAGACAAGCGCAGCGCGGCGGCGAGGGACGCACGCGACCTGGCGCTCGTGGCCATGGACCTCGTCCAGAGCGGCGCCGCGGAGGCTGGCGCCAAGGTTGGCGGCGCGCTGCGCTCCGGCGGCGCCGCGGCGAGGTGGAGCAAGTACACGACGCTCCGCCTCCAGTACTGCCGGCAGGACCACGACGACGTGGCGAGCACCGCCCCGAACTGCCGCGCTTTGGTCCGGGAGTACAATCCGCGTGCCGGTGGTGGCCGCCATGGCAGCGGCAACCTGACTCCCTTTGAGTACCTGGAGTGCGCAGGCAGGCTGGTGCACGCGGCAGATGACTGCTGGGTTCACATGCTGGACCAAGATGGTGCGGCGAAGAAGGCGGTGTGGAAGGAGATCGTCGAGGTCGCCAGTCGGGCTAACCTCGCCAAAGCTATGGTCGAGCAAATGTGGGAGAACTGCCTGGGCCGGCTCGGCCATAACCAAGCCACGGCCTAG

>SbPMEI9

ATGGCCATGGCTCGCTCCGTGGCGCACCTCTTCTTTCTCCTCCTCCTCGTCTCCACCGCGCCCGCCGTGCGTACCATCCCCGACGCCGCCGCCGCCGCCGCCGGCGGAAACAACAATAACATCCAGGAGGCGTGCAGCAGGACGCTGTTCCCCAAGGTGTGCGTGCAGGCGCTCAAGGACAACCCGGAGTGCCAGGGCGGCGGCCCCGCCGTCACGCCGCGCCGCCTCGCGGAGCTGCTGGTGTACGTCTCCGCCGAGGTGGGCATGACGGTGGCGGCGTTCGCGCACCACGAGCTCAACGGCATCAAGGACGACGTGCTGTACAAGTGCCTCGACACCTGCTCCGAGGACATCGAGGAGGCCGTGGCGCACCTCAGCGCGCTCTCCCGCGACTTCTCCGACGCCAAGTTCCTCGAGGTCAAGTCCTGGCTGTCCTCCACGCTCGGGGGCACCTCCACCTGCGAGGACGCCTGCAAGGACGCCCCCGTCAGCGACATCAAGAACGCCTGCGTCACCAAGAGCTTCGAGTTCGAGAAGCTCCTGCGCGTCACGCTCGACCTCATCACCGAGGCTTCCGGCTCCATGTCCGCCGCTGAGGTCGCCCTGCCGCCCTCCGGCGGCGGCGCCAGCGCGCCGTCGTCGTACGACGCGGCGGCGCCGTCGTCCGGAGGTTACGGCCCGTCCGCTGGTGGCGCCCCCGCCAGCGGATCACCGGCACCGGCCCCGGGGAAGAGTACTGCTTCCGACGACGCGGACGCGACTGCATGA

>SbPMEI10

ATGGCTCGCTCTGTCTCCGTCGTCGTCGTCCTCCTCCTCTCCGTCCTCGTCTCCGCCGCGTCCGCCGCACGGACCGTGGGCGACACCGTGCAGGACGCGTGCAGCAAGACGCAGTTCCCCAAGATCTGCGTCGACAGCCTCGCGGCGAAGCCGGAGAGCCAGAAGGCGACCCCGCGGAAGCTGGCGGAGCTGTTCGTGAACATCGCCGCCGAGAAAGGGTCCGGGATGGCCACGTTCGTGCACGGCAAGTACAACAACGACGCCAAGGACAGCGCCCTGTTCAAGTGCTACGACAGCTGCTCCGACGACGTGGAGGAGGCGGTGGCGCACCTCAACGGGCTCGTCCGGGAGCCCACCGACGCCAAGTTCCTGGAGCTCAAGTCGTGGCTCTCCTCCACGCTCGGCGGCACCTCCACCTGCGAGGACGCCTGCAAGGACGCGCCCAAGAGCGGCGACAAGGACGCCGTCGTCAACTTCAGCCTCGACTTCGAGAAGCTGCAGCGCGTCACGCTGGACCTCATCACCGAGGCGTCCGGCTCCATGTCCGCCGGCATCGCACTGCCGCCGTCCGACGCCGGGGCGCCCAGCTCCTACGACGCGGCCGCGCCGTCGTCGTCCGGAGGAGGCTCAGCGGACGCCCCCGCCGGCGCTGATGCCGGCGCCGGCGCCGGCTCCGAGGGCCCTGCTGCCGCCAGCGGCCCGTCGTCTGGTGGTGACGCGCCGGCGGACGGCGGCAGTGCCGCCAGCGGGCCGGCCGCTGCTGATGCTCCGGCGGCGGCGGGGGCGGCGTCATCATCCGACGGGCCCTCTGGCGCACCAGCGCCATCGTCGTCTGATTCGGCCTCTGGCGCACCAGGACCGTCGTCTGATGGTGGGTCGTCCAGCGCTCCGGCGCCAGCGGGCGGCGACGACGACGACGATGCCGATTCCGACGACGGGTCAGCTTGA

>SbPMEI11

ATGGCCACCATTCCGCTCCTCGTGCTCCTGATGGCGACCATCTTCTCGGCCGCACCGCCGGTCGCCGGCGAGCCTCCCAACGTGGTGCCCTTCGCGTGCAAGGCGGCCACCGCCGCCGGCGGCGGCACATTCGACGAGGCCTTCTGTCTGTCGACGCTCGAGGGCAGCAAAAGCAGCGTGGGCGCCGCGGACTACGCGGACCTGGCCGTCGTCGCGGTGGACCTCGCCACGGCGAACGTGACGGCCACCGAGGCCAAGATCGACGCCCTGCTTGCCAGCAATAACATCAGTGGCGCGGCAGTCGTCCAGGGCCTGCAGTCGTGCCGGGCGCTGTACGGCGCGGTGGTGCGGCAGTACCAGCCGGAGTGCCGCGCCGCCGTGAAGGGCGGCAGGTACGGCGACGGGAAGAAGTGTCTGGGCAGGACGGCGCAGGCGGTTGCGGCGTGCGAGCGGTGGTTCCAGCAGCGGAAGGTGGCGTCGCCGGTGGCCGCGGAGGACGTCGTCCTCGCCATGCTCGCCAACCTCGCCATCGCGCTGGCCTCCATCGCCAATTGA

>SbPMEI12

ATGGACTCCACTTTCACCGATCGTAGCACCACCGCCATTCCGCTCCTCGTGCTCCTGATGACGACCATCTTCTTGGACGCACCACCGGTCGCCGGCGAGCCACCCAGCGAAGTGCCATTTGCGTGCAAGGGTGCCGCCGCCGCCAGCGGCGGCACATTCACCGAGGCCTTCTGTCTGTCGACGCTTCATGGCAGCAAAAGCACCGTCGGCGCCGCGGACTACGCGGACCTGGCTCTCGTCGCGGTGGACCTCGCCACGGCCAACGCCACGGCCACCGAGGCCATGATCGACGCCCTTCTTGCTGCCGGCACTAATGGCGCCACGCCCGAGGGGTTGGGGCTGCAGTCGTGCCGGGCGCTGTACGGCGCGGTGGTGCGGCAGTACCAGCCGGAGTGCCGCGCCGCCGTGAAGGACGGCAGGTACGGCGACGGAAAGGCGTGCCTGGGCAGGACGGCGCAGGAGGCCGCGGCGTGCGAGCGGTGGTTCCAGCAGCGGAAGGTGGCGTCGCCGGTGGCACGGGAGGATGACGCCCTCGCCAAGCTCGCCAGCCTCGCCATCGCCCTGGCTTCCATCGCCTGA

>SbPMEI13

ATGGCGGCTTCAACGAGCAGAACATCACCCGGAAACCACAAGGTTCTCCTCGCCGCTCTGCTCTGCATCGCCGCCTTCTTCCCCGGATCATCCGCCAGCACGCCCTTGGTCGCCCAGACCTGCGGCAGGACGTCCAACCAGCGCCTCTGCGTCTCTCTCCTCGAGTCCAGCAACCGGAGCCGCAGCGCGACGACGGTCCGGGATCTCGCCATCATCGCGGTGAGGGGCGCCAGGAGGTCGGTGCTCCGCGCTAGGCTGCGCGCGTGGGACCTCAGCCACGGGGCGCGGCGAGAGACGACGACGCCGGCGGCGGGCCGGCTGGTGGCGAGGTGCGCGGCGCTGTACAGGGACTGCCTCCACGCCGCGGCGCACGCGCTGGCCAGGGTGACCCACATGCCGGCGTACGACGACGGCGGCCGCGTGGCCGCCGACGACGCCGTGGCCGCCCTGCGCGTGTTCCCGGAGAAGTGCCAGCGCCTCTTCGACGCGCAGGAGATCGTGTCGCCGCTGGAGCAGGTGAACCGGGACACGGAGGATAAGCTGCGCGTCGCGTCGGAGATCGTTCACTTGCTGCGCCGCTCTAGACTAGAGCCCCCTTCTCCCTCGCGGCATGGTGATGTCGAACGCACAAATGTCTAG

>SbPMEI14

ATGGCAGCCACTGGGCTATACGGCATGGTCACCTTTCTGCTCCTACTCTCCCTCCCCGCCGCTCTAGCCGATCCCAGCTTCTTCAACCGGACATGCTATCTCACGAAAAACCCCGGTGCATGTCACTCGGTGCTCGGCCACTACTTCTGGAGCCTAAACGCCACCACACTGCCCCAGCTTACTAGCACCGGGCTCGACGTCGCCGTGTTCAAGGCCAGAGACATCATGGGCGCTATGAGCGAGCTCTTTGGAGAGGGGAGGTACGCGGGCACGCACGAAGGGGACGCGCTCGCGCAGTGCGCACGGCTGTACGACAAGACCATGAGCTACGACCTCGACCAGGGGGACATGCGGCTCGTCGACGGGAAGTACAGCGACGCGCTGCGGTTCGTGACCCACGCAAGGAGCGCCGGAGATGCCTGCGAGAAGGCGTTCGCCGACCGCGGCTCGCGGTCCGTGGTGAGCGACTTGAACTAG

>SbPMEI15

ATGGCGACGAGCAGGGCGGCATCACTAGCAAGCCTCAAGAGCGTCTCGGCGGTGGCGGCGCTACTCTGCGCCGCCGCCGTCGCGTCCTCCTTCCTCCCGCTCTCGTCCGCCGGCGTGTCCCTCCTCTCGCGGACCTGCAGCAAGACGGCGCACGAGCGGCTCTGCATCTCCACCCTCGCGCCGGACGGACGGAGCGACGCCGCCCAGTCGGTGCAGGAGCTCGCCGCCATCGCGCTGAAGGTGGCCAGGAACTCCACGCGGGACGCCGTGTGGCGCACCACGGTCCTGGCCGGCGCCAGGGTCCGGACGCCGCTGGAGCGCGACCGCCTGGCGCAGTGCCGTGCTCTGTACAACGAGTGCCTCCGGGAGACGACGAGGACGATAGGCCTGGTGACCGCGGCGAGCTACGACGCCGCGGCGCGCGCCTCCAGCACCCTCCACTGGTACCCGGAGAAGTGCCAGAGCCTCTTGTACAAGCAGGGGGTCGAGTCGGCCATGGAGCAAACGAACAAGCAGGTGGAGGAGCAGCTGATCGCCTCAACGGATCTCGTTCACTTGCTACTTGTTAGGCGGCACGGAGCAGGCAAATTAAACCTAGAGTAA

>SbPMEI16

ATGGCCGGAGCCCGCGTGCTGCTCATCGTCCTCGCGGCTGCCGTCGCGCTCCTGGCCGCACGCCCGGTGGCGGCCACCGGGGGAGTTGCCGGTGTGGAAGAAGTCTGCAGGAGCACACCTTTCCCGGATCTGTGCACCAGGACGGCGGGGAAGCACGCCGAGAAGTACAAGGTCGTGGACGCGGTGACGGTGCTGGAGATGCAGGTGGACGCGTTCAAGAAGCGCGTCAAGGCGGCGCGGAGGGTCGCGAAGCAGGAGGTGAAGACGGCGGCGCCGACGCCCTTGGTGCGGAGGGCGCTGAACCTCTGCAAGAGCTACTACCTGGACGCCGGGGACAACCTCGGCGCCTGCAAGCGCGCCATCGGCTTCAGGGACGCCGTCACCATCCGCGCCACCATGAGCATGGTGGCACAGGACATGCAGAATTGCGACGAGGAGTTCAGGAAGGCCGGCTCCACCAACCCCATGGAGGACCACAACAGGTCGCTCATCGAGATGTCCGAGATCTGCCGCACGCTCTCCAACATGGTCCCTTACGAACATACCCATTGA

>SbPMEI17

ATGGCAGGAGCCCGCACCCTGCTCCTCCTCCTCGTCGCGGCGCTCTGCTTAGCCACCGTGAACGCGGCGACGCTGGCGGAGATCTGCAAGGGGACGGCGTTCCCGGACATCTGCACCAGCACGGTGGGGCCGGAGGCGGCGAGCAACCCGGTGCTGGACCCGATGGCGGTGCTGCGGATGCAGGTGGACGCCTTCAACCAGCGCACGGAGGCGGCGAGGGCGCACGTCAAGGAGGCGGCCATGACGGCGTCCCCGAAGGCGCGGACGGTGCTGGACCTGTGCAACAACCTGTACCTGGACGTGGAGGACAACCTGGGAGCCTGCCGCCGCGCCATCGGCTTCAAGGACGCCGTCACCATCCGCGCCACCATGGGCATGGCGGCGCAGGACATGCAGAACTGCGACGAGCAGTTCAGGCAGATCGGCGAGCCCAACCCCATGGAGCAGTTCGACGCGTCGCTCGTCGAGATGTCCGAGAACTGCCGCTCGCTCTCCAACATGATCTGA

>SbPMEI18

ATGGCTTCGGAAATGTCCTACGCTGCCGTCGGCGTCGTCATCCTCTCCGTCCTCGTCGTCGTCGCCGCGGCGTCCGCAGACCCCACGGCGGCACCTACGGCCGCGCCGTCGTCGAGCAAGCTCTCGCTCGAAGAGGCGTGCAAGCAGACCGCGGGGCACCACGACCTGTGCGTGGCGACGCTGTCCGCGGACCCATCGTCCAAGACGGCCGACACGGCGGGGCTGGCCCGGTTGGCCATCCAGGCGGCGCAGCGGAACGCGTCGGAGACGGCGGCCTACCTCTCCAGCTTCTACGACGACGACAGCCTCGAGAACAAGACGGCGCAGCTGCAGCAGTGCCTCGAGGACTGCGGCGAGAGGTACGAGTCGGCGGTGGAGCAGCTGTCGGACGCGACGTCGGCGGTGGACACGGGGGCGTACAGCGAGTGTGAGGCGCTGGTGGTGGCGAGCCAGGCGGAGGTGAAGCTGTGCCAGCGGGGGTGCCAGGGCGTGCCGGACCACCGCAACGTCCTCACGGCGCGCAACCGCGACGTCGACCAGCTCTGCAGCATCGCGCTCACCATCACCAAGCTCGTCGGCGGACCGCCATCGTGA

>SbPMEI19

ATGGCGGCGAGGCTTGTCTCCGTCTCCGTTCCCCTCCTCCTGCTCGTCGTTGTCCTAGCCGGCTCCCGCGCGGCGCTGGCCTCCGAGACCGTGGACCAGACCTGCGCGGCTGCGCCAAGGCCACGTCGGCGCAAGGAGCACCTGGCGTCCTTCTGCGTGTCTTCGCTCCAGGCGGCGCTCGGCAGCGAGGGCGCCGACGCGCGCGGCCTGGCCGCCATCGCCACCAACCTGACGCTGGCCAACTACACGGCCGCCGTGGCCACCATCAAGGCGCTGGAGCGGCGGGGAGGCTGGCCCGAGCGGTCGCGGCGCGCGCTGGCCACGTCACGTGCCGCCGGCGGTACAATCGAGGCGCTCAACGTCGTGCACAGCGCCGTCCACGCGCTCGCCACGGGGAAGCTCCGGGGCTACGTGTTCGACATGGAGGTCGTCCGGAAGGCGGCGTCCGACTGCGAGGACGCGTTCGGCGGCGCCGGCGGGAACTGCAAGTTGCCGCTGCGGAAGGTGGACGACGACGCTGACAACCTCACCGTGGTGGCCATGCTCATCGTCAGATCCCTCGGCAATTAA

>SbPMEI20

ATGTTCCGATACCTCGCTCCTCTGATTCTCGTCGCCGCCTTGGCGGCGTCTACGACGAACAAGTCCGTTGATGCCCGCGTGGTCCACCCCATCGTCGGCCCCGACACCCAGGCTGACCTCGAGGCCGCCGGCCCATCTCCCGCCGACGACGGCGACGACGGCCGCCGCCTGATCGGCACCGCCGGCGGCGGCCACGATGAACTCGTAGCGCTATGCCAACAGCTGCACTACAAGACATTGTGCACCACGATGACGACGCTGCCCGGGGTGACGACGCCGGAGCAGCTGCTGGACACGGCGCTGCGGATTACAGGGGTGAAGGCGGCGATGGCGGAGACGAAGCTGGACGAGGCGATAAAGTCGTCGGGCGGCGCCCAGGCGGGTAACCCGCTGATGTCGTCGCTGGAGACGTGCAAGGAGAGCTACGCGTCGCTGGTGGACTCCATCAACACCTCGCGGGACACGCTCAAGAGCGGCGGCAGCAACTCGGACCTCATGACGGAGCTGTCCGCGGCGGCCACCTACTCCACCGACTGCGAGGACACCTTCGAGGAGCGGCCGGAGCTCGTATCGCCCATACCCGGGGCGCAGCGCCACATCAGCCGCCTCGTCAGCAACTGCCTCGACCTGGCAGCCACCATTAAGGAGGAGCCCTAG

>SbPMEI21

ATGTCCTATCACACGAAGAAGAAGAAGAAGAAGATGCCACCACCACCATGCTATTGCTCCTCCCTGATAACAATAACAATAATCCTCCTCCTACAACAACAGAACAATCCCTGGACTACAGCTGCAGCAGCTTCAGCCATGGCCACCACCACCACCAAGCTTGGGTCGTCACCGTTGTCTGACGTGGTAAAGGACACCTGCGAGAGATGCAGGCAGGGCAACCCACAGGTGAACTACACCCTCTGCGTCTCGTCTCTGTCGTCGGACCCCAAGAGCAGGCAGGCGGACCTCCACGAGCTCGCCATGATCTCGGCCAAGCTGGTGAGGTCAGGCGCAGTGGGCATGGAGGCCAAGATGGCGGAGCTCAGCAGGAAGGAGCGGCCATGGTCTCGCCGGAGGTCCTGCCTGGAGGCGTGCATGGGGGTGTACCACAACTCCCTCTATGACCTCGACGCCTCCATCGCAGCCATCCAGGAGAGGAGGTATGCGGACGCCAAGACGTCCATGAGCGCCACCGTCGACGCGCCCATCACCTGTGAGGATGAGTTCAAGGAGCAGGGCCTGGAGCCACCCATGAAAGCAGAGAGCAAACGCCTGTTTCAACAGGCGGCCATCACCCTCGCCATCATCTCCCTTCTATGA

>SbPMEI22

ATGGCGATGATGGGTGCTACGACGATTCGTGGTGGTGGTGCTAGCAGCAGCGTCCGGCCTGCTGTGCTGGTCCTCGGCCTCTGCCTGCTGCTGCTGCTGCTGCTTGGTGTTGCGCAGGCGGTGGAGTTTGAGTTGGTGCCTGATGCTGGTGCCATTGATCCGATGATGATGCCTGCCATGGACGACGTCGACGGTGGTGGTCGTGAGCCGCCGCGCGAGTGCATGACACCGGTGAGCGTGGAGGAGGCGTGCCGCGGCGCGTCGGAGCTGCACGCCGGCGTGGACTACGACCACTGCATGGCGTCGCTGGGTGCCGACCCGCGCAGCAAGGAGGCCGGCAACAAGAACATGCACGGGCTGGCCGTGCTCGCCACCAAGATGGCCATCGACCACGCCGCCAGCACCGAGTCCAAGATCGACGACCTCGCCGAGCTGGATGCGGATAACCAGTCGTCGTCGCCGCAGGCCCGCCGCGCCCGCTTCAACCACTGCCTCGAGCAGTACGGCGGCGCCGCCGACCTCCTCCGCGACGCGCTCGACAACCTCAAGGCCAAGATCTACGGCAAGGCCATGGAGCAGCTCACCGCCGCGATGGGCGCCTCCGAGAGCTGCGAGGACGCCTGGAAGGGCGAGGAGGAAATCCCCGTCGCCGCGCACGACAGGGAGTACGGACGGATGGCGCACATCGCCTTCGGATTCACACACGCAGCCGCCGCATGA

>SbPMEI23

ATGTCGAGGGCTCTCCTGATGGTAGTAGCCCTGGCCGCCGTCCATGGGCTCATCACGCTCACCGGCGTCGACGCGACCGTGGTAGCGACATGCCTGGCGGCGTCCAACAGCGACCGGCGCGTCAACTACGACTTCTGCGTGTCGGAGCTGAACAAGCACCGCGACAGCCCGGGCGCCGACACCCCGGGCCTGGCCAAGGTGGCGGCCAACGTCGGCGTCAACAGCGCCGGCGGCGCGGTCAACGACATAGAGGCCCTGCTCGCCGCCAAGCAGCAGCCGCCGCCGGACGCCAGGACCAGCGCCGCGCTGCGCCTGTGCGAGCAGCTCTACTACGACATGGAGCTCGCCTTCGCGGGGGCCTACGACGAGATCAACGCGCTCAACTACACGGCGGGGAAGCAGATGGCCGCCGACGCCGACTCGCTGGTGCGCCGGTGCACCGGCGGCTTTGCCGAGGCCGGGCTCGTGCCGCCGGAGCCCGTCGCGCGGCGCAGCGCGTACGCCGTGCAGATCGCCATCGTGTGCACGGCCATCACCAACCTCATCATCAGTCCATGA

>SbPMEI24

ATGAGGCCTTCGACGGCTCGGGCTCTGGCCACCGCGGCCATCGTCGCGGCGCTCGCGCTGAGCGCCGACGTCGTCGGCGGCACCCCGGAGACGACGTGCGCGGCGGCGGCGGCCCACGACCGGCGCGTGGACTACGGCTTCTGCGTGTCGAGGCTGAGCCACCACCACGACAGCCCCGACGCGGACACCTGGGGCCTGGCCAAGGTGGCCGCCGACGTGGGCGTGGCCATCGCCGGGGACGCCGTCTACGACATCAAGGCGCTGCTGGCTACCAGCAGCAAGCCGCCGGGCGAGGGCGAGGGCGACGCCCAGGAGCGGGCGGTCCTGGAGCAGTGCCAGAGGCTGTACGACGCGGCGGAGTCGGCGTTCGCGGAGGCGTACGACGCGATCAACCGGCGCGACTACGCGGCGGGCAAGGGCAAGGCCGCCGAGGCGGCGTCCCTGGCGCGCCGGTGCGACGACGCCTTCGCGCGCGCCGCACTGCGCCCGCCGCCGCAGGTCGCGCGCTGGGGCGAGGAGTCCGCCAAGATCGCTGTCGTCTGCACGGCCATCACCGACCTCATCGACTGA

>SbPMEI25

ATGACGATGAGGCCGCTGCCGCAGACCGTCGTCCACCTCCTCCCCCCCGTCGCCGCCGTGCTCGCGTTCGCGCTCATTGGCTGCCTCGTGGGCGGCGCCAGCGCGACGGTGGTGACGACGTGCAGGGCGGCGGCGGACAGCGACGCGCGCGTGGACTACGGCTTCTGCGTGGCGGAGCTGGGCATGCACCGCGAGAGCCCCGACGCCGACGTCTGGGGCCTCGCCAAGGTGGCCGCGCTGACGGGCGTCAACAACGCCGACAACGCCGTGTACGACATCAAGGCCCTGCTGCTGCTCGCCGCCGACGACGGCGCCAAGAGCAGGTCGCCGCCGCCGGACGGCCCGACGCGCGCGGCGCTGGAGAAGTGCGGGAGGCTGTACGACTCCGTCGGGTTCGCGTTCGCCGAGGCGGACGACGAGATCAACAACCGCCGCTACGCCGCCGGGAAGGGCAAGGTCGCGGAGGCGGTGTCCCTCGCGCGCCAGTGCGACGACGCCCTCGCCAAGGCCGCCGCCGTCCCGTCGCCGCTGGCGCAGCACAGCTCGTACAACGTGAGGATTGCTAATATCTGCACCGCCATCACCAACCTCATCAAGTGA

>SbPMEI26

ATGAGTCCGTGGAAGACCCTCTTGGTGGCCGCCGCGGCCCTCGCCACGCTCCTGGCAGCCGACGCTACCGTGGAGTCGACGTGCAAGGCGGCGGCCGCCATGGACGTCCGCATCGACTACGGCTTCTGCGTGTCGGAGCTGAGCAAGCACCGCGACAGCCCTGGCGCGGACACCTGGGGCCTGGCCAAGGTGGCAGCCAACCTCGGCGTCAACAACGCCGGCGGCGCAGTCCGCGAAGCGGACGCGCTGCTGGCCAGGCCGCCGGGCACGGGAGGCGCGGACGACGCGAAGGCAAGGGCGGCGCTGGGGCAGTGCCGCAGGCTCTACTTCGACATGGAGCTCGCGTTCGCGGGGGCGCACGACGAGATCGACGCGCGTCAGTACGCGGCGGGGAAGGAGATGGCCGTGGAGGGCATCCCATTGGCGCGACGGTGCGACGCCGTCTTCGCCGAGGCCAGGATCCCGTCGCTGCTGGCACGGCGAGGAGAGTACGCCGAGCAGATCGCGGTGCTGTGCATTGCCATCACCGACCTCATCAAGTGA

>SbPMEI27

ATGAGTCCATCGAGTATCCTTGTCACCACATCCGCCATCGTCGCCATTATTCTTGTTCTTCATGGTGCCGACGCTACGGTGGTGACGACGTGCAAGGCGGCTGCCGAGAGCGATAAGCGCGTCGACTATGATTTTTGTGTGCTAGAGTTGGGCAAGCACCATGAGAGCCCCGATGCAGATATTTGGGGCCTAGCAAAAGTGGCGGCTCTGGTCGGTGCTGCCAACACTGGGAATGTTCTCGTCGAGATCAGGGCCCGGCTAGCGAAGCCGGGAACAGACGCCAAGACGACGACGGTGCTGCGACAGTGCCTTAAGTTGTACGACGCAGCAGATGACGCGTTCCTGAACGCCTATGAAAGAATCAACGAACGCAATTACGCCGCAGGGAAGGAAGAGGTGAGATGCGATGACGCATTCACTAAGGTTGCCAGCCCATCGCCTCTCAACCAGAGCAGCACGTACACAACGAAGATATCCATCGTCTGTATAGCAATCACCAACCTCATAAAGTGA

>SbPMEI28

ATGAAGCTAGTCTCCTCCGTGTTGTTCGCCCTGCTCATCTTGCCGATGTGCAGATCTTCCCCGCTTCAAGACACATGCAGGTCCTTCGCCGCCGGCCACCCGTCCATCGGCTACGACTACTGCATTAGGATCTTCCAGGCCGACAAGGCCAGCGCCGAGGCCACCGACGCGCGCGGCCTCGCCGCCATCGCGGCCAGGCTCGCCGAGGCGAAGGCCAACGCGACGGCCGCGCGCGTCGCGTCCATGAGCGCGCTCGAGGGGGACGCGAGGAGGCGGGACCGCCTGTCCGTGTGCGCGGAGGTATACTCGGACGCCGTGGACCAGCTCGACCAGGCAGAGGAGGAGCTCGCCCACGGCGCGGAGGGCGGCATCGACGACGCGGTCACGCAGCTCAGCGCGGCGCTGGACGCGCCCGAGACGTGCGAGGACGCGTTCCGCGAGGCCGACGACACGTCGCCGCTCGCCGCGGAGGACGCCGAGTTCAAGAAGCTGGCGACCGTCGCTCTCGCCGTCGCGGCGTCACTGACGCCGCCACCAGCATGA

>SbPMEI29

ATGAGGTCATTCCTGGTGCAACCTGTATCCATACTACTACTACTACTCTTCATCACAGCCATCGCTCCCGTGGTCACCGCCGGCGGCTCACCTGTCATCAACGCGACGTGTGCCGCGCTCAAGTCCCTGCAACCCTACGACTACTGCGTGGGCGTGCTCTCCGCCGACCCAGCAGCCGCCGCTGCCACAGACGTCCGGGGAGTGGCGGCAGCCGCCGTCAACATCACCGCGCAGAAGGCTGCGTCCACGTTGCTCGTCATCAACTATCTCGCCGGCGATCTCAACACCTGCCGCGGGTACTACAGCAACATGTTGCAGTCGTTGGAGAATTCCCTCGTCCACTTTCGCGATGGTCGATTCTTGAATGCGTCCCTAGGGATCGCCAATGCCACCGGAGATCCCACGGGCTGCGATTTACTGCTGTTCGAGGGGAAAACGCACAAGGATCCGATTTCCGACGAGAACTATGAGAACATGCGCTTGGTTGACCTAGCAGATGGTATCGTAGATCTATTCGCGAATAAGCGTCTATACTAG

>SbPMEI30

ATGGCCTACTACATCAAGAGCTCAGCAATGGTGCCCGTACTACTCCTCGCCGTATTAGCCATCGCCCCAGTGCTGGCCATCGCGACCACATCTGCCATAAACGCGACGTGTACCGCGCTCGACGCCCAGCATTACGACCACCCCTACGCGTACTGCGTGGGCGTGCTCTCCGGCGACTCGGCGGCCGCCGCCGCGACGGACGAGCGTGGGGTCGCCGCAGCCGCGATCAACATAGCAGCGCATAAAGCGGCGGCGACCGTGAGCGTCGTCACCTACCTCGTCGACGAGCTTAGCCTTTGCAGCAAATACTACGGCCGCATGGTGGAGTCGCTGACCGCCGTCCTGGCGGACTTCCACGCCGGACGATTCGACGATGCGGCGCTTGCGAAAGCGCGAAGTGCCTCTGAGGTGCCCAACGACTGCGACGTCATCCTGTTGCAGGGCAGCGCCAAGAAGAACCCGTTTTCTCAGGAGAACATCGACAACGGCAGGTTGTCAGGCCTAGCCCGTGACATCACTGCTCTTGTTGCGAACAAAGGCCCGTCG

>SbPMEI31

ATGGCCATGGCAGCAACCCCAACCGCGACTGCCAGCATCTTGCTCTTGGCTCTCTTCCTCGCCGGCGCTCACGCGGAGCCGGCCGAGCTCCCATGCGCGCTCCCCGCGTGCAAGACGGTGGGCGGCGGCAGCCAGTTCTTCGACGTGCAGTTCTGCTTGGCGGCACTCGGCTCCGACGGCCGGAGCATCAGCCACTGCATGGACTACCAGGTCTACTCCGTCATCGCTGCCGACCTCCTCGCAGCCAACGTCACCGCCACGGCGGCCAAGATCGACGGCCTGCTCCAGGGGAGCGGCGGCGGCGGCGGCGGAGACGACGCCGCCGCCACGGCGCGCTGCCTCCGGTCGTGCCAGGCGCTGTACGGCGGCACGGTGCGGAGGCAGCCCGGCTGCGCGGCCGCCGTCAGGGGCGTCAGGAAGGGCGAGGCCACGACGTGCCTGGAGGAAGCCGCCGCCGCGGCCAAGCAGTGCGAGGACGGGTTCCAGAGCAGCAAGGTGGCGTCGCCGGTGACGGCGGAGAACCAGAACGCGTTCATGCTCGCCAAGCTCGCCGTCGCGCTGCTCCGCGAGGTCTATGCTAATAAATGA

>SbPMEI32

ATGGCGGCGCCGCAGCCTCGAGCCCTCACTACCCACCACCACCACCTTCTCCTCCTCCTCCTCATCGTCGTCTTCACCATGGCTAGTGCGCACACGACAGCGGCACCCGCACCGAGGGCGGCGGCGGCGGCGGCCGAGAGTCCATCGCCAGCGGCGATGTCCTTCCTCCGTGCCCGCTGCGCCACCACGCTGTACCCGGCGCTCTGCTACGACTCCCTCCTCCCGTACGCCTCCGAGGTCCAGGACAACCCCGCCCGTCTCGCGCGCGTCGCCGCCGACGTCGCGGCGGCGCGCCTCCGCGCCCTCTCTGCCCGCGTCAAGGACATCCTCCGGCACGTCGGCGGCGATCCAGCAGAGGGAGCCGCCGCGCTGCGCGACTGCGCGAGCACGGTCTCCGCCGCGGCGAGCCTGGCGAGGCAGTCGTCGGCCGAGCTCACCAAGCTGGAGCCGGACGCCGGTCGCGTCGTCACGACTTCGGCAGGCGACGGGATGAGCAGCAGCAGGCAGGCCAGGTGGGAGGTGTCCAACGCCAAGACGTGGCTCAGCGCCGCGATGGCCAACGAGGGGACGTGCGCCGACGGGCTGGTGGAAGCTGGCGCCGCGGCAGCCGCGGGGAAGGAGGTCACCGCCGGCGTGGCGGCCGTGAAGCAGTACACCAGCAACGCCCTCGCGCTTGTCAATGGCATTCCACTGTGA

>SbPMEI33

ATGAGGGTGCCTCTCCTCCCCTTCGTCCTCATCGTTGCCGTCGTCGCCACCACCGTGTCCCTGGTCCCGGCGGTGTGCAACGGTCAAGAAGCTGCTACCGCCGGCGAGGAACATGGAGGCTCTATCAAGCCCTTGTCCCTCGACGGATATGGACCACTGGAGAAGGCTGCCAAGAAGCCCAAGGAGCAGACCTTGAACGCGCAAGCTTCGCCGGCGGTGCCCGCCGACACCTATGATCAGAAACCTGACAAATATGTTGCGTCGTCTCTAGTTCCTGCTAAGGAGGAGGAGGAAACTCCTGCGGAGGTAAAGAAGGAAAAAAAGGAGAAGTCGGATTATTTGGATGAATCTACATCATCCAAGAAGGAAAAGAAGGAGAAATCTGATGATTCTGATGTATCTACATCTTCCAAGAAGAAAAAGAAGAAGGCCAAGACTGACGATTCTGACGAAGATGCATCTCTTACTAAGAAGGAAAAGAAAGAGAAGAAACACAAGAAGCACAAATCCGATGATGATGATTTGGACAGTACATCTCCCAAGAAACACAAGAAGGAGAAATCCATTGATTCGGATGCATCTTCATATCTTCAGAAGGAAGAGGAGAAATCCGGCGGCGATTCGGACGAAGCCACGTCTCTTAAAAAGCACAAGAAGGAGAAGAAAAACAAGAAGAAGAAGGAGGAGAAATCCGGTGAGAACGCCGACGAGGACGACGCTGCGCCAGTCGACGTCTCCACGGACGGGCAATATGTGTCTCCCTCTTCCAAGGAGGAGAAATCCGACGAGGATGACGCCATGCCGGTCGACGTCTCCACCACCACCGGCCAATACGTGTCTTCTCCGAAGTCCAAGGGAGGAGAGCGTCAGGTCTCCACCCCTACGGATGCATACACGTCTCCCGACGAGCTTCCTCCGGCGGCCAAGAGCTCCACCACCTCCGATGCATACGCACCTCCAAAGCAACAGGTCGTCAGCAGCAGCAGCAGCAGCAGCCAGCCCATAGCCGGTGGTTCTCCCGACGAGCTTCCGCCGGCGGCCAAGAGCTCCGCCACCGCCGACCCGTACTTATCTTCAAAGCATCAGGTCGTCAGCAGCCAGCCCATGGCCGGTGGCGCTCCCGACGAGGTTCCGCCGAACGTGGCCGCCAACGGGCAGCCGAACTTGCCGGCGGCGGGGAATAAGCCGAAGCTGTCGATGGGGACGTTATCGGGGATGATCAAGAAGCCGATCGCCAAGTTCCTGAGCCCGGTGATCAAGAGCGTGTGCGCCAAGACGGAGTACCCGGTGGACTGCGAGGCGTCGATCGGCGGGCTCCCCGGGGCGGCGTCGGCGGCGGCGACGGACAGCGTGGGCGTGCTGAAGCTGGCCATGGAGGCGGTGCGGCAGAAGGTGATCGTGGCGATGAACGCGGCGACGGACCGGATGAACGCGCCGGGCGTGGACGGGACGACCAAGGACGCGCTGGACTCGTGCACGTCGTCGTACAGCGACATCAAGACGAGCTTGGACTCGGTGGACGACGCGCTCAAGCGCGGCGACGTCGACACGGCGCACACCAACCTCGACTCGGTGGAGACGGACCTCACCACCTGCGACGACGGCTTCCAGGAGCACGGCATCCCGTCGGTGATGACCGACCATGACCAGGAGCTCCAGAAGCTCGCCAGCAACCTCCTCTCCATCGGCGCCGCCATCCATCGCTAG

>SbPMEI34

ATGACGCTCCCGCGCCGCCGCCACCTTGTCCTGCTCGCCGGCCTCCTGGTCCTCGTCGTCGTCGCGGCCACCGCCACCGCGTCGACGGATTCCTCTTCCACCACGGCGGTGGCGGTGGACTTCGTGCGGCGGTCGTGCCGGTCGACGGAGTACCCACGGGTGTGCGAGACCACGCTGGTCCCCTGCGCGGCGAGCGTGGGTCGGAGCCCGCGGCGGCTGGCGCGGGCGGCGCTGGTGGTGGGCGCCGACCGCGCGCGCAACTGCTCCGCCTACATCCACGGCAGCCGCAGCGGCAGCGGCGGGGCGATGAAGGACTGCGCGGAGCTGGCGCGCGACGCGGAGGACCGGCTGCGGCAGTCCGCGGCGGAGATGGAGCGGATGGGCCGCGCCGGCACCCCGCGCTTCGCTTGGTCCCTCAGCAACGTCCAGACCTGGGCTAGCGCCGCGCTCACCGACACCTCCACCTGCCTCGACTCCCTCGCGCAGCACAAGGACCGCGGCCGCGGTGGCAAGGGCAGCGACGGCGACGACGATGCCGTCAGGGTGAAGAGGAGGGTGGTCGCCGTCGCGCAGGCCACCAGCAACGCGCTCGCGCTCGTCAACAGGCTCCAGCCGGCGACGCACCGGCAGAGGCTCCTCCTGTGA

>SbPMEI35

ATGGCAGCGACAAGGGCATCCTCGTTGCTGATCTTGCTTCTCATCATCCAGCTGAACTTGCTCTTCCACCTCCCAGCTGGCTCCTCCTCCGTCACCGCCGTCGCGCAAGCAGCTGATGAGCAACAGAACACCAAGCAGCAACACCGCCAGCCGGCGCTGGTGCAGTCGACGTGCAACTCGACCAGCTTCTACGACGTCTGCATCGCGGCGCTCGCCGCGGACCCGTCCAGCTCCACGGCCGACGTCCCGGGCCTCTGCGCCATCGCCGTCTCCGCGGCCGCCGCCAACGCCTCCGGCACGGCGGCGTTCCTCGGCAACGCCAGCAGCGACGCCGCCGCGGCGGCGGCGGGCACCCCGGAGGCGGCGGACTACCGCGCGCTGCTCCGCGCCTGCGCCGGCAAGTACGCGGCCGCGCGCGACGCGCTGCTGGAGGCGCGGGCGTCCCTCGCCCAGCAGGCCTACGACTACGCCTTCGTGCACGTCAGCGCCGCCGGCGAGTACCCCGCCGTGTGCCGGACGCTGTTTCGGCGCCGCCAGCAGCGTGGGGGCAGCAGCAGGCCGTACCCGCCGGAGCTGGCCAAGAGGGAGGAGGCGCTGCGGCGGCTCTGCACCATCGCGCTCGACATCATCTCGCTGCTGCAAAATCAGGAGCCCAAATAG

>SbPMEI36

ATGAAAGAGAATCCTCTGTCCAAAGTTTCAACATCCCAACATGGCTACAAAAACCCATCCAAAGCTTGCAGGCAAATTAAGCGGCAACGACACACCAACCTGGCACTAGCAGTAGCAGCACACGCACCCTCCCAGCAAGCAACGATGGCGGCGAGGCTTGTCTCCGTCTCCGTCTCCGTTCCCCTCCTCCTGCTCGTCGTTGTCCTGGCCGGCTCCCGCGCGGCGCTGGCCTCCGAGACCGTGGACCAGACCTGCGCCAAGGCCACGTCGGGCGCGCAGCACAAGGAGCAGCTGGCGTCCTTCTGCGTGTCGTCGCTCCAGGCGGCGCCCGGCAGCGAGGGCGCCGACGCGCGCGGCCTTGCCGCCATCGCCACCAACCTGACGCTGGCCAACTACACGGCCGCCGTGGCCACCATCAAGGAGCTGGAGCGGCGGGGAGGCTGGCCCGAGCGGTCGCGGCGCGCGCTGGCCACGTGCCGCCAGCGGTACATCGAGGCGCTCAACGTCGTGCACAGCGCCGTCCACGCGCTCGCCACGGGGAGGTTCCGGGACTACGTGTCCGACATGGAGGTCGTCCGGAAGGCGGCGTCCGATTGCGAGGACGCGTTCGGCGGCGCCGGCGGGAACGGCATGTCGCCGCTGCGGAAGGTGGACGACGACGCCGACAACCTCACCGTGGTGGCCATGCTCATCGTCAGATCCCTCGGTAATTAA

>SbPMEI37

ATGAAGCTCCTGCAAGCTACTGTCTCCCTGGTCTTCCTCCTCGCCTGTTCCACGTCCAACGCTTCTGTCTTACATGACGCCTGCCAGTCCTTCGCCGCTATCCGACACAAGGATGCCGACTACAACTACTGCGTCAGGTTCTTCCAGGCCGACAAGGAGAGCGCCACCGCAGACCACCGTGGCCTCGCCGTCATCGGAGCCAAGCTCATCGAGGCAACCGCTAAGAGCACCGGCTCTCTCATCGCCACCATGCTGACCTCGGAGAAGGATAAGGAGAAACTCGGTTGCCTCGTCGCGTGCGGCAAGGGTTACTTGGACGCCATGGACGAGATCGGCAAGGCGGCGAAGGGCATCGTCTCGAGAAAGGATGGGGGCGTAGAGGACGCGGTGACGGCACTCGGCGGGGCGCTGGACGCGCCCTTGGACTGCGAGGATGGGTTCCAGAAGCTTCACAAGCCGTCGCCACTCGCTGCGGAGGATGCCAGGTTCCGCAAGGAGGCGTCCATCACCCTGTTTGTAACGGGGACGTTACTTCCCCAAATCAACAGCTCTAAGTTAGGGATCTAG

>SbPMEI38

ATGAAGCTTCTGCAAGCTCTGTGCCCTCTCGTCTTCCTCCTCGCCTGCTCCACGTCCAACGCTTCCGTCTTACAAGACGCGTGCAAGTCCTTCGCCGCTAAACACCCGGAGACCGGCTACTACGCCTACTGCATCAAGTTCTTCCAGGCCGACAAGGGAAGCGGAAGCGCGGACAAGCGTGGCCTCGCCGCCATCGCCGTGAAGATCACCGGGGCAGCCGCCAAGAGCACCGCCCAGCACATCGCCGCCCTGCGGGCCTCCGAGAAGGACACGAAGCGGCTGGCGGGCCTCAAAGATTGCTCCGAGGTGTACTCGCAGGCCGTGGACCAGACCGGCGTGGCGGCGAAGGGCATCGCGTCGGCCACGCCCCGGGGCCGTGCGGACGCGGTGACGGCGCTCAGCGCGGTGGAGGACGCCCCCGGCACCTGTGAGCAGGGGTTCCAGGACCTGGGTGTGCCTTCGCCGCTGGCCTCGGAGGACGCCGAGTTCCGGAAGGAGGCGTCCATCGCGCTGTCTGTAACGGAGGCGCTGTAG

>SbPMEI39

ATGGCAGCAGCAGCCACCACCAAGAACGTCATGGTGCTCCTCCTCCTAGCACTCCTCCCTCTCGCCACACTCTCCTCCCGCGCCGGCCCATCATCGGCTTACAAAAGCCACGGCCACAGCCACAGGTCATCGCCGTCGGCCAAGCACCCACCGCCGTCTCCTTCTCCTCCGTCCTCACCACCTTCTGCTCCCTCGCCTGCTCCGGCGGCGACCGCCGCGCTAGTGCGCGCGACCTGCAACTCCACGGCGTACTACGACCTGTGCGTGTCCGCGCTGGGCGCGGACCCGTCCAGCGCCACGGCCGACGTGCGGGGCCTGTCGGCCATCGCGGTCTCGGTCGCGGCGGCCAACGCCTCGGGCGGCGCGGCGACCGCCGCGGCGCTGGCGGCGAACGGCACCGCCCCGACGGGCACCGCCGCCGCTGCTTCCAGTTCCACGGTCGTCGACGGCACCGTGCAGGCGCTGCTCCACGCCTGCGCGGCCAAGTACGCCAGCGCCCGCGACGCGCTGGCCGCCGCCGGAGACTCCATCGCGCTCGAGGACTACGACTTCGCGTCCGTGCACGTCAGCGCCGCCGCCGAGTACCCGCAGGTGTGCCGCACGCTGTTCCGGCGGCAGAGGCCAGGACAGTACCCCGCGGAGCTCGCCGCCAGGGAGGAGACGCTGAAACAGCTCTGCTCCGTCGCGCTCGACATCATCGGGCTGCTCTCAAACAGCAGCTAG

>SbPMEI40

ATGGCGGCCAGCACCAGGAAGGCAGCACTCGTGCTAACCCTAACAATGGCGCTCCTCGCTCCGAGCATCCTCGGCGCCCGCACCAGACCGCCGTCGTCTCCGCACCATAGCCAGGGCCACAAGCGCTCGCCGCCTCCCGCTTCTCCTCCTCCGCCGCCGGCGCCCGCCGCACCCACGGCCGCCGCCGGACTCGTCCAATCCACGTGCAACGCGACGGCCTACTACGACCTATGCATGTCAACGCTGGGCGCCGACGCATCCAGCGCCACGGCCGACGTGCGAGGCCTCTCATCCATCGCCGTCTCCGCCGCCGCCGTGAACGCCTCGGGCGGCGCGGCCACGGCCGTCGCGCTGCTGGCCACCGCCGGCGCCGGCGGCGGCAACACCACGACCGCCGTCGACGGCACCACGCAGGCGCTGCTCCGCACTTGCGCCACCAAGTACGGCGAGGCCCGCGACGCGCTGTCGGCCGCCAGGGACTCCATCGCGCAGGAGAACTACGACTACGCGTCCGTGCACGTCAGCGCCGCCGCCGAGTACCCGCAGGTGTGCCGGGTGTTGTTCCAGCGGCAGAGGCCTGGGGAGTACCCGCCGGAGCTGGCGGCCAGGGAGGAGGCTCTCCGACGGCTTTGCACCGTGGCGCTAGACATCATCACGCTCCTCACCAACAACACCAACTGA

>SbPMEI41

ATGGCAATGGTGGTGCTCCTCCTCTTGTCCCTGCTCCCTCTGAGCACCCTCGGCTCTCGCTCCGGCCCGACGCCGGCCGTGCCGCACCACGGCCACGCCGGCCATGGCACCCCCAAGCACTCCTCGCCGCCTCCCCAACCAACCACGGCGGAGCTAGTACGCAGCACCTGCAACTCCACAGCCTACTACGACCTGTGCGTGTCCGCGCTGGGCGCCGACCCGTCCAGCGCCACGGCCGACGTCCGCGGGCTCTCCACCATCGCCGTGTCCGCGGCGGCCGCCAACGCCTCGGGCGGCGCCGCCACGGCCACGGCGCTCGCCAACGGCAACGGCACCGCCACGTCGTCCAACGCGCAGGCGGCGGCCCCGGCGGCCACCGCCGCCACGGCGCTGCTTCGCACGTGTGCCGCCAAGTACGGGCAGGCCCGGGACGCGCTGGCCGCCGCCGGTGACTCCATCGCGCAGCAGGACTACGACTTCGCGTCCGTGCACGTGAGCGCCGCCGCCGAGTACCCGCAGGTGTGCAAGGCGCTGTTCCGGCGGCAGAAGCCCGCCGGCGGGCAGTACCCGACGGAGCTGGCGGCGAGGGAGGAGGCGCTCAGGCAGCTCTGCTCCGTCGCGCTCGACATCATCGCGCTCGCTTCCAACACCAGCAGCTAG

>SbPMEI42

ATGGCCTACGTCGCTGCAGCAGTGTTGGCAGCAGTGTCTCTCACCGCCTTGTTGTTCGCCGGCGGCGAGGCCTGCGCCAATGTCCCGTCCATGACATCGACCGAGGCGTGCCAGCAGACGAACAAGTGGGAGCAGCTGTGCCAGCAGACGCTCCAGACGGCGCCGGACACCGCCGAGGTCACCGTGTTCGCGCTCGTCGCGACGAGGCTGGCCAAGAGCGCGTACGAGGACACCTTGTCGGCGCTGGACCAGATGCTCGGCCCCGGCAACCTCCCCGGCGCTGAGCGGTTGGCCATCGACAACTGCAAGGAGACGTACAGCACGGCGCTGAGCAAGATGGCCGGCGTCGTGGACCACATGTCCGCCTGCGACTTCTCGCTCGCCAGCAAGGAGTACATCGACGCCGAGGCCGGCGTCCGTTCATGCCTGGAAGGGCTGCAGCCGTACCAGTTCTTGCCGCTGTTCGGCAAGGTCTCTGCAGACCATGATTTGACCTTGGTCGCGTATTTGCTAGGCGCTATCATTGTTGGCAGGTAG

> SbPMEI43

ATGGCCTACATCGCTGCAGCAGTGCTGGCAGTACTAGTGTCTCTCACCGCCTTGTTCGCCGGCGGCGAGGCCTGCAACAACGTCGCCTCCATGACATGGACGGCGGCGTGCCAGCAGACGGACAGGTGGGAGAAGCTGTGCCAGCAGACGCTCCAGGATACGGCGCCGGACACCGCCGAGGTGACCGTGTTCGCGCTCATCGCGACGAGGCTGGCCAAGCTCAGGTACGAGAACACCTTGTCGGAGGTGGACACGTTGCTGCGGCCCGGCAACGCCCCCGCCGAGTCGCGGGCGGCGCTTGACAACTGCAAGGTGAAGTACGGCTCGGCGCGGCGGCTCTTGGCCGGTGTCTCGGACCAGATGTTCGCCTGCGACTTCTCGCTCGCCAGGCAGGAGTACATCGACGCCGAGGTCGGCGTCCGTTCCTGCCAGGATGGACTGCTGCGGCTGCCGAACCAGTACCAGAGCTGGCCCTTGTTCCGCAAGGTCTCTGATGATCATGAGTTGACTGTAGTCGCGTATTTACTAGGCGCTATCTTTCTTGGCAGGTAG

>SbPMEI44

ATGACGCCGCCGTCGCCGCCGCCGCCGCTGTTCCTCGCCTGCATTCTCCTCACCCTCCTCCTCGCCGCCGCGGTGGCGCCACCGGCGGTCGCCGTCTGCGTTCCGAAGGGTCACAGCAAGCCAGGGGCGCCGGCCAAGGCGAAGGCGAAGGCGAAGCCCAAGCCGAAGCCGGCGCCGCCGAAGCCGACGGCGATCCCGATCGCGCCTGGCGCCGACATCGTGCGGAGCCTGTGCGTGAAGACGGACTACCCTGACCTGTGCACGTCGGCCATCACGAAGCAGCCGCAGCCGCAGCTGCCCGCCGGGAAGCGGCTGGACGGCGCCGGCGTGCTGCGGCTGGCCATGTCCGCCGTGCGCGCCAAGGCCGCCGAGGCCAAGGCCGCGGCGGGCGCGCTCGCGAAGGACCCCAAGACGCAGCCGCTGGCGCGCAACCCGCTGCAGGACTGCGTCGAGTCCTTCGACGACATCGCCTACAGCCTCGACCAGGCCCAGAAGGCGCTCGCCGGCGGCGACCGCGACACCACGGGCACCATGCTCGACACCGTGCGCACCGACGTCGACACCTGCGACCAGGGGTTCGAGGAGCGCAAGCAGCTCACGCCGGTCATGTCCAAGCACGACGCCGAGCTCGCCAAGCTCTCCAGCAACTGCCTCGCCATCGCCACCGCCGCCGGCTTGCGCTAG

>SbPMEI45

ATGGCCGTCGTCTGCGGCGGCTCCAGCGCCGCCGTCGGGGCGGCGAACTCGGCGCTGGACCAAGTCTGCGAGTCCGTTGGTGGTTCCTACGTCACGCCGGAGCTCTGCGCCTCCGCGCTCTGCTACGACGCGGCGTCGCCGTGCCGCGACGCGCGCGACTACGCGGCGGTGGCGACGCTCGCCGCCGGGCTCCTGGTGCGCAACGGCACCGCGACCAGGGACACCGTGGCCGTGGCCGCGGCGGCGGCCAACGCCACCGCGGGCCTCAAGTCGTGCCTGCAGCTGTACGACGGCCTCTTGCCGGCGCTGGAGTGGGCGGCGGGGTCCGTGGCCGCGGGGCGCGCGTACGGCGCCGCGCGGGAGCTGATGCAGGCGACGCAGTTCGCGCAACGGGCGTGCGCTGGCATGGTCGCCGGCGCGGAGATGCCCAGGGAGAACGGCGGCTTCGTCACGATGGCCAGAGTCGCGCACGCCGTCCTCTCCACCTCCGTTCCAAAGACTGACTGA

>SbPMEI46

ATGAGGACCTTGCTTGTCGCCGCCGCCGCCGCGGCCCTCATCCTGTCCGCCGCCGCCGGCGCGCTCGGCGTCGCGCCGGCCGACACCGTGGCCGACTCGTGCCACGCGATCAGCGACTTCGTGGACATGGACTTCTGCACGTCCCGGCTGCGGTCCGTGCCGGGGGCGGCCGCCGCGGACCGGTTCGGCCACCTCCTGATGGCCACGGACCTCGCCGTGGCGAGCGGCGCCAAGGCGCAGGACCTCGCGGCGGCGGCGGCGCGCGACGATGGCAGCGTCGTCGTCGACCCGGGCGAGCGGGACGCGATGCAGGCGTGCGCGTTCCTGTACGGCGCGGCGTCCGTGCCGGGGCTGCGGCTCCTCCGCCAGTACGCGGCGGCGCGGAACTGGGCCCCCGCCCACGCGCTGGTGATGCTCACCATGGACGCCGGCGACGGGTGCGACGCCGCCGTCGGGGGATCTAATGGCAGGATGGCCGGGCCGAACCACGAGTTCGACCAGCTTAGCGCCATGGTCACCGCGCTGCTCAACAGCATCAACTTGTACGATGAGTAG

>SbPMEI47

ATGGCGGCCGGGTCGTCCTCCTGCTCTCCGGTCCTCCTCCTCGCCCTACTCCTCGCCGTCTCGGCCGGCGCAGGCGCAGGCGCAGGCGAGGCTTCGGCGCCGGCGCTGGACCAGGTGTGCGGGAGGCTGGGCAGCTACTACGTGACGCCATCCCTCTGCATCTCCGCGCTCTGCGCCGACGCGTCGACGTCGACGTCGACGTGCCGCGCCGCGCGGGACGCGCCCGCCGTGGCGGCGCTCGCGGCCAGGCTGGCGGCCGACAACGCCACGGCGGCCAGGGACAGCATCCAGGCCGCCGTCTTCTCCCCGTCGTCCTCGTCCTCGTCCTCCGCCACCGCGGCAGCGGCGGCCGCGCGGTCGTGCCTGCAGCTGTACGCGGGCGCGGTGCCGGCGCTGCGGTGGGCGGCGCGTGCCGTGGCCGCCGGGCGGTACCGCGGCGCGCGGGAGGTGCTGCAGGCGACGCAGTACGTCGCCGCGGGATGCGAGGGCATCGCGGGCGACGCCGCCGCCGCGCTGCCTCGCGAGAACGACGGGTTCGCCGACATGGCCTTCGTCGCGCACGCCGTCGTCGCCTCCATGTCCGCCGACTGA

>SbPMEI48

ATGATGCATTCATTGTTAGTTCAACCTCCAAACGGCCACCGTTATTGCGACGAGAACGCAACTCCGGTGATGCACGCAGGCGTCGCGCTTGCAGCTGCAGGTCGCACAGCAAACGTCCACCAACAAGCAAGAGCCATGGGCAGGACCACCGCCACCACGCTCCTCGCCGTGGCCGGCGCCGCGCTCTGCTTCTTCTCATGCTGCTTCTACGGCGGCGCGGCGGCGGGGGACACGGTGGCCGAGTCGTGCGACGCGATCCGCGACTTCGTGGACGTGTCCTTCTGCGCGTCGCGGCTGGGGTCCGTGCCGGGCGCCGCCTCCGCGGACCGGCACGGCCACCTCCTGATGGCGGCGGACCTGGCGGCCGCGAGCGGGGCCTCGGCGCGCGACGCCGCGGCGGGGATGGCGCGCCGGCGCCGCGACGGCGAGGGCGAGGGCGAGGGCGACACGGACGCGCTGGAGGCGTGCGGCATCCTGTACGGGGCCGCGTCGGTGCCCGCGCTGCGGCTCATGCGCGGCTACGCGGCGGCGCGCGCCTGGGGCGCCGCGCGCGCGCTGCTGCCGCTCACGGGACAGGCCGGGATCGGGTGCGACGCCGCGCTCGAGGGCTCTGCGACGGCCAAGGCGCGGATGGCCGCCGCTAACCGCGAGTTCGACCAGCTCTCCACCATGGCCACCGCGTTCCTCAACAAGCTCACCTTAGTCACCTAG

>SbPMEI49

ATGGCCGCCTCAGCCTCAACTCCCACGCTCTCTGCTACCCTCGTCGTCTTCGTGTCCGTCGTCATCGCCGTCGGGGCGACCACGGCGCTGGACCAAGTGTGTGGCGGCCTGGGCGGCTACTACGTGACGCCGGAGCTCTGCGTGTCCGCGCTCTGCCCGGACCCGTCGCCGTCGTCGCCGTGCCGCGCCGCGCGCGACGCGCCCGCGGTGGCGGCGGTGGCGGCCAGGCTCGCGGCGGCCAACGCCACCGCGGCCAGGGACAGCGTCCAGGCCGCGCTCTCGTTCTATGCCGCCGCCGCGGGGGACGACGACGCGGCGGCGGGGAAGAAGGCGGCCTTGCGGTCGTGCCTGCAGCTCTACGGCGGCGTCGTGCAGGCGCTGCAGTGGGCGGCGGGGTCCGTGGCCGCGGGGCGGTTCCCCGGCGCGAGGGAGGTGATGCAGGCGGCGCAGTACGTGCCTGCCGGGTGCGACGGCATGGTGGGCGGCGGCGTGGCGCTGCCCTCGGAGAACGAAGGCTTCGCCACCATGGCCTTCGTCGCGCACGCCGTTCTCGCCACCCTGTCCAATGGCTACTGA

>SbPMEI50

ATGGGGCTAGCCTACTACTACCACCAACGGCTCGTCCTCCTGGCCGTCGTGGCGTTCCTATGTGCTGGCCTCTTCCCGCAGGCATTAGGGAAGGGGCATGGCGGTGCTGTCAACCCGGCGGTTGCCGGCATCTGCTCTCGCACCCCATTCCCTGAGGTTTGCAAGTCCACAGCCGGGAGGCATGCGTCCAAGTACCCGGTCATCGACAACTTGGCCGTGCTCAACATGCAGGTGGAGGCGTTCTCCAAGCGCACTGCGCAGGCGCGGCAGCACGTCGCGAAGTCGGCCCGCACTATTCCACCGGCGCAGAAGCAGGCCCTTACATTCTGCGACACAATGTACATGAACACACAGGACACCATCGGTGCGGCGCAGCGGGCCATCACGTTCAAGGACACGAGCACCGCAAAAATCATGCTGCAGCTCGCCGTTCAGGACTTCGACTCGTGTGACCGTCCGTTCACCCAGGCTGGTATCCCCAACCCCATGGGGAAGTTCGATAAGGAACTAAACCAGATGGCCAACAACTGCATGACGCTTGCAAACATGATATGA

>SbPMEI51

ATGGAGAGCTCGAGGATCATCGTCGCCTCCCTCCTCCTCCTCCTCCTGGCCTTCGCAGCCACCGCCGAGGCCCGCGTCGTCCGCGAGCTGATCGGCGAGAACGCATGCCAGCAGACATGCAACCAGGTGCACTTCAAGAAGATGTGCCAGAGCTTGACGAAGCTCCCGAAGGTGACGACGCCGCGGGAGCTCCTGCTGGCGTCGATGCGCGTCGCGGCGGAGAAGGCGAAGGAGGCCAAGAGCCGTGTGGACGAGTACGCGGCGAGGTCCCACGAGGGCCGGCCGATGGAGTCTATCCTCAGTTCCTGCAGCTCCGGGTACGACAACGTGGTGCAGACGCTGGAGGAGACGGAGAAGATCGTCGCCACGCAGGGAACCCAGGTGGACTTGAACACCAAGCTGTCGGACGCCGTCACGAGCGCCGGCGACTGCGACAACGCCTTCCAGGACTTCCCGGAGATGAAGGACCCCTTCTTGGCCATGCAGCGGAACGTCTGGCGCCTCGTGGACAACGTCCTCAACATCGCCGTCGTCGTCAAGCAGTCGGGGGACGCGCACGCCCACTAG

>SbPMEI52

ATGGGTGGTGTCACCGTGACGACGCCGCTCTGCTACCTGGCAGTGGCCGCGGCCTCGGCCCTGCTTCTGCTGAGCACCGCCGTGGCGCCCGCGCAGGTGGTGGTGACCATCGAGGAGGCGTGCCGGATGGCGACGAGCGGCGGCGCGGGGAAAGTGAGCTACGACCACTGCGTGGCGTCGCTGGCGTCGGACGCGCGGAGCCGCGACGCCGCGGATCTGCACCACCTGGCCGCGCTGGCCGCGCGGATCGCGGTGGAGCACGCGGCGGCGACGGAGGCCAAGATCGAGGACCTGGGCGAGGTGGAGGAGAGCCCCCACGCGCGCGCGCGCCTGCACCACTGCCTGGACCTCTACAACGCCGCCGCCGACGTCCTGCGCGACGCGCTCGACAACCTCCACGCCCGCGTCTACGGCAAGGCGTCCCAGCAGCTGGCCGCCGCGCTGGGCGCCGCCGAGAGCTGCGAGGACGTCTGGAAGGGCGAGGAGCACGTCCCCGTCGCCGCGCACGACAGGGAGTACGGCCGCATGGCGTTGGTCGCGCTCGGCCTCACCAGCGGCATCGCTTGA

>SbPMEI53

ATGGCAGCTCGATCGATGGCCATGGCGTTCCTGACCACCGCGCTCGCCGTGGTGCTCCTCCTCGGTGCCTGCGCGGCATCAGCACCCTGCCTCACCACCGACGCCGCCGCCGCCGCTGTCTGCCAGAACCAACAACGGCACGACGACGACGACGGCGACCTGGTGGCGACGGCGTGCGAGAGGGCCAAGGGCCACGAGGCGCACCACTTCCGCGGGCTGGGTCTGACGGCGCTGACCAAGGACTTCTGCGAGACGACGCTCCGGTCGGACAACCGGAGCGCGGCGGCGAACGACACGCGGGAGCTGGCGCTGGTGGCCATGGACCTGGCCAGCACCGCCGCCGCGAGCGCGAGCACCAAGGCGCGTAGCGCGCTCCGGTCGTCGGGGGGCAGGGGCGGCAAGGACAGGGACACGGAGTTCTCGCTCCGGTACTGCGTGATGGACTACGGCACGGTGGCGGCCGTCCTCCCGGCGTGCCGCGTGATCGTCGAGGAGTACAGCCCCGGCGACTTCCAGGCGCCGTTCGACTACCTGGAGTGCGCCGGCAGGGTGATGGACGCGGCGGGCGACTGCTGGCAGCGCGTGTCGTACGAGGATGGCGAGTTGAAGAGGGCGCTGTGGAAGGACGCCGTCGACGTCGCCAACCGGGCGAACCTCGCCCAGGCCTTGGTCGAGCAGATGGTCGATTTCCCCGACGATCATCACTGA

>SbPMEI54

ATGGCTCGCCTGCTCCTCCTCCTCGCCGCCGCCGCCGCAGCGGCGTTCCTGGCCGTGGAGGCCGCGTCGCCGGTGGCCAGCGACTTCATCCGCAAGTCTTGCCGCGCGACGCAGTACCCGTCGGTGTGCGAGCAGAGCCTGGCGTCGTACGGGGGCACCCCGCCGCCGCGGAGCCCGCGGGAGCTGGCGCGCGCCGCGCTGTCCGTGAGCGCGGACCGCGCGCGCGCCGCGTCCGCGTACGTGGGCCGCCTGTGCGGCGCCGGCACCGGCGCGAAGAAGGGGTCCGGGTCGCGGCCGGCGGCGGCGGGCCCCGTGCGCGACTGCCTGGAGAACCTGGCGGACAGCGTGGGCCACCTCCGCGACGCGGCGCAGGAGATGGGCGGCGCCGGGATGAGCCGCTCCGGGACGCCCGCGTTCAAGTGGCACCTCAGCAACGTCCAGACCTGGTGCAGCGCCGCGCTCACCGACGAGAACACCTGCCTCGACGGCCTCTCCTCCCGCGGCGTCGACGCCGGCACGCGCGCCGCCATCCGCGGCAAGGTCGTCGAGGTCGCGCAGGTCACCAGCAACGCGCTCGCGCTCGTCAACAAGGTCGGGCCTGGGTACTAG

>SbPMEI55

ATGGCTCGCCCGGGTAGTAGTGCCGCTGCTCCCCTCCTCCTGCTGCTCGCCGCCGCGGCGGCGTCGATCCTGGCAGCGGCAGCCGCGTCGCCGGCGCCGAGCGACTTCGTCCGCAAGTCGTGCCGCGCGACGCAGTACCCGTCGGTGTGCGAGCAGAGCCTGGCGTCGTACGGGGGCTCCCCGGCGCCGCGGAGCCCGCGGGAGCTGGCGCGCGCCGCGCTGTCGGTGAGCGCGGACCGCGCGCGCGCCGCGTCGGCCTACGTGGGCCGCCTGTGCGGCGGCTCCAGCAGCTCCGCCGGCCACAAGAAGGGCGCTGCTGCGAGGAAGGGCGGCGCGCCCGGGTCGGCGGCGGGACCCGTGCGCGACTGCCTGGAGAACCTGGCGGACAGCGTGGGACACCTCCGCGACGCGGCGCAGGAGATGGGCGGCGCCGGGATGTCCCGCTCCGGGACGCCCGCGTTCAAGTGGCACCTCAGCAACGTCCAGACCTGGTGCAGCGCCGCGCTCACCGACGAGAACACCTGCCTCGACGGCCTCTCCTCCCGCGGCGTCGACGCCGGCACGCGCGCCGCCATCCGCGGCAAGGTCGTCGACGTCGCGCAGGTCACCAGCAACGCCCTCGCACTCGTCAACAAGGTCGGGCCAGGGTACTAG
