# Supplementary material for: Genome-Wide Identification, Characterization and Expression Patterns of the Pectin Methylesterase Inhibitor Genes in Sorghum bicolor
Source: Genes (Basel). 2019 Sep 26;10(10):755. doi: 10.3390/genes10100755 (PMC6826626; doi:10.3390/genes10100755)
Supplement: Supplementary file 1 [file genes-10-00755-s001.zip › Supplementary Files/Supplementary File 5.docx]

>SbPMEI1

ATGCCGAGGCCACCCAAGCGAGCAGCGGCGGCGGCGGCCAAGCTCCCCCTCACGGCCACCTTCCTCTCCCTCTCCCTCTCCCTTCTCCTCCTGCCGCTCGGCTTCGTCGCCACCCACAGGGCATTCTCCGGTGGCGTCGCCGCCGCGTCCACGCCGACCGCGAAGCGTGTTCTTCTCGAGGACGACGGTCATGCCAATGCCGCCGCCGCCGAGCACGCGGCCGCCGTGGAGCGGCACTGCGCGGGGACGCTGCACCGCGACGTGTGCGCGTCGACGCTGTCCGCCATCCCGGACCTGGCGCAGAAGCCGGTGCGCGACGTGATCTCGGCGGTGGTGGCGCGCGCGGCGGCCGCGGTGCGCGCGTCGGCGTCCAACTGCTCGGCGTACCTCCGCCGGCCGGCGGGGGCGGGTGCGCTGCGGGTGCGCGACCGCCTGGCGCTGTCCGACTGCGTGGAGCTCCTGCAGCACACGCTGGCGCAGCTGGGCACGGCGGAGGCGGAGCTGTCGGCGGCGAACAGCAGCACCAGCACCAGCACCAGCACCGATGAGGAGTCCGTGGCCGGGGTGCAGACCGTGCTGTCGGCCGCCCTGACCAACCAGTACACCTGCCTGGACGGCTTCGCGGGGCCGTCGGCGTCGGAGGACGGCCGCGTCCGGCCCTACATCCAGGGCCGCATCTACCACGTGGCCCACCTTGTGTCCAACTCCCTCGCGATGCTGCGCCGCCTCCCGCAGCGCCGCCGGCGGCGGGGTAGAGAGGCGCTGGAGCTGGAAGGGTACGGGCGCGTGCGGCGCGGGTTCCCGTCGTGGGTGTCGGCGGCGGACCGGAGGCGGCTGCAGCAGCAGCAGGTGGTCCCGGACCTGGTGGTGGCCAAGGACGGGAGCGGCAACTTCACGACGGTAGGGGAGGCGGTGGCGGCGGCGCCCAACAACAGCGAGTCGCGGTTCGTGATCTACATCAAGGCGGGTGGGTACTTCGAGAACGTGGAGGTCGGGAGCGAGAAGACCAACCTGATGTTCGTCGGCGACGGCATGTGGAAGACGGTGATCAAGGCCAGCCGCAACGTCGTGGACAACTCCACCACATTCCGCTCCGCCACGCTAGGTCAGTAACTCCTCCTCCACCAGCCAACACAATGCAAGTACAGCATTTTACTCATCAGAATCAGATCACTTGCCGACAATGCAATGCAATGCAATGTTGGCAAGATGGCAAATAGATAGGAGTATGTATGTAGAAGATCATCCGTCTGTTGGGTTGGGTTGGGTCGGGACCGATCGCGCAATGCAGCGGTAGATGATCGTGATCCTTTTCTAGCTAGTGGACCACCGGCCCGGGCGGCGCTCGTGTCGGAGATGAAGACGAACGTAAGGTTAGAGTATAGTACTAGCTGTAAAGTGTGGAAGTGCTGCTGGTTGAGGCAGGCGACGACGACAGTTGAGGAGAGCACGAGCGAGCGAGGGAAGCGTGGCCACCGGTGAAAGAATGAAAGATCGATCCTGCAATAATTTGCCTTTGCTGGATCCTGCTTCTCCATTCCATTCCTGCAGTGGCTGGTAGCTGTTGCAAATTAAATCAGGTCAGGTTAAACACAAGCATCACAGTGCAACTTCGATGATTGCATCTCTAGTACGGTAGGAGTATTGCATGCGTAGCCGGCGCGTGGTGGTAGGTTGGTTGGCCGACAATTGCATATGCGTGCATGAGCCGGCTCGTGCCCGTACCCAAATCTACATGGATGAGTGTGTACATTTGATTCACGAGTGTCGTACATAGTAGTCGTCAAATTGGAGTAGTAAGGTGCAGAGCTTTGTTGCCTCTACTACGTACTCGCTCTGCTGCGACTGATTGGTCGGTTGCGGCTCGCCGATAAGAGCCTCTTTGGCAGGACTCTCGCTTCTTCTATCGTCCACTTTAGCATCACTGTTTGCCACTATAGTAAGGAGCCGTTTTGCTTTTTAAGGTGAAAAATCAACTAGAAAGAGGAGCGGGAGGAGCTACGATTTGGAGCTTCACCGGCTCCTTACTACAGTGTCACTATAGTGTTGTAAAGAGAAGGCGCCAGAGCTAGGCCTTGTTTAGATGCAAAAAAGTTTTGGGATTTTGACACTGTAGCATTTTCGTTTTTATTTGACAAACATTGTCTAGTTATGGATTAACTAGGCTTAAAAGATTCGTCTCGTGATTTACAGATAAACTGTGCAATTAGTTATTCTTTTTATCTATATTTAATACTTCATGCATGTGCCGCAAGATTCGATGTGACAGGGAATCTTGTAAAGTTTTGGATTTTTGAGTGTATCTAAACAAGGCCCTAGACAAGGAGCTGTGCCAAATAGTCCCTGTAGTAGCTTGTTAGGTTGGCGATAGCAAACTACGGCCCTGTTGGTCTGCCTCTGCGAACACAAGGTTGTTGGTCTGATGGATTTGGCTCCCATTCATTCATCCTTTTTCTAGTTGCCTTGCTCGATCCTTCAGCGTTCAAATTCAACTTTTTGGCTGGCATTTTCCTCCTTGCAGGAGCTGAGAGGAGCAATAATCATTTTTTACTAAAATATCGGAGCTCAAAAACGTGGGTTTTTCTAAAGTTCTTTCTCACAAACCTAACCATACAAAATTATTACAAAATTAGTACCAGAGCCGTATTTTGTCAAACTTTTTTTCAAAACGACTTCGGCTTTTGCCATAGAAGTTGCTCCGCGTGAAGAGTTAGTGCCGAAATCGTACAAAGGAGGACCTCCATGATATAAGCCAGCAGACACCATAAATAAACTTAATTTTTATGGTCATATTGTAACATGTCAAAACTAAAGGCCTGTTTAGATGCACCCAAAAACCAAAAATTTTATAATATTTCCCATCACATCGAATCTTGTAGCACATGCATGAAGCATTAAATATAGATAAAAAAGATAACTAATTGCACAGTTTACCTGTAAATTACGAGACGAATCTTTGAAGCCTAGTTACTCCATGATTGGACAATGTTTGTCAAATAAAAACGAAAGTGCTACAGTGTCAAAATCTAAAAAGGTTTTGTATCTAAAACAAGGCCTAAGTGTTAATTTGTGCATTTGGAGACAAATTTGACTACATACCTAGAGCCAAACTACCATGTCAGCAAATCGACGCGACGCCCATCTGATCCTAATCAACTCCCGACACCATTCCCCTTTCCACAAGCTAGAGCTAGAGACAAAAGGGGACTCATGCAGTCATGCCCATTTGGCGTGCCTTCAAACCTTGCTACAGTTCATGTAATCTATTTTGAGAGAAAAGATAAAAATAGCTTCTTGTTACAGTATACGAAGTAGTTAGAGCTAAAGCCCCTTGGAGCTCTCCACCAAGTGGGCCATGAGAGTTCTATTTGAAGCAGCATATATAGGATTTATTTGGAACTATTCTACAAGCTCCACCGTGAAGTAGCTTAAAAAAACTAAAATTTCTAGAGCATCCTCTTAGGTGTTCTCTAAACTCCAATATATTTGTTAGAGCAGAATTTTTAGAGCTATATCTGTTTAACTGAAAACACTTGGACCAAATAATTAATACTCCCTCCGTTCCAAATTATAAGTCATTCCAAGAATCTTGGAGAGTCAAAGTATTTTCAAGTTTGACCAAAAATATAAAGAGAAATATAAAGATTTATGTCATAAAATAGGTACACTATGAAAATATAACTAACAAAGAATCTAATGATACTTGGTTGGTACCAAAAATGTTATTATTTTGCTATATAAATTTGGTCAAACTTCAAAAACTTTGACTCTCCAAGATTCTTGGAATGACTTATAATTTGGGACGGAGGGAGTAGAAAGCAGTATCAACCAGGTCCACAGTCCCATACGTGTGTTTAACTGAAAAACATGGAGCGAGCGAATAATGGAACGAAGCGTGTCAACCAGGTCCATAGTCCAAGTGGGAAGAGCGCACGATGATTTATGAGGCATCACCTGATACCAAATTACCAACTCGCGAGTACGCTAGGCAGTTTAATAATGTGGCGAACGGACCGGAAGGGCTGATTGCCTGTAAAAAATATTCAGACAATGCAGCGCGGCTGCACACACACTGCACTTGAGAATTGATGAAGGAACACACTGCACTGGCGCAGGAGGGGAGGGACACGCTCTCCTTGCTTCATGGCGTTTGGAGTTATGCAGCACGGGAATGATTAGGTGAGGAGGCAAACGCCCAAATGCCCATGCATTAACGCGGCCGCGTGCGTGGGCCTGGCTCTAAGCTGCCGGCCAAAACGGAGATGGAAGCGCATGCAGTGCAAGTGATTTCCTTCTGCGATCGGGGTCTTCAAGCTCATCAACCACACCCTACAAGAGAGGAGGGCATGCATACAGGCAGCCACAACCACAAGGGTACCACACCATGGCCACCGGATCGCAGAGAGAGAGAGAGAGAGAGAGAGATGGAGACGATTTTGTGCTGCTGATATGCTGTGGTCTCCCTTCTTCTCGATCTCCCTCGTGCCTTCTCTGTGGGCCGGATTGGAGTAGGAGTACGTGGACAGGACGAGTGGTGTGTACTGGATGCCGGCCGATTGGTCGTCGTCGATCGGTCTGGTCCCTGCATGCAGCTGCATTCAGTGCCAGTGCGTGGGGTGGGGATCCATGCTAACCTCTACTCTAGTCTAGAACTGAAACACGCTTGAACAATGAACTCACAAACATGCATAGCATAGTAGTGTAGTAGGCACCGACAGTTGACAGAGTCACTCATCACTCTTGGCACTTTTTAATTGGCCGGCGAATGGAATTGCATTGATGAATCACGCTCCAAATTAAACACACAATGATCCTAAACCTGCAGCAGCTAGTTAGCGTCCAGGATCATTGCTCATTTCCTCTTGCTCATTTCTACTATGGGCGCTGCAAAGATGCCACCCGTAGCAAAAGAACAGCAGCAAGCCGACACTGCCCGCCCGCCCCATGCATGGGCCATGACCAACCGATCGACAGACCGGCCGGCCGGCCGGCGTGCCTCGTCTACCGCGCCGCGCGCCCCCGGCCGCGGCCGGTCTCCTCCTCGATCGGTCCGTGGCCTTGTCGCGGCCGCTGGACACAAGCGCCCGCTCCCTCGCTCTACTACTATAGAGCTGGTGATCGGTGTCGCCATTGCATGCCTGGCCGAGGACATCGATCCATCCATGGGTGGTGGCGACAGGGAATAGTGAAGCACCAAACGCTTGTTTTGGCCCGGCCAGCAGAGATCTCGAGGCATGAGCTAGCTAGTACCGCGGCAGACGACGATCGATATGAGAGCGAGAGGCACTACTGTCAAGCTCTGGTTGGTGGTTGGCACGCCACTTTCGCCTACTTGGTGCTGGTGCGTGTCGTGTCGGTGGAACGTAACGTACAGTACGCACGCAATGATGGTGCGCGCATCGCCAGTACTCACATGCCACTTGCATGTGGGCACTTTAATTTGCGATATAGTATATGCATGCCTACGCCCGTCGCCAGCTAGCCAGCGATACGTACGTGCTGTATGCTGTACCATACCAATACCACCGTACGTGTACAGTACGTGGTGCATGCAGGTTAAACATGCAGTCATGTCTGCTCTGACACAGATCCCCATTTGCTAATGCAAAACCGCAGCGGTGGTTGGCACGGGGTTCCTGGCGCGTGACCTGACGGTGGAGAACGCGGCGGGTCCAAGCAAGCACCAGGCCGTGGCGCTGCGCGTGAACGCCGACCTGGCCGCCTTCTACCGCTGCAGCTTCGCGGGTTACCAGGACACCCTCTACGCGCACTCCCTCCGCCAGTTCTACCGCGACTGCGACGTCTACGGCACCGTCGACTTCGTCTTCGGCGACGCCGCCGCCGTGCTGCAGGGCTGCAGCCTCTACGCGCGCCGCCCGGGCCCCGGGCAGAAGAACGTCGTCACGGCGCAGGGCCGGGAGGACCCCAACCAGAACACGGGCATCGTCGTGCAGGGCGGCAAGGTCGCCGCCGCCGCCGACCTCGTCCCCGTCCTCGGCAACGTCTCCTCCTACCTCGGCAGGCCGTGGAAGCTCTACTCGCGCACCGTGTTCGTGCAGACCAAGATGGAGGCGCTCGTCCACCCGCGAGGATGGCTCGAGTGGAACGGCACCTTCGCGCTCGACACGCTATACTACGCCGAGTACATGAACCGCGGGCCTGGAGCCGACACTTCCGCAAGGGTGGCGTGGTCGGGGTACCACGTGCTCACCAACGCCACCGACGCCGCCAACTTCACCGTGCTCGACTTCATCCAGGGAGACCTCTGGCTCAACTCCACATCCTTCCCCTACACATTGGGCTTCACCTAG

>SbPMEI2

GTGTCCGTGCATGGAGCCATGGAGCGGCCGGGCGATGCATTCCTTTCCTTGTCACCACATGCAGTATTGGCTCGCAAAGCGCTCCCGGCCGCAGGTGCGCACGAGACGGTGGCGCGGCCTTGTCGGAGGCTGAACCGCCGCGCATATATACGTGTGCCCTCACGCCAGCCGTGCATTCTAGCTAGCTACTACTAGCTATTTCGAGACGCAAGCGTCCGTCTCCGTCTCCGATCCGATCCATCGAGCATACTACGTACGGCGGCGATGGAGGCGAGGTCGGCCATTAGCTGGTACTGCGGCTCCCTCCTGGCCGTGGTCATCGCGCTGTTCCTGTCAGTGTCCCTCGGCGTGCGCGCCGCCGGCGTGGACCTGAAGGCGTCGTGCGCCGCGACGCCGCACCCGGACGTGTGCCTGCGCGCGCTCCAGGACGACCACAGCATCAAGGGGGCCTCGACCCAGCGGGACCTGGCGTCGGCGGCGATCCGCGCGGCGGCGACCGCTGGCGGCGCGGTGGGCGACTACGCGCGGGACGAGCTGAACGTGGTCAAGGACAACCTGATGTGGCAGTGCCTGAACGAGTGCGCCGAGGACATCGAGGAGGCGCTGGACCACCTGGACGACTCCGAGGGCGGCCTCGACGACGACAAGCTCCGCGACGTCAAGGAGTTCCTCGACACGGCGGAGGAGGACACCTGGTCCTGCGACGAGTCCTGCAAGCACGCCCCCAACACGCCCATCAAGACCACCCTGCTAGCCAAGAACAAGGACTTCGCCGCCGTCATGCGCGTCGCCAACGCGCTCATCAAGCGCGCCACCGCCGGTGACTCGCCGGCGCCAAGATTCATCAATTGAAGCAGTACGGTGATTCATCCATTGATTATTCATCCCCGCGCGCGCCATACACATCCACATCGTATTGAATTTGTTGTAGTATGTATATCCTTGTTGCTGGTACCTTTTTATATGTGTCTTGTCATTGTAATTTGATTAGATTGTTTGGGAATTTTTTACTACAGTATTTGTACTCAGATGTACGTAGTGCTTGATGTTCATCTACTAGCAGTTCAATGTGTAGCAAATGATTATATAGAATTATACTAATAAAAAACATCATGGTCTACATTTTCTCATTTTATTTATTGTATATACTATTAGTTTTTTCTTTGAGAAAATTGTATGTACTACTAGTGTATCACCATTTGTATGTGCTACCCTTTTTGAGATTATACTACTCTTTTTTTTTTAGAATCACTTGTATGTGGTGATATC

>SbPMEI3

CACAGATCGATCGAACACATCGTCGTTCCCCACTCACTGCTTCTTCACTCGTTCATCAACTACTGGAAATTAGATAGAGATAAAGCAGGCAGGATCTCTCGGAGATCTGGAACTAAAAAGGACGACCAGCGAATTAAGAAGGAGAGAGCTAGATCAGATCGACGACCGATCGAAGACGATGGCGTCGGTGCGCACGACGACGACGACGACGTCGTCCCTGGTAGCAGCAGTCCTGTCCCTCTGCGTCGTCGTCTCCCTCTCCCTCCGCGGCGCCGACGCCGCGCGGATCACCCCCGGCGACAGCCCGATCGTGGCCACCTGCATGACGGGCCCGTACCCGGAGCTGTGCGTGGGCGAGCTGGGCAAGCGGCTGCTGGACGTGCAGACGGTCATCGCGTCCGCGGCGCCCAACAAGGGCGCGGCCAAGATCGCCGGCGCCCCGGGGCAGGTGGACGTCAAGGCGCTGGTGTCCGTGGCGCTGGAGGCGGCCACGGAGGCCGGCACCATCTTGGTGTCCATCTTCGAGGGCAAGCTCCCGGGGTTCAACACCAGCGTGCCGGACTTCCACAAATGCATGGGCAACTGCAGCGTCACCATGAAAAGCGCCATGCAGAAGCTCCACGGCGCCAAGGCCGCGCTGCACGCGGGCGACAGGCAGGTGGCCAAGACGCTCGCGCTGCGCGCCGCCACCGACGTCTCGTCCTGCACCATCAGCTGCAGGGAGCTCAACGGCGACGTGCGCGTCATCGTCGCGCAGAGCCTCACCGAGTTCGCCAAGATGCTCCAGATCGCCATCGGGTTCATCAGCAAGATGAAGTCGGAGCCGTCGGAGCCAAAGCCACCGTCGGAGCCCAAGCCACCGTCGGAGCCAAACCCACCGCCGACGCGGACCACGCCATGA

>SbPMEI4

CAAACTCTGAAACTCGTACACCACTGCAGTACGAAAACACTCACGTAGCAGCGATATGGCACTGGCACGTACCGCCTCGTCCTCGTTGCTGCTTCTCTTGGTGCTCTCGTGCTGGTGCGGCGCCACGACGGCGCGCCCGGCGCCGACCTCTGACGCCGCGGGCACGGGCGCGGGCGCGGGCGCGGGCTTCGTCAAGTCGTGGTGCGCGGGGACGGAGTACCCAGCGCTATGCGACGCGACGCTGGCCTCGTACGCGGCGGAGGTGGGCACCAGCGCGGCGCGCCTGTCGTGGGCCGCGCTGACGGTGACGCTCGACGGCGCGCGTGACGCGACGGCCGCGATGAAGGGGATGGCGGCGGCGGGCCACCTGGCGCCCGTGGCGGCCGAGGCGGCGCGGGACTGCGTGAGCATGCTCGGGGACGCCGTGGACATGCTGCGACAGTCGGTGGAAACCATGGCGCGCCTCGGCGAGGAGGAGGAGGAGAAGCAGCAAGGGCAGCCGGGGAGCAGCAGCAGGAGGAACGTGAGGTTCCAGGTGGACAGCGTGCAGACGTGGGCGAGCGCGGCGCTGACGGACGACGACATGTGCATGGAAGGGTTCAAGGGCGAGGCCGCGGTGGTGAGGGAGGCCGTGCGGGGGCACGTCGTCGGCGTCGCGCACCTCACGGCCGACGCGCTTGCCATTGTCAACGCCATGGGCAAGTCCATGGGAGTCGATATATGCCGCGGTTCTTGCAAATCCACACCGACGCCGCCGCCTGCTACTGCCCCATAGAAACACCGAAAGCTCCACATCGAGCAAAAAGAAAAATGGTTTCAATCATCCATAGATGTCATATGTCATAAGTGTTGTAGCTTTGCTAAAAAAAGGACCGGCCCTAGTAGCTAAATAAAGTTTTCGACACATGTCTAGACAGATATCTGTATAAAAAAAATCAATCACTACTATAAAATTTTTTTCGTAACAACATTTTCATCAATATATAGTTCAGTAAAAAAACATTTATGATAATATATCACCAATTTTCACTGCCAATCTGTGTGTGCCAATGGTTGATGAAAATATTTGCACTATGTCTCTATAGAAGCCCGCCCAGCTCATATCAGCCGCTAAGAGCTTCTCCACCAGATTAATTTAAACACATCTCCTTACCCTAAAAACAACAATAAAGGGGACTGGGGTGTGAAAAAAATATGCTTCACCAGATCCCCTTTCCAACCCTATCTCTCCTTTTTTTTCTCTCAAACCCATAAACCCCTCAAAACCATCCCTCTACAAAAAGAATCGGCTAAAGAAAATTAAGTACACAAATGATTTGACTTTGTGTAATCATATTTTTTTGATATTTTGATAGAAAATAATTTCATTAGAATTATCGATAACAAGGTGTTGACATCACTCTAGAACTTATAAGAGCATACATATTGTAAGTGGTATAATTCATTTGATTTATTACTTGTAATTGCA

>SbPMEI5

GCGGTTCGTACGTGCATGCTGCGGCCTCGATCGATCGGCGAATCAAAGGCGAGCAGCCGAGCACCCTCCACCTCTATGCCATCCCACAGAAAAGGGCTGGAGAGGCCAGCCGCCGGCGGCGTCGCCTACTACGTATCCTGGCAATCCTGGCCAGAGACGACGTGCCGGTTGAGCCAGCCATACGTACAGGCCGCGCCCTCATCGTCCTGCTGCTGAGTGCTGACTGCCCCTATAAAACTCGACGCTGGCCGGTTCTACACACCACTTGAGAGTCAGAGAGCCGGAGGCAGCAGTAAGAAAACACGTAGAAACCGTACGCGCGGCGCCATGGCACGCAGCAGGGCCTTGCTGTTGTTTCTTCTCGCGCTCTCGTGCTGCTGGTCGTGGTGGTGCGGCGCCGTCGTCGTCACGGCGCGGCCGACCCCGAGCACCACCACCACAGCCGGCGGCGGCGGCGGCGGCGGCTTCATCGCGTCGTGGTGCGCGGGGACGGACTACCCGGCCCTGTGCAACGCGACGCTGGCCCCGTACGCGGCGGAGGTCGGGGCCAGCCCGGCGCGCCTGTCGCTGGCCGCGCTGACGGTCACGCTGGGCGGCGCGCGGAAGGCGACGGCCGCGATGAAGGCGATGGCGGCGGGCGCGAGCAGGAGCAGCCCCGTGGCGGCCGAGGCGGCGGAGGACTGCGTGGGCATGCTCGAGGACGCCGTGGGCCTGCTGCGGCAGTCGGTGGAGGCCATGGAGCGCATCGGGAAGGAGGAGGAGGAGCCGAGCGGCAGCAGCGGGCAGCAGGGCGGGAGCGGCAGCAGCAGGAGCGTCAGGTTCCAGGTGAACAGCGTGCAGACGTGGGCGAGCGCCGCGATGACGAACGACGACATGTGCGTGGAGGGTGGCCAGGCCGCCGTCGTCAGGGAGGCCGTGCGGGGGAACGTCGCCGGCGCCATGCACCTCACGGCCAACGCGCTCGCCATCGTCAACGCCATGGCCAAGCAAATATCGTAGGGTACGGTGTTATATGCACGTGTATAGCATGCATGCATGTGGATGCCTTGTGTATATAACTATATATATATGTACGTATACGCCTCTGTGTGTGTGTTGTTGCTGTCGTCGTCGTCTGAGATTAGAGCGGTAGTCGAAATGATCACATCTGATCCTAATTTGTTTAGAGCCGCAAATGGAAGAATCACGGATTGATTGTCGTGTGCTGCACCCGTGTCCGGACAGTGCAATGGAAGAATCATGATTTTAATTTGTTGTTATGCGTTGTTCTTCACTTTTCTAAGGCCTTGTTTAGTTCGCAAAAAAATTTGGATTTTTGCTACTGTAGCATTTTTATTTTTATTTGACAAATATTGTCTAATTATAGACTAACTAGCCTCAAAAGATTTGTCTCGTAAATTATAGGTAAACTGTGCAATTAGTTTTTTTATCTATATTTAATGCTATATGCATGTGCCGCAAAGTTTGATGTGACGGGGAAAGATTTGTCTCGTAAATTACAGATAAACTGTGCAATTAGTTATCTTTTTTATCTATATTTAATGCTCTATACATGTACCGCAAAATTTGATGTGATGAAGAATCTTAAATTTTTTGGGAACAAGGCCTAAGTACTGGTGCGACGAGCAGAAGACCGTGCCGAATCAGATTGGATACTAAAGTTGAGTCTGGAGTCCTTTTGATATTCGCAACTGGTACTCAATCAGGAAATGTGTTTGTACCATTCAAATTAACAGGAACACACTCTGCTGTCAGTAGATTGATGATGAAAGACAAGTAGTGGCGCTTGAGCTTATCAGCCGAATCTACCAGCTATTCAGCAGTGTTTTTCTCTCACAACAAATCAGCCAACAACAAATCAGCCAACAATTTCCTGCCATGGCTTATCAGCCAAACGAATAAAACGGATCTATTTTACTGGTTCGGGTACTATAACTACTGTGCAATGACTGCTCCCTCCG

>SbPMEI6

ACGTCCCGCTTTTGTAGCCAGCTAACTGGTGGACCGTTAAATTCATCAGACCATTCCGGCCGATAATAAATCGACGTGTGGTGGGTCGACGACGTACTTCTTTGCCCCCATTCAAGTTAGAACAATGGCGTCAGCAGCAGCCGCCTTCCTCCTGATCATCGCTGGAGCCGCATGGCCTTCGTTAATCGACGCCGCTCCGTCTCCGTCGTCGTCGTCGTTCGTCCCGCCGCCGCCATGCGCACCACCGCGATCGGCGGTGGAGTTTCTCCGCGCGCGCTGTGCCAGCACGCTGTACGGCGTGGCCTGCTACGAGTCCCTCCTCCCGTACGCGTGCATATTCCGTACCAGCCACGTCAAGCTCGCCCGCGCCGCCGGCGACGTCAACGCCGCCTGGATCAGCTCGATCTCCAAGCGCGTCAAGGAGCTCGTCGCCCGCGGCGCTGCTGGTGGCACCGCCGTGGCCGAGTCGGCTGCGCTCCGCGACTGCGCCAGCACAGTGTCATCGGCCGCCGGCTTGGCCAAGCAGGCGGCGGCCGAGCTGGCCAAGCTCGACGCCGCCGGAGGCGCCGTCGGGAGCAGCAATGTCCGGTGGGCGATTTCCAACGCCCAAACGTGGCTCAGCGCGTCCATGACGAACGAGGCGACATGCGCCGACGGGGTCGCGGCTACAGGCGCTGCAGCGTCGTCGCCGGTGGCCAGGGAGGTAGTGATGGCAGTTGTGAGAGCCAGGGAGCTCACGAGCATCGCTCTCGCACTCGTTTATGGGATACCAGTTCCTCCGTGATCGCCAGAGAATCCACAAACTGCTTGTAGTTCCGTTAATGTTTCCTCTCTAGACGCTCGAAGATAGATTTCCAATTATATTTGTGGAATGTTTATTACATGCAAGGTTGCAATAAAATAGGAACACTTCGTAGCGATGAGGCAAAGCCATATGATTAAATGTCACCGGGTCAGTCTAAACATACTAGGACTAGCTATTTTACACCCTAGGTGTAGAAGCTACACCGAACTGTTATACTGAGCAAAATTCAGTATCGTTCAGTATTTCTATTTTAGTATTGTTCAGTATTTTGTGATCTAGGGTGTTAGACAAGATGATACTGAACACTCTACGTACTGCTCTCTACAGTACTGCTCAGTATTTTTTCTACCTAGGGTGTAGAATAGCTTGTATATATGCGTCATCGGTTTACCACAGTCCACATGTACAAACATTCCAAGGATATTTTATATCTAGGGGAGAATGAGTATTAATTTACTA

>SbPMEI7

ATGCCCATCGTTCTCGCGTTCCTCCTCGTCGTGGCTGCAGCATGCCAGCCGGCAGCCTCCGCATCGTCGTCGGCGCCGGCGAGTAGTCTTGCTCTACCAGCACCAGCGCCAGCGCCGGTGCCGGACGCCAGCGGCCACGGAAGCCGCAGAGCGGCGCAGGCGGAGGCGGAGGCGGCGGAGTTCGTGCGCGCCAGCTGCGGGCGCACGCTGTACCCGCGGCTCTGCTACGCGGGGCTGGCCCCGTACGCGGCCTCCGTCCGCTCCAGCCACGCGCGCCTCGCGCTCGCCTCCGCCAACCTCACGCTGGCCGCCCTCGACGCGCTGGCCGCGCGCATACCGTCTCCCTCTCCCGGCTCCGGCTCCGGCTCCGGCGCGCTCAGCGACTGCGCGGACGCCGTGGCGTCGGCGGAGGACCAGGCGGCGCGCGCGGCCGAGCGGCTGGGCGGCGTCGAGCAGGCCGTCGGCGGGCGGCCCGAGCTGCTCTGGCGCGTGGACGACGCGCTCACGTGGCTCAGCGCCGCCATGACCTACGAGGACTCGTGCGCCGACAGCCTCGGCCCCCGCAAGTCCGCGCCGGCTCCGGTGAGGGCCGAGCTCCGCGCAAGGGTGCGCCGCGCCAAGCAGTTTACCAGCATCTCTCTCGCGCTCGTCAACATCCTTGTCAGTAACCCGAGAAGTTAGGCAAACCATGGTCACTTTTTTTATTTTGATAACTTCGTATTCGCATACTACAGTGTAAATTGACAGTCTTGGCTCTGTGTGTTATATTAGTACAATTTACATCTTCTACATGTACGTAATAACCATAATATATAACACTTTTTTTACGAAACTATGTAA

>SbPMEI8

GTCTCTGTACCGTTCCTTCCATTGCCCTATCGATCAAAGCCATCAGCGAAACAGTAGCTAGCGATGAACATTGGCGTTATGGCGGCAGCAACAACGACGACGATTTCCTCCACGCTCGCCGTGGTGCTCATCTGTGCCGCGTCCTTGGTGGCAACCGGCGCCGGCGCCGGCGCCGGTGGCCACCATCTCGAGCCCAACGACCTGGTGGCCAAAACGTGCGCCACTGTGACTAGACGCCACTACAGAGGGCCCGGGCTCACCAGACAGTTCTGCGAGTCGGCGCTGCGGTCAGACAAGCGCAGCGCGGCGGCGAGGGACGCACGCGACCTGGCGCTCGTGGCCATGGACCTCGTCCAGAGCGGCGCCGCGGAGGCTGGCGCCAAGGTTGGCGGCGCGCTGCGCTCCGGCGGCGCCGCGGCGAGGTGGAGCAAGTACACGACGCTCCGCCTCCAGTACTGCCGGCAGGACCACGACGACGTGGCGAGCACCGCCCCGAACTGCCGCGCTTTGGTCCGGGAGTACAATCCGCGTGCCGGTGGTGGCCGCCATGGCAGCGGCAACCTGACTCCCTTTGAGTACCTGGAGTGCGCAGGCAGGCTGGTGCACGCGGCAGATGACTGCTGGGTTCACATGCTGGACCAAGATGGTGCGGCGAAGAAGGCGGTGTGGAAGGAGATCGTCGAGGTCGCCAGTCGGGCTAACCTCGCCAAAGCTATGGTCGAGCAAATGGTTGGCGTTGTTGACGACCATGATCATTAGAGCCTTCTACTAATCTATTTGTGGAGCTATAGCTAAAACCATGTAATTTGATAACTTGCTCCTATATATTAGAGTTATATTATAATGACTAAGAGCATCTTCAAGACTTCAACGGTTTAGCAAAATTCGCTTCCCAAATCTTGTTATTTGTCAACTCCCAAAATAATACAGGGAAATATGTCAAAGTTTTAAAAATATGAGAAATTAGAATGTCAACTGTTGAAGAAAATTATTTCTTATTTTGCTAAAAAAAATATGAATAGGAAGTTATTTGCCAAACTATTGGATTGACTTGAAAATATTTAATAATAAGAAAAGGGACAAATCTTTAATAGATTCTCAAAAGGAAAATATCTGGCTATCTACACGCATGTAGACTCTAATCATCATTTATAAAATTAAAATTCAATCTAATTAGCATCTATATTATCCATAACCAAGGTTATGCCCCGAGGCGGCACTGCGCCCTCCGGCTTGCGCTCGGCCGACCGACGCTCGCCGAGTCGCCGGTGAGGTCACACTCACACGCTCGCAGCCCCGTGCACCACTACGCACAGCGCACGCCAACCTGGCTTCGGGACTCGTGGACTAGCCCCGTACGCTGCCTGGCGTGGGAGAAATTGGGCCAACATGCCACTAGTCATATTGGGCCGATCGAGTGGGGAAGAAAAGCTACAAGTCACGTCTGTTGAATTCTTACAGGATACTCGTTTTGGCTTTATTTTCTTTTTTCTCCTACACATAAAAAAATGTATACACATTATACATATATTTTTTCTAGCAAAAGAATTAGATATCTAACTAAATATCCTTGAGAATAAGTGGGCCTGTCCCTTGAGTGATTAAGTGCTTGAGTGGTTGAGTCTAGTATATTGTCAATGTTTGAGGCCTTGTTTAGTTCGTGAGTGAAAAGTTTTTTGATGTCATATCGGATGTTTCATAGGATGTTGGAAGGGGTGTTCGGAAATAAATTAAAAAAAATAATTACATAACTTGCGTAGAAACCGTGAGATGAATTTATTAAGCATAATTAATCTGTCATTAGCACATGTATGTTACTATAGCAATTATGGATAATCATGAATTAATTAGGCTTAGAAGACTTGTCTCACAGTTTCCATGCAAATTGTGTGATTAGTTTTTTTATCTATACTTAATGCTCAATGCATGTTTTTAAACATTAGATGGGACAGGATAAAAAGTGTTTACTTGAATGGCCCGGAGGTTGCCGGTGCAAAGCTCATTGGCTGCACCGACCTGAGCAACCTAAGTAGTGTGCTAAGAAGGGGAGTAGGAAGAAGAGACGGAAGAGTGAGGAGGCATTAAGATTTGGCTGGGCTTTTTATATGAGTGCATTTTCAAACGGCTACATCACAACCATCCAACCCAGATCAACACGTTTGCCCAGGCTTTAGGTTTTTTCCCCCTAGTGGGAGAACTGCCTGGGCCGGCTCGGCCATAACCAAGCCACGGCCTAG

>SbPMEI9

CATCGATCGCTTGCAAAGAGCACACGAAGAAGGTTAGATATATAGCATGTATATAAAGCCCGTGCAGGTGGCAGCCTTCCCCTCACCACCGTCGCCAACGCCAAGAACCACAACACAGACCACACAGGGAGGACGACGAAGGCGACCGCGCCGGCGCGACGCGCCATGGCCATGGCTCGCTCCGTGGCGCACCTCTTCTTTCTCCTCCTCCTCGTCTCCACCGCGCCCGCCGTGCGTACCATCCCCGACGCCGCCGCCGCCGCCGCCGGCGGAAACAACAATAACATCCAGGAGGCGTGCAGCAGGACGCTGTTCCCCAAGGTGTGCGTGCAGGCGCTCAAGGACAACCCGGAGTGCCAGGGCGGCGGCCCCGCCGTCACGCCGCGCCGCCTCGCGGAGCTGCTGGTGTACGTCTCCGCCGAGGTGGGCATGACGGTGGCGGCGTTCGCGCACCACGAGCTCAACGGCATCAAGGACGACGTGCTGTACAAGTGCCTCGACACCTGCTCCGAGGACATCGAGGAGGCCGTGGCGCACCTCAGCGCGCTCTCCCGCGACTTCTCCGACGCCAAGTTCCTCGAGGTCAAGTCCTGGCTGTCCTCCACGCTCGGGGGCACCTCCACCTGCGAGGACGCCTGCAAGGACGCCCCCGTCAGCGACATCAAGAACGCCTGCGTCACCAAGAGCTTCGAGTTCGAGAAGCTCCTGCGCGTCACGCTCGACCTCATCACCGAGGCTTCCGGCTCCATGTCCGCCGCTGAGGTCGCCCTGCCGCCCTCCGGCGGCGGCGCCAGCGCGCCGTCGTCGTACGACGCGGCGGCGCCGTCGTCCGGAGGTTACGGCCCGTCCGCTGGTGGCGCCCCCGCCAGCGGATCACCGGCACCGGCCCCGGGGAAGAGTACTGCTTCCGACGACGCGGACGCGACTGCATGATCGAACGACGCACACGCACGCCTTATGTGACAAGGATTTTGTTCTTTCCATCCCACAAATTTCCATAAGTATGCCAGTTTAGAATTAGAGTTATTTGGAGGGTCGAGCTGCATATAATTGTTAATTGATGCAAATATAATGAGGCCAGAAAATGGCACATAGGCCTTAGCTGAGTGTGTTTTGTTATTATTATTACTACTTTTTGATGGGATGGGTTGAAGGATTCGATGTAGCATTGATGTTTTCTTTTGCGAAAAATGTAACATTCATGTTAGCTATGTTACTTTGACATGTTGCAGCAGTAAAGCCATCATCTATTTTTTTTTTCT

>SbPMEI10

GACCAGTCACACCACCACCACCACCACCTTTACCACCGGGAGAGAAACGCGAGCAACCAGCGATCGCCATGGCTCGCTCTGTCTCCGTCGTCGTCGTCCTCCTCCTCTCCGTCCTCGTCTCCGCCGCGTCCGCCGCACGGACCGTGGGCGACACCGTGCAGGACGCGTGCAGCAAGACGCAGTTCCCCAAGATCTGCGTCGACAGCCTCGCGGCGAAGCCGGAGAGCCAGAAGGCGACCCCGCGGAAGCTGGCGGAGCTGTTCGTGAACATCGCCGCCGAGAAAGGGTCCGGGATGGCCACGTTCGTGCACGGCAAGTACAACAACGACGCCAAGGACAGCGCCCTGTTCAAGTGCTACGACAGCTGCTCCGACGACGTGGAGGAGGCGGTGGCGCACCTCAACGGGCTCGTCCGGGAGCCCACCGACGCCAAGTTCCTGGAGCTCAAGTCGTGGCTCTCCTCCACGCTCGGCGGCACCTCCACCTGCGAGGACGCCTGCAAGGACGCGCCCAAGAGCGGCGACAAGGACGCCGTCGTCAACTTCAGCCTCGACTTCGAGAAGCTGCAGCGCGTCACGCTGGACCTCATCACCGAGGCGTCCGGCTCCATGTCCGCCGGCATCGCACTGCCGCCGTCCGACGCCGGGGCGCCCAGCTCCTACGACGCGGCCGCGCCGTCGTCGTCCGGAGGAGGCTCAGCGGACGCCCCCGCCGGCGCTGATGCCGGCGCCGGCGCCGGCTCCGAGGGCCCTGCTGCCGCCAGCGGCCCGTCGTCTGGTGGTGACGCGCCGGCGGACGGCGGCAGTGCCGCCAGCGGGCCGGCCGCTGCTGATGCTCCGGCGGCGGCGGGGGCGGCGTCATCATCCGACGGGCCCTCTGGCGCACCAGCGCCATCGTCGTCTGATTCGGCCTCTGGCGCACCAGGACCGTCGTCTGATGGTGGGTCGTCCAGCGCTCCGGCGCCAGCGGGCGGCGACGACGACGACGATGCCGATTCCGACGACGGGTCAGCTTGAGCGTACGTGAGCGATGATGACGCGCGGGTTTGCTTGGATCATTAGGTGCCTACTCCAAGACGATCGACGATCCCCTTCTTCCCATTGCATTTTTTTTTGCCGTCCGGCCGTTTTCCTCTTGACAATGTATAATGATAATAACAGTGGATGATCGATCCTACGTACTTACGTACGTATTTATAGCTAGATGAAGGTGTCTGTGTAGTGACCCATCCATAGACGATGATGCTGACAGATCGCACGCAGTCTCCTTAGTCGGCTGATCCATTATAACATTAATGATATCAAGTCTTTCATGTTTTTTTATTACATTTATTTATGGCAATCAAACTAATGATCGATGCCCTGTCTTCTATCTTGTGATATGTATATATGTG

>SbPMEI11

ATGGCCACCATTCCGCTCCTCGTGCTCCTGATGGCGACCATCTTCTCGGCCGCACCGCCGGTCGCCGGCGAGCCTCCCAACGTGGTGCCCTTCGCGTGCAAGGCGGCCACCGCCGCCGGCGGCGGCACATTCGACGAGGCCTTCTGTCTGTCGACGCTCGAGGGCAGCAAAAGCAGCGTGGGCGCCGCGGACTACGCGGACCTGGCCGTCGTCGCGGTGGACCTCGCCACGGCGAACGTGACGGCCACCGAGGCCAAGATCGACGCCCTGCTTGCCAGCAATAACATCAGTGGCGCGGCAGTCGTCCAGGGCCTGCAGTCGTGCCGGGCGCTGTACGGCGCGGTGGTGCGGCAGTACCAGCCGGAGTGCCGCGCCGCCGTGAAGGGCGGCAGGTACGGCGACGGGAAGAAGTGTCTGGGCAGGACGGCGCAGGCGGTTGCGGCGTGCGAGCGGTGGTTCCAGCAGCGGAAGGTGGCGTCGCCGGTGGCCGCGGAGGACGTCGTCCTCGCCATGCTCGCCAACCTCGCCATCGCGCTGGCCTCCATCGCCAATTGA

>SbPMEI12

ATGGACTCCACTTTCACCGATCGTAGCACCACCGCCATTCCGCTCCTCGTGCTCCTGATGACGACCATCTTCTTGGACGCACCACCGGTCGCCGGCGAGCCACCCAGCGAAGTGCCATTTGCGTGCAAGGGTGCCGCCGCCGCCAGCGGCGGCACATTCACCGAGGCCTTCTGTCTGTCGACGCTTCATGGCAGCAAAAGCACCGTCGGCGCCGCGGACTACGCGGACCTGGCTCTCGTCGCGGTGGACCTCGCCACGGCCAACGCCACGGCCACCGAGGCCATGATCGACGCCCTTCTTGCTGCCGGCACTAATGGCGCCACGCCCGAGGGGTTGGGGCTGCAGTCGTGCCGGGCGCTGTACGGCGCGGTGGTGCGGCAGTACCAGCCGGAGTGCCGCGCCGCCGTGAAGGACGGCAGGTACGGCGACGGAAAGGCGTGCCTGGGCAGGACGGCGCAGGAGGCCGCGGCGTGCGAGCGGTGGTTCCAGCAGCGGAAGGTGGCGTCGCCGGTGGCACGGGAGGATGACGCCCTCGCCAAGCTCGCCAGCCTCGCCATCGCCCTGGCTTCCATCGCCTGATTTTTCCAGTGGCTTTAGTTTGCACTGTGATCTTATTTGATAAGCTGAAACTAACAGCCAGTTTAACCGGCGGTATTATCTAGGTGCCTAAACTTCTAAAGCTATATTTGTGTGTGCGTAAATTTGGCAGACTGTGTCATCTATATCTCTGAACTTTTAAGTAATCACCACAGGTC

>SbPMEI13

ATGGCGGCTTCAACGAGCAGAACATCACCCGGAAACCACAAGGTTCTCCTCGCCGCTCTGCTCTGCATCGCCGCCTTCTTCCCCGGATCATCCGCCAGCACGCCCTTGGTCGCCCAGACCTGCGGCAGGACGTCCAACCAGCGCCTCTGCGTCTCTCTCCTCGAGTCCAGCAACCGGAGCCGCAGCGCGACGACGGTCCGGGATCTCGCCATCATCGCGGTGAGGGGCGCCAGGAGGTCGGTGCTCCGCGCTAGGCTGCGCGCGTGGGACCTCAGCCACGGGGCGCGGCGAGAGACGACGACGCCGGCGGCGGGCCGGCTGGTGGCGAGGTGCGCGGCGCTGTACAGGGACTGCCTCCACGCCGCGGCGCACGCGCTGGCCAGGGTGACCCACATGCCGGCGTACGACGACGGCGGCCGCGTGGCCGCCGACGACGCCGTGGCCGCCCTGCGCGTGTTCCCGGAGAAGTGCCAGCGCCTCTTCGACGCGCAGGAGATCGTGTCGCCGCTGGAGCAGGTGAACCGGGACACGGAGGATAAGCTGCGCGTCGCGTCGGAGATCGTTCACTTGCTGCGCCGCTCTAGACTAGAGCCCCCTTCTCCCTCGCGGCATGGTGATGTCGAACGCACAAATGTCTAG

>SbPMEI14

CAGACCTTAACCATTAGCTAGCCGTGAGTTCTGAGTTGTGACTTGTGAGGAACAATGGCAGCCACTGGGCTATACGGCATGGTCACCTTTCTGCTCCTACTCTCCCTCCCCGCCGCTCTAGCCGATCCCAGCTTCTTCAACCGGACATGCTATCTCACGAAAAACCCCGGTGCATGTCACTCGGTGCTCGGCCACTACTTCTGGAGCCTAAACGCCACCACACTGCCCCAGCTTACTAGCACCGGGCTCGACGTCGCCGTGTTCAAGGCCAGAGACATCATGGGCGCTATGAGCGAGCTCTTTGGAGAGGGGAGGTACGCGGGCACGCACGAAGGGGACGCGCTCGCGCAGTGCGCACGGCTGTACGACAAGACCATGAGCTACGACCTCGACCAGGGGGACATGCGGCTCGTCGACGGGAAGTACAGCGACGCGCTGCGGTTCGTGACCCACGCAAGGAGCGCCGGAGATGCCTGCGAGAAGGCGTTCGCCGACCGCGGCTCGCGGTCCGTGGTGAGCGACTTGAACTAGATGATGAAGCTGTATTGTGATGTGGTCGTGGATCTTATCGAACTCCTGATCAATAACGCACGCAGTAGCAAGTAATCCTATACGTATGTAAAGGGGGATATTTCTTCTTAATAACATTGCTAATCTGTCCCCACTCCTCCTCTGGTCAATCCCCCACCCGTCCATGTCCATGTTTGATCGGCCTTCGTGAACTTAGAGGTGAGAGATATGTTATCGTTAGAAC

>SbPMEI15

ATGGCGACGAGCAGGGCGGCATCACTAGCAAGCCTCAAGAGCGTCTCGGCGGTGGCGGCGCTACTCTGCGCCGCCGCCGTCGCGTCCTCCTTCCTCCCGCTCTCGTCCGCCGGCGTGTCCCTCCTCTCGCGGACCTGCAGCAAGACGGCGCACGAGCGGCTCTGCATCTCCACCCTCGCGCCGGACGGACGGAGCGACGCCGCCCAGTCGGTGCAGGAGCTCGCCGCCATCGCGCTGAAGGTGGCCAGGAACTCCACGCGGGACGCCGTGTGGCGCACCACGGTCCTGGCCGGCGCCAGGGTCCGGACGCCGCTGGAGCGCGACCGCCTGGCGCAGTGCCGTGCTCTGTACAACGAGTGCCTCCGGGAGACGACGAGGACGATAGGCCTGGTGACCGCGGCGAGCTACGACGCCGCGGCGCGCGCCTCCAGCACCCTCCACTGGTACCCGGAGAAGTGCCAGAGCCTCTTGTACAAGCAGGGGGTCGAGTCGGCCATGGAGCAAACGAACAAGCAGGTGGAGGAGCAGCTGATCGCCTCAACGGATCTCGTTCACTTGCTACTTGTTAGGCGGCACGGAGCAGGCAAATTAAACCTAGAGTAACTTATATTAAATACGGTGTGGGTGATGGTGCACCGTGCACGCATTGCTTACCACACACACAAATACATGTGGTTTTATCCAAACATATATATCTATTA

>SbPMEI16

AGCCCCTTTCGCACCCGCAACCCTGCAACTAATAAAAACCATCGGCTAGCGATGGCCGGAGCCCGCGTGCTGCTCATCGTCCTCGCGGCTGCCGTCGCGCTCCTGGCCGCACGCCCGGTGGCGGCCACCGGGGGAGTTGCCGGTGTGGAAGAAGTCTGCAGGAGCACACCTTTCCCGGATCTGTGCACCAGGACGGCGGGGAAGCACGCCGAGAAGTACAAGGTCGTGGACGCGGTGACGGTGCTGGAGATGCAGGTGGACGCGTTCAAGAAGCGCGTCAAGGCGGCGCGGAGGGTCGCGAAGCAGGAGGTGAAGACGGCGGCGCCGACGCCCTTGGTGCGGAGGGCGCTGAACCTCTGCAAGAGCTACTACCTGGACGCCGGGGACAACCTCGGCGCCTGCAAGCGCGCCATCGGCTTCAGGGACGCCGTCACCATCCGCGCCACCATGAGCATGGTGGCACAGGACATGCAGAATTGCGACGAGGAGTTCAGGAAGGCCGGCTCCACCAACCCCATGGAGGACCACAACAGGTCGCTCATCGAGATGTCCGAGATCTGCCGCACGCTCTCCAACATGGTCCCTTACGAACATACCCATTGATTTTTTTTTTCCACCCACAATGTTCAGTAACGTCGTCGTCGTCGTCGTCGTCGTCTAAGTGCGGCCTAACATGCAATTTGATGGCCGCGTGCGTGTGTGTGTGTCAGTCGATGATAGAGCTGGACGCATGCCACAGTTCATGCGGAGCTAGCGCGTCCTCCTTTTTTCTGTGTTGCCTCATGCGGCTCGTTGAGAGGCACGCGTGTAGGGCAGCCTATATGTAATCCTATTATAACCTGCTAATTTGTGCTTCTTGTATAGCCAGTCTCATCATACAACTAAAAATGTTGCTCAGTTCTTGGCAGCACAATACAGAGTAAAACTGGTCTTCTTCCTCGTTTTCGCCTTAGTTGAGTGGCTGTTTTTACAAAGATTT

>SbPMEI17

ATGGCAGGAGCCCGCACCCTGCTCCTCCTCCTCGTCGCGGCGCTCTGCTTAGCCACCGTGAACGCGGCGACGCTGGCGGAGATCTGCAAGGGGACGGCGTTCCCGGACATCTGCACCAGCACGGTGGGGCCGGAGGCGGCGAGCAACCCGGTGCTGGACCCGATGGCGGTGCTGCGGATGCAGGTGGACGCCTTCAACCAGCGCACGGAGGCGGCGAGGGCGCACGTCAAGGAGGCGGCCATGACGGCGTCCCCGAAGGCGCGGACGGTGCTGGACCTGTGCAACAACCTGTACCTGGACGTGGAGGACAACCTGGGAGCCTGCCGCCGCGCCATCGGCTTCAAGGACGCCGTCACCATCCGCGCCACCATGGGCATGGCGGCGCAGGACATGCAGAACTGCGACGAGCAGTTCAGGCAGATCGGCGAGCCCAACCCCATGGAGCAGTTCGACGCGTCGCTCGTCGAGATGTCCGAGAACTGCCGCTCGCTCTCCAACATGATCTGA

>SbPMEI18

CAAATTACCAAAGGACAAGAGAGAGCGGGCCAAATATGGCTTCGGAAATGTCCTACGCTGCCGTCGGCGTCGTCATCCTCTCCGTCCTCGTCGTCGTCGCCGCGGCGTCCGCAGACCCCACGGCGGCACCTACGGCCGCGCCGTCGTCGAGCAAGCTCTCGCTCGAAGAGGCGTGCAAGCAGACCGCGGGGCACCACGACCTGTGCGTGGCGACGCTGTCCGCGGACCCATCGTCCAAGACGGCCGACACGGCGGGGCTGGCCCGGTTGGCCATCCAGGCGGCGCAGCGGAACGCGTCGGAGACGGCGGCCTACCTCTCCAGCTTCTACGACGACGACAGCCTCGAGAACAAGACGGCGCAGCTGCAGCAGTGCCTCGAGGACTGCGGCGAGAGGTCAGTGCGTCGTGTCTGTGTGTGGCTCTGTGCCTGCCTTCCTCGACGACCTCGAGTGCTATATGTGCCTGCGTAGCTGACAAGCACGCCCGGTCGAGTGCATGCAGGTACGAGTCGGCGGTGGAGCAGCTGTCGGACGCGACGTCGGCGGTGGACACGGGGGCGTACAGCGAGTGTGAGGCGCTGGTGGTGGCGAGCCAGGCGGAGGTGAAGCTGTGCCAGCGGGGGTGCCAGGGCGTGCCGGACCACCGCAACGTCCTCACGGCGCGCAACCGCGACGTCGACCAGCTCTGCAGCATCGCGCTCACCATCACCAAGCTCGTCGGCGGACCGCCATCGTGATACAGGACGTGATCCACGCAATTATATATATTGATAGTTTACTCAGCTCTCGATCATGGCAGCTGCTAGTTTGTTGTATAACCATGTCTCGATCGTTGTAAGCAGATCGGTAGTCGGTGGTTAAACAGATTAAATTACCTTAAAGTTTTCGATCCATATATTCTGTCAGGAGAATTCTTGTACCATAGTGATGACATAGTGGAATGCATAATATAGACGATAGACCCATACTATAAGACATCCT

>SbPMEI19

ATGGCGGCGAGGCTTGTCTCCGTCTCCGTTCCCCTCCTCCTGCTCGTCGTTGTCCTAGCCGGCTCCCGCGCGGCGCTGGCCTCCGAGACCGTGGACCAGACCTGCGCGGCTGCGCCAAGGCCACGTCGGGCGCGCAGCGCAAGGAGCACCTGGCGTCCTTCTGCGTGTCTTCGCTCCAGGCGGCGCTCGGCAGCGAGGGCGCCGACGCGCGCGGCCTGGCCGCCATCGCCACCAACCTGACGCTGGCCAACTACACGGCCGCCGTGGCCACCATCAAGGCGCTGGAGCGGCGGGGAGGCTGGCCCGAGCGGTCGCGGCGCGCGCTGGCCACGTCACGTGCCGCCGGCGGTACAATCGAGGCGCTCAACGTCGTGCACAGCGCCGTCCACGCGCTCGCCACGGGGAAGCTCCGGGGCTACGTGTTCGACATGGAGGTCGTCCGGAAGGCGGCGTCCGACTGCGAGGACGCGTTCGGCGGCGCCGGCGGGAACTGCAAGTTGCCGCTGCGGAAGGTGGACGACGACGCTGACAACCTCACCGTGGTGGCCATGCTCATCGTCAGATCCCTCGGCAATTAATTAAGGGCGGAGGCCGAGGCCATTGGTGTTGCGTTAAATTACCTTGCAATTGTAATCAAAATGATCTCTTGAGGTTCTTGTGTGATGTGCCTATTTAATGCGAATGAAACTCAGATGAAACTCCCACTGAGACTGGCCTAA

>SbPMEI20

ACTTATGGCCAATTTTCGCCTGCAGTCAACTCCCGTTCGACCTCTTTAGCAGCAGCACGAATTTTTGGGACACGAATGACGTCCATTTATAGCCATTTCACGCCCTTTGCCTCTCTTGTTTACCTCACGGTCATTCCAAATCCGAGAGGACCGCCGGCCGTACCACGCAATGTTCCGATACCTCGCTCCTCTGATTCTCGTCGCCGCCTTGGCGGCGTCTACGACGAACAAGTCCGTTGATGCCCGCGTGGTCCACCCCATCGTCGGCCCCGACACCCAGGCTGACCTCGAGGCCGCCGGCCCATCTCCCGCCGACGACGGCGACGACGGCCGCCGCCTGATCGGCACCGCCGGCGGCGGCCACGATGAACTCGTAGCGCTATGCCAACAGGTAGCAGATGATGCACTGCACCCACGAAGAATTGAATTTGATTTCTTCTTGAATATGAAGACTTAATTACAAATGTGGCAACGCAACGCATGGTATGGTAATGGCATGTGCAGCTGCACTACAAGACATTGTGCACCACGATGACGACGCTGCCCGGGGTGACGACGCCGGAGCAGCTGCTGGACACGGCGCTGCGGATTACAGGGGTGAAGGCGGCGATGGCGGAGACGAAGCTGGACGAGGCGATAAAGTCGTCGGGCGGCGCCCAGGCGGGTAACCCGCTGATGTCGTCGCTGGAGACGTGCAAGGAGAGCTACGCGTCGCTGGTGGACTCCATCAACACCTCGCGGGACACGCTCAAGAGCGGCGGCAGCAACTCGGACCTCATGACGGAGCTGTCCGCGGCGGCCACCTACTCCACCGACTGCGAGGACACCTTCGAGGAGCGGCCGGAGCTCGTATCGCCCATACCCGGGGCGCAGCGCCACATCAGCCGCCTCGTCAGCAACTGCCTCGACCTGGCAGCCACCATTAAGGAGGAGCCCTAGACGAGACGCCTATATATGATATATATGACGTAGACACTGTAGCCAGGCCGCCGCTTGCATGCATGCATGCATCGACGAAGACACGCAGTGCAAGCGAATTACATACATGTATGTTTATTATGTGTATCTCTGCTACTAGTGTACATTGCTCGATCTGTTGTACATGCCATATGGGGAACGCTGCGGTGCGTTTGTAATAGGCTTGTGTTTATGTTTACTATAATCACTTTCAGCATATATAGGTGTTCTATTTATTAGAAGAACAACCCACCGAGGAGTTTTTATATAAGAAAGACT

>SbPMEI21

GTAATTGCAATGTCCTATCACACGAAGAAGAAGAAGAAGAAGATGCCACCACCACCATGCTATTGCTCCTCCCTGATAACAATAACAATAATCCTCCTCCTACAACAACAGAACAATCCCTGGACTACAGCTGCAGCAGCTTCAGCCATGGCCACCACCACCACCAAGCTTGGGTCGTCACCGTTGTCTGACGTGGTAAAGGACACCTGCGAGAGATGCAGGCAGGGCAACCCACAGGTGAACTACACCCTCTGCGTCTCGTCTCTGTCGTCGGACCCCAAGAGCAGGCAGGCGGACCTCCACGAGCTCGCCATGATCTCGGCCAAGCTGGTGAGGTCAGGCGCAGTGGGCATGGAGGCCAAGATGGCGGAGCTCAGCAGGAAGGAGCGGCCATGGTCTCGCCGGAGGTCCTGCCTGGAGGCGTGCATGGGGGTGTACCACAACTCCCTCTATGACCTCGACGCCTCCATCGCAGCCATCCAGGAGAGGAGGTATGCGGACGCCAAGACGTCCATGAGCGCCACCGTCGACGCGCCCATCACCTGTGAGGATGAGTTCAAGGAGCAGGGCCTGGAGCCACCCATGAAAGCAGAGAGCAAACGCCTGTTTCAACAGGCGGCCATCACCCTCGCCATCATCTCCCTTCTATGATAGTATTGACATGATCCATCTCCCTCCCTGCGATACAGTACAGCTACTGCTTGCTACTAGTGTGCAACTAGCTAGCTATAGATAGTGCACACTCCAATATACTTGCATGTATGGCATCATCTATCGCTCCATGTTTGTTTATCTACAATATATATGCAATCCGGC

>SbPMEI22

ATCGTCAAGCCGGCCTGCTTCCGATCCATCCACCATATCATATCCCTTCTTCTTCAATTCTTCTACTATTGGTTCGTCGTTCCTCGATCGATACCATTAATTCCGTGGGTGTACGTAGCGCCTCTCTCTCGATCTAGCAGGCAGGTGCAGTGCAGAGTCAGAGTGCACTGCTAGATAGTTGATCGTCCATCATATCCAAGCTAAGTAAGTAAGTAATAGCTAGGTCGCAAATGGCGATGATGGGTGCTACGACGATTCGTGGTGGTGGTGCTAGCAGCAGCGTCCGGCCTGCTGTGCTGGTCCTCGGCCTCTGCCTGCTGCTGCTGCTGCTGCTTGGTGTTGCGCAGGCGGTGGAGTTTGAGTTGGTGCCTGATGCTGGTGCCATTGATCCGATGATGATGCCTGCCATGGACGACGTCGACGGTGGTGGTCGTGAGCCGCCGCGCGAGTGCATGACACCGGTGAGCGTGGAGGAGGCGTGCCGCGGCGCGTCGGAGCTGCACGCCGGCGTGGACTACGACCACTGCATGGCGTCGCTGGGTGCCGACCCGCGCAGCAAGGAGGCCGGCAACAAGAACATGCACGGGCTGGCCGTGCTCGCCACCAAGATGGCCATCGACCACGCCGCCAGCACCGAGTCCAAGATCGACGACCTCGCCGAGCTGGATGCGGATAACCAGTCGTCGTCGCCGCAGGCCCGCCGCGCCCGCTTCAACCACTGCCTCGAGCAGTACGGCGGCGCCGCCGACCTCCTCCGCGACGCGCTCGACAACCTCAAGGCCAAGATCTACGGCAAGGCCATGGAGCAGCTCACCGCCGCGATGGGCGCCTCCGAGAGCTGCGAGGACGCCTGGAAGGGCGAGGAGGAAATCCCCGTCGCCGCGCACGACAGGGAGTACGGACGGATGGCGCACATCGCCTTCGGATTCACACACGCAGCCGCCGCATGAATCATCATATCAATCATCATTTTTTTCGCAAAAAAAAATATATCAATCATCATCTATATATGCATCCATCCATGAACGTAAGAACTCCGTCCTCTTCTTCTTGCATCGTCCATCCAACATCCATCATGCATCTCCGTCGACGTACTCGCCGGATCGATGATCCCTTCTTGCAAGCATGCATGCATGCATGCGTCCAATGATATACATATATATAGTAGTATATTCATATCTCCATTATATATGCATCCATCTCCGCTTGAATTGTGGTTCCTCCGATCCGTCGTATATTATTCCATGATCCTATAGTATATATAGTTGCCGATCGACCGGGCTTTTGGTCCATCATGTATGCATTGATGCATGCTTTCGATATATATATCGAGCAACAAGGCTATATCAATTATTATACATTCTAGTAGTCATCGATTACATTTAATTTTGTGTGATTTTTTTCCTTTTTTTTCCTTATGTATGTGCTGCTGTTGGATACATATATGTTGTAGGGTTTCCTCATCAGATGAAGTGAAACACAAGGAATAAAAGCCTTATTCATTTATTATTAA

>SbPMEI23

ATGTCGAGGGCTCTCCTGATGGTAGTAGCCCTGGCCGCCGTCCATGGGCTCATCACGCTCACCGGCGTCGACGCGACCGTGGTAGCGACATGCCTGGCGGCGTCCAACAGCGACCGGCGCGTCAACTACGACTTCTGCGTGTCGGAGCTGAACAAGCACCGCGACAGCCCGGGCGCCGACACCCCGGGCCTGGCCAAGGTGGCGGCCAACGTCGGCGTCAACAGCGCCGGCGGCGCGGTCAACGACATAGAGGCCCTGCTCGCCGCCAAGCAGCAGCCGCCGCCGGACGCCAGGACCAGCGCCGCGCTGCGCCTGTGCGAGCAGCTCTACTACGACATGGAGCTCGCCTTCGCGGGGGCCTACGACGAGATCAACGCGCTCAACTACACGGCGGGGAAGCAGATGGCCGCCGACGCCGACTCGCTGGTGCGCCGGTGCACCGGCGGCTTTGCCGAGGCCGGGCTCGTGCCGCCGGAGCCCGTCGCGCGGCGCAGCGCGTACGCCGTGCAGATCGCCATCGTGTGCACGGCCATCACCAACCTCATCATCAGTCCATGA

>SbPMEI24

CTCGGCACTCAGCTCAAGACAGCACAGCCACCACTCCACTCCACCAGGCTCTAGCGCCGGAACAAGAACAACGCCGGTTGGCTAGCAGAGGTAGCAGATCGACGATGAGGCCTTCGACGGCTCGGGCTCTGGCCACCGCGGCCATCGTCGCGGCGCTCGCGCTGAGCGCCGACGTCGTCGGCGGCACCCCGGAGACGACGTGCGCGGCGGCGGCGGCCCACGACCGGCGCGTGGACTACGGCTTCTGCGTGTCGAGGCTGAGCCACCACCACGACAGCCCCGACGCGGACACCTGGGGCCTGGCCAAGGTGGCCGCCGACGTGGGCGTGGCCATCGCCGGGGACGCCGTCTACGACATCAAGGCGCTGCTGGCTACCAGCAGCAAGCCGCCGGGCGAGGGCGAGGGCGACGCCCAGGAGCGGGCGGTCCTGGAGCAGTGCCAGAGGCTGTACGACGCGGCGGAGTCGGCGTTCGCGGAGGCGTACGACGCGATCAACCGGCGCGACTACGCGGCGGGCAAGGGCAAGGCCGCCGAGGCGGCGTCCCTGGCGCGCCGGTGCGACGACGCCTTCGCGCGCGCCGCACTGCGCCCGCCGCCGCAGGTCGCGCGCTGGGGCGAGGAGTCCGCCAAGATCGCTGTCGTCTGCACGGCCATCACCGACCTCATCGACTGAGCCGAGCCGAGCGCGCGATGGGTACCGTACCGTGTACCAGGTGATGCGCTGGCTGCTCCGTCGTCCGTCGCGATCCGGCGCCGGCGTGTTGTGTGAACTTGTGATCCGTTGGTTACACGCCAGACAAAACGCGGACGTGATGATGGCAGTTACCCGAACTTGTGTTGCCGTTGAAAAATGTAATGT

>SbPMEI25

GTCGCGCACTCAGAGCTTGTCCGGCGGCTGTCCCATCCCAGCTAAAGCAAAACCACCCTCGATCGAGCCCGAGCTGATCATCATTCCTTTCACGACGCAAAGAAGGCACCAAGAAGAATCCCCTCCATCGACGACGAAGGGGCGATGACGATGAGGCCGCTGCCGCAGACCGTCGTCCACCTCCTCCCCCCCGTCGCCGCCGTGCTCGCGTTCGCGCTCATTGGCTGCCTCGTGGGCGGCGCCAGCGCGACGGTGGTGACGACGTGCAGGGCGGCGGCGGACAGCGACGCGCGCGTGGACTACGGCTTCTGCGTGGCGGAGCTGGGCATGCACCGCGAGAGCCCCGACGCCGACGTCTGGGGCCTCGCCAAGGTGGCCGCGCTGACGGGCGTCAACAACGCCGACAACGCCGTGTACGACATCAAGGCCCTGCTGCTGCTCGCCGCCGACGACGGCGCCAAGAGCAGGTCGCCGCCGCCGGACGGCCCGACGCGCGCGGCGCTGGAGAAGTGCGGGAGGCTGTACGACTCCGTCGGGTTCGCGTTCGCCGAGGCGGACGACGAGATCAACAACCGCCGCTACGCCGCCGGGAAGGGCAAGGTCGCGGAGGCGGTGTCCCTCGCGCGCCAGTGCGACGACGCCCTCGCCAAGGCCGCCGCCGTCCCGTCGCCGCTGGCGCAGCACAGCTCGTACAACGTGAGGATTGCTAATATCTGCACCGCCATCACCAACCTCATCAAGTGAATATACTGCTGTAGTATATAGTACTGTAATTACAGGAACAGGAGCACTCACTTTGGTGCATCCAGAATAAATAACGTGGTCGATCGGCCTGACAGGTGACAGGACTTTAAAGTTTGTATACCTTTTGAAAACTTTGTACAAATAATGAAAAAAAAGGAGTTCTGCGTCGAGTAAAGTTCTCCACTCTCCCCA

>SbPMEI26

GAGAAATAAATATGCCATGTATTAATAGTGAATAGTTAACTATTGTATGGGTGGACTGAGAGAAGGCTAAAAAAACCTTACAGCAAGCAAGTGGACTGTATTATTAAACTTGCTCTTAGGAGAAGACAAAGAGGTGCCAAAAAGCAGGATGCCAAAGAAGTTAATGCAATAATGTCAAATTGATGAGTGCCTAAAACGCATCGCAATACGTTCGCATTCGTGACTCCTGAGTATCACGCGCTCTCTCAATTCTCAATAAGAACTGACGCCCACGGCTGCAAATAGCAGGTTATTGCAGTCCATCATCAAGAAGAACTAAAACACCGCAGCTCTCTGCCGTCCCACCCGCCGCCTGGAACTGCAAGATGAGTCCGTGGAAGACCCTCTTGGTGGCCGCCGCGGCCCTCGCCACGCTCCTGGCAGCCGACGCTACCGTGGAGTCGACGTGCAAGGCGGCGGCCGCCATGGACGTCCGCATCGACTACGGCTTCTGCGTGTCGGAGCTGAGCAAGCACCGCGACAGCCCTGGCGCGGACACCTGGGGCCTGGCCAAGGTGGCAGCCAACCTCGGCGTCAACAACGCCGGCGGCGCAGTCCGCGAAGCGGACGCGCTGCTGGCCAGGCCGCCGGGCACGGGAGGCGCGGACGACGCGAAGGCAAGGGCGGCGCTGGGGCAGTGCCGCAGGCTCTACTTCGACATGGAGCTCGCGTTCGCGGGGGCGCACGACGAGATCGACGCGCGTCAGTACGCGGCGGGGAAGGAGATGGCCGTGGAGGGCATCCCATTGGCGCGACGGTGCGACGCCGTCTTCGCCGAGGCCAGGATCCCGTCGCTGCTGGCACGGCGAGGAGAGTACGCCGAGCAGATCGCGGTGCTGTGCATTGCCATCACCGACCTCATCAAGTGATACAACCAGAGTGCATTCATCAGTCTTGTCCTAGCTAGTAGGCTCTTTGAACAAGAATCCAGGGTTTAGACTTTGTTTCCTTGATTTCTTGTAAAACACAGAGACGTTCAAAATAAAGGAGATGCTATCAGCGGAGCTAAGACTTGAGTTGTGCCATTTTAGGACCAATTGTGGTATGGTCTTTTAATTTAGCATTGCCTAAAGCACGGCATGTTACGGAGTATTTGGACCGTGTTGGCACGGCACGAATGCGAGGGCAGTGCTATGCCAAACGCGAGGGCAGTGCTATGCTTAGAATCTTGGCATGATGGGTTGTATGGCTCAGCTTATACCATATTTAGATCACCACAACATAAAAATGGCACATAAACATATCTATAAGCTACAATATAATTTCTAATATTTACTG

>SbPMEI27

ATGAGTCCATCGAGTATCCTTGTCACCACATCCGCCATCGTCGCCATTATTCTTGTTCTTCATGGTGCCGACGCTACGGTGGTGACGACGTGCAAGGCGGCTGCCGAGAGCGATAAGCGCGTCGACTATGATTTTTGTGTGCTAGAGTTGGGCAAGCACCATGAGAGCCCCGATGCAGATATTTGGGGCCTAGCAAAAGTGGCGGCTCTGGTCGGTGCTGCCAACACTGGGAATGTTCTCGTCGAGATCAGGGCCCGGCTAGCGAAGCCGGGAACAGACGCCAAGACGACGACGGTGCTGCGACAGTGCCTTAAGTTGTACGACGCAGCAGATGACGCGTTCCTGAACGCCTATGAAAGAATCAACGAACGCAATTACGCCGCAGGGAAGGAAGAGGTGAGGCGGGCTGGCCGCACCTCTTGCGCTCAGATGCGATGACGCATTCACTAAGGTTGCCAGCCCATCGCCTCTCAACCAGAGCAGCACGTACACAACGAAGATATCCATCGTCTGTATAGCAATCACCAACCTCATAAAGTGA

>SbPMEI28

ATGAAGCTAGTCTCCTCCGTGTTGTTCGCCCTGCTCATCTTGCCGATGTGCAGATCTTCCCCGCTTCAAGACACATGCAGGTCCTTCGCCGCCGGCCACCCGTCCATCGGCTACGACTACTGCATTAGGATCTTCCAGGCCGACAAGGCCAGCGCCGAGGCCACCGACGCGCGCGGCCTCGCCGCCATCGCGGCCAGGCTCGCCGAGGCGAAGGCCAACGCGACGGCCGCGCGCGTCGCGTCCATGAGCGCGCTCGAGGGGGACGCGAGGAGGCGGGACCGCCTGTCCGTGTGCGCGGAGGTATACTCGGACGCCGTGGACCAGCTCGACCAGGCAGAGGAGGAGCTCGCCCACGGCGCGGAGGGCGGCATCGACGACGCGGTCACGCAGCTCAGCGCGGCGCTGGACGCGCCCGAGACGTGCGAGGACGCGTTCCGCGAGGCCGACGACACGTCGCCGCTCGCCGCGGAGGACGCCGAGTTCAAGAAGCTGGCGACCGTCGCTCTCGCCGTCGCGGCGTCACTGACGCCGCCACCAGCATGAACGCCAAGGCCAAAGATTAGTGATTAAGGGCGCCTGTAATTTACACCGTGCTTGATAATACAAGCATGGACGTAATGCAGGTATAATAATAAGCTTTCCTACGGGACAAGAGAGTATTTACTGGCAGCAGAAGATGTTGTTTGAGTTCTTATTTTTTGTTTCCCTTGTTCGTTATGTCGTCGGAGTTTGGTTGTGTGTAGTGCCGTGCATGCTTCGCATGCATGCGCGCGATATGGTGGTGATGGGCGAGTGGCACGTCTGGAATTGTGTCGCGCCTTTTTTTGATCTTTGAACGTGGATTGTTAGCTGTGCGGGACGCTTGAAGCCTTGAGTGGAAGCGACGCGTGCTGCCGGCTGCAGTGCACTTTTGCCTTTGGGCCTCTTCTTTTACTTGTGGGTGGACAGTGGACCTATGTTGTGGCAAGTCCATGAGCCCGGCTGGGCAAAACAGTTGTTAGTAGTAGTCAGCAGCTGTCTTGTCAGCTAGCTTCTACAAGGGGAGCTTTGTATAAGGCAAGTTTGGTTCGTGACCAGGCAGGCAGCTTACCTTTGGAGCAATGATCTTGATTGCTATTGCTCCCTTCATATCCGTAAAGAAATTT

>SbPMEI29

CCGCGCGCGGCACCATGAGGTCATTCCTGGTGCAACCTGTATCCATACTACTACTACTACTCTTCATCACAGCCATCGCTCCCGTGGTCACCGCCGGCGGCTCACCTGTCATCAACGCGACGTGTGCCGCGCTCAAGTCCCTGCAACCCTACGACTACTGCGTGGGCGTGCTCTCCGCCGACCCAGCAGCCGCCGCTGCCACAGACGTCCGGGGAGTGGCGGCAGCCGCCGTCAACATCACCGCGCAGAAGGCTGCGTCCACGTTGCTCGTCATCAACTATCTCGCCGGCGATCTCAACACCTGCCGCGGGTACTACAGCAACATGTTGCAGTCGTTGGAGAATTCCCTCGTCCACTTTCGCGATGGTCGATTCTTGAATGCGTCCCTAGGGATCGCCAATGCCACCGGAGATCCCACGGGCTGCGATTTACTGCTGTTCGAGGGGAAAACGCACAAGGATCCGATTTCCGACGAGAACTATGAGAACATGCGCTTGGTTGACCTAGCAGATGGTATCGTAGATCTATTCGCGAATAAGCGTCTATACTAGTCTAGGGGATCAAGGGTCACACAAGAGTGTGAGGAGAATCTAGATCACACTGTTTTTCCTATACAATATGGCGTGCTTGAAATGAAATGTAATACACTACATACCGAAGGAAAACCTTGATGGTCGTAAAAATCATATTGGTCTCGGTAAAGACATATACTTTCGTGATCATA

>SbPMEI30

ATGGCCTACTACATCAAGAGCTCAGCAATGGTGCCCGTACTACTCCTCGCCGTATTAGCCATCGCCCCAGTGCTGGCCATCGCGACCACATCTGCCATAAACGCGACGTGTACCGCGCTCGACGCCCAGCATTACGACCACCCCTACGCGTACTGCGTGGGCGTGCTCTCCGGCGACTCGGCGGCCGCCGCCGCGACGGACGAGCGTGGGGTCGCCGCAGCCGCGATCAACATAGCAGCGCATAAAGCGGCGGCGACCGTGAGCGTCGTCACCTACCTCGTCGACGAGCTTAGCCTTTGCAGCAAATACTACGGCCGCATGGTGGAGTCGCTGACCGCCGTCCTGGCGGACTTCCACGCCGGACGATTCGACGATGCGGCGCTTGCGAAAGCGCGAAGTGCCTCTGAGGTGCCCAACGACTGCGACGTCATCCTGTTGCAGGGCAGCGCCAAGAAGAACCCGTTTTCTCAGGAGAACATCGACAACGGCAGGTTGTCAGGCCTAGCCCGTGACATCACTGCTCTTGTTGCGAACAAAGGCCCGTCG

>SbPMEI31

TGCTAGGAGTAGGAGAGTAGTACACTTTAACTCAGAGAGAACTCAGAAGTCGGGACACCTTGAAAATGGCCATGGCAGCAACCCCAACCGCGACTGCCAGCATCTTGCTCTTGGCTCTCTTCCTCGCCGGCGCTCACGCGGAGCCGGCCGAGCTCCCATGCGCGCTCCCCGCGTGCAAGACGGTGGGCGGCGGCAGCCAGTTCTTCGACGTGCAGTTCTGCTTGGCGGCACTCGGCTCCGACGGCCGGAGCATCAGCCACTGCATGGACTACCAGGTCTACTCCGTCATCGCTGCCGACCTCCTCGCAGCCAACGTCACCGCCACGGCGGCCAAGATCGACGGCCTGCTCCAGGGGAGCGGCGGCGGCGGCGGCGGAGACGACGCCGCCGCCACGGCGCGCTGCCTCCGGTCGTGCCAGGCGCTGTACGGCGGCACGGTGCGGAGGCAGCCCGGCTGCGCGGCCGCCGTCAGGGGCGTCAGGAAGGGCGAGGCCACGACGTGCCTGGAGGAAGCCGCCGCCGCGGCCAAGCAGTGCGAGGACGGGTTCCAGAGCAGCAAGGTGGCGTCGCCGGTGACGGCGGAGAACCAGAACGCGTTCATGCTCGCCAAGCTCGCCGTCGCGCTGCTCCGCGAGGTCTATGCTAATAAATGA

>SbPMEI32

TTCATGGCACAAGTCATCAACTCCAAGTCTGAGATTCTCCCACTGCACACTGATCAGCTGACAGCAAACGACTCCACCCATGGCGGCGCCGCAGCCTCGAGCCCTCACTACCCACCACCACCACCTTCTCCTCCTCCTCCTCATCGTCGTCTTCACCATGGCTAGTGCGCACACGACAGCGGCACCCGCACCGAGGGCGGCGGCGGCGGCGGCCGAGAGTCCATCGCCAGCGGCGATGTCCTTCCTCCGTGCCCGCTGCGCCACCACGCTGTACCCGGCGCTCTGCTACGACTCCCTCCTCCCGTACGCCTCCGAGGTCCAGGACAACCCCGCCCGTCTCGCGCGCGTCGCCGCCGACGTCGCGGCGGCGCGCCTCCGCGCCCTCTCTGCCCGCGTCAAGGACATCCTCCGGCACGTCGGCGGCGATCCAGCAGAGGGAGCCGCCGCGCTGCGCGACTGCGCGAGCACGGTCTCCGCCGCGGCGAGCCTGGCGAGGCAGTCGTCGGCCGAGCTCACCAAGCTGGAGCCGGACGCCGGTCGCGTCGTCACGACTTCGGCAGGCGACGGGATGAGCAGCAGCAGGCAGGCCAGGTGGGAGGTGTCCAACGCCAAGACGTGGCTCAGCGCCGCGATGGCCAACGAGGGGACGTGCGCCGACGGGCTGGTGGAAGCTGGCGCCGCGGCAGCCGCGGGGAAGGAGGTCACCGCCGGCGTGGCGGCCGTGAAGCAGTACACCAGCAACGCCCTCGCGCTTGTCAATGGCATTCCACTGTGAAAGTGTGCAGTGAATTAGCGTGGACCAATTACTACTTACTAGTGCCTGTGATAGTGTTTAATATTATGAACTTGTCAAGCTGTACAGTGGCCAGTGGGGAACGTGGAGTGGATTGAAAATTTCGAAAAAAAGTCAAAAATTATTGCCTAGTTGTACTGTACAGAGGGAAGGTGGAGTTGATCAGAATTTTTAAAGAACCGTAAAAAAAGTTCTTGCTTAATAATTACACGTTTCCTGTATCTCTTGTTTGTGATTGAGGCAGCGGTTGGAGAAGACGACCCGAGAAAGAAGACAAATAGGCGTGGGGTTAGCTACCAAGCTTATTATGTAAAACACGCTAAAGAATTCTAGTCTAGTGTAATAACATTTAAATAAGAGATGTACTTCAGTCTTCTTCATGCTAAAA

>SbPMEI33

CCACCGTCTCCACCCTCTTTCCAAGGGCCACGATGAGGGTGCCTCTCCTCCCCTTCGTCCTCATCGTTGCCGTCGTCGCCACCACCGTGTCCCTGGTCCCGGCGGTGTGCAACGGTCAAGAAGCTGCTACCGCCGGCGAGGAACATGGAGGCTCTATCAAGCCCTTGTCCCTCGACGGATATGGACCACTGGAGAAGGCTGCCAAGAAGCCCAAGGAGCAGACCTTGAACGCGCAAGCTTCGCCGGCGGTGCCCGCCGACACCTATGATCAGAAACCTGACAAATATGTTGCGTCGTCTCTAGTTCCTGCTAAGGAGGAGGAGGAAACTCCTGCGGAGGTAAAGAAGGAAAAAAAGGAGAAGTCGGATTATTTGGATGAATCTACATCATCCAAGAAGGAAAAGAAGGAGAAATCTGATGATTCTGATGTATCTACATCTTCCAAGAAGAAAAAGAAGAAGGCCAAGACTGACGATTCTGACGAAGATGCATCTCTTACTAAGAAGGAAAAGAAAGAGAAGAAACACAAGAAGCACAAATCCGATGATGATGATTTGGACAGTACATCTCCCAAGAAACACAAGAAGGAGAAATCCATTGATTCGGATGCATCTTCATATCTTCAGAAGGAAGAGGAGAAATCCGGCGGCGATTCGGACGAAGCCACGTCTCTTAAAAAGCACAAGAAGGAGAAGAAAAACAAGAAGAAGAAGGAGGAGAAATCCGGTGAGAACGCCGACGAGGACGACGCTGCGCCAGTCGACGTCTCCACGGACGGGCAATATGTGTCTCCCTCTTCCAAGGAGGAGAAATCCGACGAGGATGACGCCATGCCGGTCGACGTCTCCACCACCACCGGCCAATACGTGTCTTCTCCGAAGTCCAAGGGAGGAGAGCGTCAGGTCTCCACCCCTACGGATGCATACACGTCTCCCGACGAGCTTCCTCCGGCGGCCAAGAGCTCCACCACCTCCGATGCATACGCACCTCCAAAGCAACAGGTCGTCAGCAGCAGCAGCAGCAGCAGCCAGCCCATAGCCGGTGGTTCTCCCGACGAGCTTCCGCCGGCGGCCAAGAGCTCCGCCACCGCCGACCCGTACTTATCTTCAAAGCATCAGGTCGTCAGCAGCCAGCCCATGGCCGGTGGCGCTCCCGACGAGGTTCCGCCGAACGTGGCCGCCAACGGGCAGCCGAACTTGCCGGCGGCGGGGAATAAGCCGAAGCTGTCGATGGGGACGTTATCGGGGATGATCAAGAAGCCGATCGCCAAGTTCCTGAGCCCGGTGATCAAGAGCGTGTGCGCCAAGACGGAGTACCCGGTGGACTGCGAGGCGTCGATCGGCGGGCTCCCCGGGGCGGCGTCGGCGGCGGCGACGGACAGCGTGGGCGTGCTGAAGCTGGCCATGGAGGCGGTGCGGCAGAAGGTGATCGTGGCGATGAACGCGGCGACGGACCGGATGAACGCGCCGGGCGTGGACGGGACGACCAAGGACGCGCTGGACTCGTGCACGTCGTCGTACAGCGACATCAAGACGAGCTTGGACTCGGTGGACGACGCGCTCAAGCGCGGCGACGTCGACACGGCGCACACCAACCTCGACTCGGTGGAGACGGACCTCACCACCTGCGACGACGGCTTCCAGGAGCACGGCATCCCGTCGGTGATGACCGACCATGACCAGGAGCTCCAGAAGCTCGCCAGCAACCTCCTCTCCATCGGCGCCGCCATCCATCGCTAGAAAGGGATCGGAGAAGGGATCAGAAGGGAGGAGGTGGCCGTGTGCAGGCGTGCGTTCATGCATGCTTGCATTGCGTTTTCATCTCAAGAATGAATTTTCGGTTGTAAAATCTGTAACCGAGGAAGAAAAACACCTCGATTAACCATGCATAGCTGCTATGATTCTGTGATTCGACGAGACGATGTTAATTAATCTGCAGCTTTCACTATGTATAACAGTATATATGATTGTGTGAATGTTTCGGAAAAAAAC

>SbPMEI34

GGTTTACGCTTGGGTTGGCAGCATGCACTTAAAAAGCACCACCACACCACACCACACGCCAAGAAATTAAAAAGCAACCGGCCAACGAGTCACAGCATAGAGATGACGCTCCCGCGCCGCCGCCACCTTGTCCTGCTCGCCGGCCTCCTGGTCCTCGTCGTCGTCGCGGCCACCGCCACCGCGTCGACGGATTCCTCTTCCACCACGGCGGTGGCGGTGGACTTCGTGCGGCGGTCGTGCCGGTCGACGGAGTACCCACGGGTGTGCGAGACCACGCTGGTCCCCTGCGCGGCGAGCGTGGGTCGGAGCCCGCGGCGGCTGGCGCGGGCGGCGCTGGTGGTGGGCGCCGACCGCGCGCGCAACTGCTCCGCCTACATCCACGGCAGCCGCAGCGGCAGCGGCGGGGCGATGAAGGACTGCGCGGAGCTGGCGCGCGACGCGGAGGACCGGCTGCGGCAGTCCGCGGCGGAGATGGAGCGGATGGGCCGCGCCGGCACCCCGCGCTTCGCTTGGTCCCTCAGCAACGTCCAGACCTGGGCTAGCGCCGCGCTCACCGACACCTCCACCTGCCTCGACTCCCTCGCGCAGCACAAGGACCGCGGCCGCGGTGGCAAGGGCAGCGACGGCGACGACGATGCCGTCAGGGTGAAGAGGAGGGTGGTCGCCGTCGCGCAGGCCACCAGCAACGCGCTCGCGCTCGTCAACAGGCTCCAGCCGGCGACGCACCGGCAGAGGCTCCTCCTGTGACTCTCTCTCTCTCTCGTAGCTGCAGCTTGCTATGGAAGTCGGTGCTGCTGATTCCTCATCGATCCTCCTCGCTCCATGTCCGATTCCTCACTTCCCCTGCATCTCCTTCGCCAATCCCATGTATGTATGTATGAATGAATGTATGGACTATGGATATGGTTCGATCAGCTTGCTCGATTTCGTAGTTCTCTTTGTCTGTCAGTAGTAATTGTGCAGCTGGGATCATTCGATACCTACTAATGATTGTTCAGGGTTATATATGCTCATGCTTAACTAAAATAACCAGATCTGTGATTAGCCTGA

>SbPMEI35

ATGGCAGCGACAAGGGCATCCTCGTTGCTGATCTTGCTTCTCATCATCCAGCTGAACTTGCTCTTCCACCTCCCAGCTGGCTCCTCCTCCGTCACCGCCGTCGCGCAAGCAGCTGATGAGCAACAGAACACCAAGCAGCAACACCGCCAGCCGGCGCTGGTGCAGTCGACGTGCAACTCGACCAGCTTCTACGACGTCTGCATCGCGGCGCTCGCCGCGGACCCGTCCAGCTCCACGGCCGACGTCCCGGGCCTCTGCGCCATCGCCGTCTCCGCGGCCGCCGCCAACGCCTCCGGCACGGCGGCGTTCCTCGGCAACGCCAGCAGCGACGCCGCCGCGGCGGCGGCGGGCACCCCGGAGGCGGCGGACTACCGCGCGCTGCTCCGCGCCTGCGCCGGCAAGTACGCGGCCGCGCGCGACGCGCTGCTGGAGGCGCGGGCGTCCCTCGCCCAGCAGGCCTACGACTACGCCTTCGTGCACGTCAGCGCCGCCGGCGAGTACCCCGCCGTGTGCCGGACGCTGTTTCGGCGCCGCCAGCAGCGTGGGGGCAGCAGCAGGCCGTACCCGCCGGAGCTGGCCAAGAGGGAGGAGGCGCTGCGGCGGCTCTGCACCATCGCGCTCGACATCATCTCGCTGCTGCAAAATCAGGAGCCCAAATAGTAATATAATGTGATTGTCAGTCGGTTTTTAGTCCATCTTCACGCATAAGAGAGTACTAGTGAAATGGAGCAATGGTCGTTGATCAAGAAATGCATGTCATGAGTTGTGCTCGTACGTGCAATGCAGTGCCGACACGAATACGACATGAATAGCTGTCCGCGGAATGAAAAAGGTTGCTCACGGACTGTCGGACTGTTGTTGTCTGCGGACGAGCATTGGCACGATTTGTC

>SbPMEI36

AAAAAAACTAATTATATAGTTTACCTATAATTTGCAAAATAAATTTTTTGAACCTAAATAGTCCATGATTAAATAATAATTATCAAATACAAATAAAATACTATAGTACCTAAAATCTAAAATTTTTGTCAACTGAACAAGGTCTAAATGAAAGAGAATCCTCTGTCCAAAGTTTCAACATCGTCTAAATGAAAGAGAATCCTCTGTCCAAAGTTTCAACATCCCAACATGGCTACAAAAACCCATCCAAAGCTTGCAGGCAAATTAAGCGGCAACGACACACCAACCTGGCACTAGCAGTAGCAGCACACGCACCCTCCCAGCAAGCAACGATGGCGGCGAGGCTTGTCTCCGTCTCCGTCTCCGTTCCCCTCCTCCTGCTCGTCGTTGTCCTGGCCGGCTCCCGCGCGGCGCTGGCCTCCGAGACCGTGGACCAGACCTGCGCCAAGGCCACGTCGGGCGCGCAGCACAAGGAGCAGCTGGCGTCCTTCTGCGTGTCGTCGCTCCAGGCGGCGCCCGGCAGCGAGGGCGCCGACGCGCGCGGCCTTGCCGCCATCGCCACCAACCTGACGCTGGCCAACTACACGGCCGCCGTGGCCACCATCAAGGAGCTGGAGCGGCGGGGAGGCTGGCCCGAGCGGTCGCGGCGCGCGCTGGCCACGTGCCGCCAGCGGTACATCGAGGCGCTCAACGTCGTGCACAGCGCCGTCCACGCGCTCGCCACGGGGAGGTTCCGGGACTACGTGTCCGACATGGAGGTCGTCCGGAAGGCGGCGTCCGATTGCGAGGACGCGTTCGGCGGCGCCGGCGGGAACGGCATGTCGCCGCTGCGGAAGGTGGACGACGACGCCGACAACCTCACCGTGGTGGCCATGCTCATCGTCAGATCCCTCGGTAATTAAGGGCGGATCGAGGCGGAGGCCATTGGTGTTGCGTTACCTTGCAATTGTAGGCAAATAATCAAAATGTCCTCTTGAGGTTCTTGTGTGATGTGCCAATCAATGAGACTATGGATCAGATAATACTTCACTGACAGTATTACTTGTACGAGCCTGATTTGATATTTCGTCCATTCTAATGGTCTTGTTTGGCTGGTGTTGTCTATTTTTAGGGCTTGTTTAGTTCCAAAAAATTTTACAAAATTTTT

>SbPMEI37

CACACCTGAACTTTCAAAAAGCTTTAGGGAATCGAACCAAGGAAAGTAGGAAACCATGAAGCTCCTGCAAGCTACTGTCTCCCTGGTCTTCCTCCTCGCCTGTTCCACGTCCAACGCTTCTGTCTTACATGACGCCTGCCAGTCCTTCGCCGCTATCCGACACAAGGATGCCGACTACAACTACTGCGTCAGGTTCTTCCAGGCCGACAAGGAGAGCGCCACCGCAGACCACCGTGGCCTCGCCGTCATCGGAGCCAAGCTCATCGAGGCAACCGCTAAGAGCACCGGCTCTCTCATCGCCACCATGCTGACCTCGGAGAAGGATAAGGAGAAACTCGGTTGCCTCGTCGCGTGCGGCAAGGGTTACTTGGACGCCATGGACGAGATCGGCAAGGCGGCGAAGGGCATCGTCTCGAGAAAGGATGGGGGCGTAGAGGACGCGGTGACGGCACTCGGCGGGGCGCTGGACGCGCCCTTGGACTGCGAGGATGGGTTCCAGAAGCTTCACAAGCCGTCGCCACTCGCTGCGGAGGATGCCAGGTTCCGCAAGGAGGCGTCCATCACCCTGTTTGTAACGGGGACGTTACTTCCCCAAATCAACAGCTCTAAGTTAGGGATCTAGAGTCTTTTGGAAGATCAATAAAATTAATTACTAGTATATGAGATTTTGTAAGGTTGGAAACTGGTATCAATCAATAA

>SbPMEI38

CCCTACGAGATAGTCCAAACACTCACTTGAACTTCGATTTGCATAGACAATCAGGTCAGCCAAGGATCGGAGACCATGAAGCTTCTGCAAGCTCTGTGCCCTCTCGTCTTCCTCCTCGCCTGCTCCACGTCCAACGCTTCCGTCTTACAAGACGCGTGCAAGTCCTTCGCCGCTAAACACCCGGAGACCGGCTACTACGCCTACTGCATCAAGTTCTTCCAGGCCGACAAGGGAAGCGGAAGCGCGGACAAGCGTGGCCTCGCCGCCATCGCCGTGAAGATCACCGGGGCAGCCGCCAAGAGCACCGCCCAGCACATCGCCGCCCTGCGGGCCTCCGAGAAGGACACGAAGCGGCTGGCGGGCCTCAAAGATTGCTCCGAGGTGTACTCGCAGGCCGTGGACCAGACCGGCGTGGCGGCGAAGGGCATCGCGTCGGCCACGCCCCGGGGCCGTGCGGACGCGGTGACGGCGCTCAGCGCGGTGGAGGACGCCCCCGGCACCTGTGAGCAGGGGTTCCAGGACCTGGGTGTGCCTTCGCCGCTGGCCTCGGAGGACGCCGAGTTCCGGAAGGAGGCGTCCATCGCGCTGTCTGTAACGGAGGCGCTGTAGGCATCGTCATAATCGATGACGACGTGATGAATTATCTAAGTTAGGGTGTAGAATCCTTTTGGATATGTTAATATACCCTCTGGGTTTTTATGAGCTCAGATAATTT

>SbPMEI39

TCGATCACACATAGCCAAACACAAGCAAGTGTCGGCAACAAGCTAAACACAAGCAGCAACAGCAATGGCAGCAGCAGCCACCACCAAGAACGTCATGGTGCTCCTCCTCCTAGCACTCCTCCCTCTCGCCACACTCTCCTCCCGCGCCGGCCCATCATCGGCTTACAAAAGCCACGGCCACAGCCACAGGTCATCGCCGTCGGCCAAGCACCCACCGCCGTCTCCTTCTCCTCCGTCCTCACCACCTTCTGCTCCCTCGCCTGCTCCGGCGGCGACCGCCGCGCTAGTGCGCGCGACCTGCAACTCCACGGCGTACTACGACCTGTGCGTGTCCGCGCTGGGCGCGGACCCGTCCAGCGCCACGGCCGACGTGCGGGGCCTGTCGGCCATCGCGGTCTCGGTCGCGGCGGCCAACGCCTCGGGCGGCGCGGCGACCGCCGCGGCGCTGGCGGCGAACGGCACCGCCCCGACGGGCACCGCCGCCGCTGCTTCCAGTTCCACGGTCGTCGACGGCACCGTGCAGGCGCTGCTCCACGCCTGCGCGGCCAAGTACGCCAGCGCCCGCGACGCGCTGGCCGCCGCCGGAGACTCCATCGCGCTCGAGGACTACGACTTCGCGTCCGTGCACGTCAGCGCCGCCGCCGAGTACCCGCAGGTGTGCCGCACGCTGTTCCGGCGGCAGAGGCCAGGACAGTACCCCGCGGAGCTCGCCGCCAGGGAGGAGACGCTGAAACAGCTCTGCTCCGTCGCGCTCGACATCATCGGGCTGCTCTCAAACAGCAGCTAGCCAAGCTACCCTGAGCTATATATTCCCATGCATACATCCACACTTGCTTGATGAATTCTTTCTTTCATGTCCCTCGGATACATATATTAAATTAAATGTATAACAACGTGTAACAACTTAATTAGCTAATAACGTGATCCTTCATCCTACGTGTACGTATGTTGTCTCTTGGTTTTGTTTTGGTAGTAAATACTACTAGTAGCTAGTAATCCACTTGCTTCATGACCTGTGTACGTACTCCAATGTTAATCTGAGTGGTTAGAAGATTTCATTGCAATACATGATCGAGTTATTAGCTTGAAGAAAGGTGTAGTGTGTTTTAGAAGGAAAAAACAATACGAGTTTTGCTTTATCTCTCTACTCACAACTAATGATGTTTTGAGACATGTTTTGGACGACCAAAGTCCTGATACATGTTTACAGTTTTGGTTAATGATTACTCGTTATATTATACTTATAACAAATAAATACAGTGTTTATATATGATACGTACGTGACAATTTTTAT

>SbPMEI40

CATGCATTGTGGTGGCTGACCGTGGACTCCGCCACTTAGAGCTTGTTGGAGTCAACAATGGCAGCCTACCATACCATGCATGCATGCCCTAGCTTCTCCTATAAAACCTCGCTCGTCATCCTCCTCCTTTCTTCACTCCAACACAAGCAAAGCAAGCAGCTCAACCTCACCAAGACAACAGCTGAGCCAGCAAGCCAAAACCAAGAGCAAAAACCGAAGCTGCTCGTGCAATTGCTACGTCAACAATGGCGGCCAGCACCAGGAAGGCAGCACTCGTGCTAACCCTAACAATGGCGCTCCTCGCTCCGAGCATCCTCGGCGCCCGCACCAGACCGCCGTCGTCTCCGCACCATAGCCAGGGCCACAAGCGCTCGCCGCCTCCCGCTTCTCCTCCTCCGCCGCCGGCGCCCGCCGCACCCACGGCCGCCGCCGGACTCGTCCAATCCACGTGCAACGCGACGGCCTACTACGACCTATGCATGTCAACGCTGGGCGCCGACGCATCCAGCGCCACGGCCGACGTGCGAGGCCTCTCATCCATCGCCGTCTCCGCCGCCGCCGTGAACGCCTCGGGCGGCGCGGCCACGGCCGTCGCGCTGCTGGCCACCGCCGGCGCCGGCGGCGGCAACACCACGACCGCCGTCGACGGCACCACGCAGGCGCTGCTCCGCACTTGCGCCACCAAGTACGGCGAGGCCCGCGACGCGCTGTCGGCCGCCAGGGACTCCATCGCGCAGGAGAACTACGACTACGCGTCCGTGCACGTCAGCGCCGCCGCCGAGTACCCGCAGGTGTGCCGGGTGTTGTTCCAGCGGCAGAGGCCTGGGGAGTACCCGCCGGAGCTGGCGGCCAGGGAGGAGGCTCTCCGACGGCTTTGCACCGTGGCGCTAGACATCATCACGCTCCTCACCAACAACACCAACTGATCATGGTGTGTTAAGTTGCCGGTGCATGATGCATCTACCATGCATAATATATATGCAAGTGATACCAACCTTAATTACTTTATTACTTGATTCATTAGTTATTCATGTCCAACAGTCCAAGACCACAATATATGTATGTATGCAATGTAACGTGTAAGGTAAGCAGTCCTTCTAAAAAACGTGTAAGCAGTCTCGTGAGTCTCCATGTAATGACAGGAGACTCCTTCTCTATATTTATATATGTCCGACAAAGCTCTTGCCGGCCGGTAGCTTGTTGAAGAAAAAAAAATAACACAGGAGTCATGGTTTTTATAATTTCGTTTGTACTCTATATAGGACATAAGTCGTTACTCCACTTTATTAAGTACGTATGTTTAATTTTTAACAAGATGCCATGAGGATGTGCCAATTTCATTGAGGATATATGTAAGGGTTTTTTT

>SbPMEI41

TTCTCATCCAATCGACACTAGAATTATTCAAACACACAAACACAAGCTTCATCCCATATATATAATATAATACAACCTCATTAGTAGTAGTCGTCTTCAGAGTCTTCTCACACACAAGCTACAAAGATCATCAAGCTCATCATCATAACATGGCAATGGTGGTGCTCCTCCTCTTGTCCCTGCTCCCTCTGAGCACCCTCGGCTCTCGCTCCGGCCCGACGCCGGCCGTGCCGCACCACGGCCACGCCGGCCATGGCACCCCCAAGCACTCCTCGCCGCCTCCCCAACCAACCACGGCGGAGCTAGTACGCAGCACCTGCAACTCCACAGCCTACTACGACCTGTGCGTGTCCGCGCTGGGCGCCGACCCGTCCAGCGCCACGGCCGACGTCCGCGGGCTCTCCACCATCGCCGTGTCCGCGGCGGCCGCCAACGCCTCGGGCGGCGCCGCCACGGCCACGGCGCTCGCCAACGGCAACGGCACCGCCACGTCGTCCAACGCGCAGGCGGCGGCCCCGGCGGCCACCGCCGCCACGGCGCTGCTTCGCACGTGTGCCGCCAAGTACGGGCAGGCCCGGGACGCGCTGGCCGCCGCCGGTGACTCCATCGCGCAGCAGGACTACGACTTCGCGTCCGTGCACGTGAGCGCCGCCGCCGAGTACCCGCAGGTGTGCAAGGCGCTGTTCCGGCGGCAGAAGCCCGCCGGCGGGCAGTACCCGACGGAGCTGGCGGCGAGGGAGGAGGCGCTCAGGCAGCTCTGCTCCGTCGCGCTCGACATCATCGCGCTCGCTTCCAACACCAGCAGCTAGCTTGTCTCGATCATGGCCGGTCTCGAGAGATCTCCATTACTGGCTCTCCCATATATGCATGCATCCATCTTTGGACGAAGTGTGATCTGCTGATCGATGGATCGGATCTTGACGCCGATCGACGTACGCCGTCGATCGTATGCGTGTAGTCTCTACTCTCTAGTGATATATGACTATATATATATGAGTCTACGTGTAATGACACGAGTACACGTCTTCGTCGTACGTGTTTAGTTTTGGCACATCATATATATATATATATATATATGATGTTATTACTGCACTGTTAGCTTGTTAAACTGTATGTAACCAGCGTACTCTAGTGCTGAGATGAGTTGGTCATTACTACTGATGCCTCGTTGTGTTTTTTTTTTCATGATGGTGCACAGAAGAGGAAACATGATGTTAGACAACGATTTGATGGCCGGACGCAGGATGCTGAAATTATTCCTATAAGGAAATAGCTAGAAAGAGCTGATGAAAGGATCGGAACTAGTATTATAAGAAAACGGTAATCGTATCCGATTCCAAATATTTAACATCTGATACCTTATATGTATCTAAATACTCAAATTGTATATTTATTATCCTATCCAGTATGGTTTACACTATCTGTATTCGAATCTGAATCCTAACAAAAATATGAAAACAATGTAATATCAATGATACCCATTCGTATCTGATTCGTACTACTAGTGATGCATGTAAGTGGCCAACAACTATCCTCAAATTATGCAAGACTAACTCACATAAGGTAACAAATAAATAAATCTAAAAAGAGCAAGTGGTTATATTATCTAGATTGGACCAAGAAGAAAAGGGGAAACAAAAAAAAGCTGAAAAGAACACTGTGCGTGCTACAGTGACCTCTCTCTCTCTCTCCCAAAACACCTCCTAGCTAATTATTAAATTAGATACAGCTATGTGTTGTTCATCACTTTTCTGCTACACAATATATATATAACATATAAGTAACACCTCTTTGTTACGTTTTAGCAGGCACATGCAGCGGCATAAAAGGTGCTTGTACAATAACAATTTCCTGAATTATTTGAGCAAGCATGAGCACTTTGCGTTGCACTGCATGTGATCTCCGTGACACGGTTAGGAAATGAAGGGAGTCGGTCCACTGATCAAACCACTAAAAAAAGGATTAATAACTAAAAAGCTCGTCCCTGCTTTACAGTCTTGAAATAACAACCAATCAGCTTACAACTTCCTCTGGAGCACAAGAAAAATATTAGAAAAATCCATATCTATGATGAATCATTTTTGTCACAGTAAATATGCCAATATTGTCATTGAAAATCATATGTGAATGTGACGAAGTTATATGAAGAAATCTTTCTCGTCATGTTTGTCGCGTCACATATGAAACTTAATGATAATTTAGCATAGCATTGTTCATAGATAAGCATTATTAGAAACATGGCGAATTGGCTACATGTATGAGCCACCTTCTTCAAGTGCATCTCACCATCTTGTTAGGGCCATTCTTTAGCTTCAGGATCAACTCCGGTGCTTTCCTTTCCTCTTTTCCGTCACATGCCAGTAGTGGGAATACAAACACCTTGGAATTGTCTGTTAGGGGGATTTAATGAGTTTGCTAGAGAAAACAAGGTCATTACAAAAATTTCAAAGAGCCCAACCCACCCCCTGGCTAACACATAGACCCTTAGCTTATTTCTACTACCATATTCTCCCAACCAAAGATCTCGGTAGGTCCTTATTAAAAATGGTATGGCATTTCACCCCCTAGGTTTTCTCTCACACAACACCAAACAATAACGGCAAAGAGAGGAAGAGAAAGAGGAAGAGAGAGGGGGGAAAGAGAGAAAGGAGATGAGGGACTGGCTCACTGTATTTGGATGCTACTGTGAAGATCATTATTGAGGTCTTGGAGGCCGAGGCTCTGACATACCCGCCTACTGCAGCAGGAACTCACCACCATGGCCATGACATTGGGAGAGAAGATGGGAGTGCTAGCATTGGGAGATGTGGCGCCATGGTGGAGACGAGCATTGGTGGATCTAGGGCTCTATTTTAGAGGTGGGTCTTTTAAGAGGTCTGTCTTTGAAAATATAGGTCATGCCTCTTAAGAGATTTGATGTTAATTGGTTTTTGTATGTCTGTTTCCTACATTTTTTTGGGGGTGGTATATTTTTTTGGCTATATCTATAATGGGCCGAGCATGTGTTGAGATTCGATGTTAATTGATTTTTAGATGTCAGTCTACTCTAACATTTTTTGCAGTACACTACACCACAACACTAAAACAGCATCAGACATCTGTTGCTAAAGATAAAAATTAGCATTGAACGGTCCGTTGCTAAAGGTTTTCCTCGGATCCTCAGTCGCTGAAATTCAATACTTCGATGGATGAACCGTTGCTATAAGTATTTTGCTTTGGCGTTGAATCGTCCGTTGCTATATATGAGTAACTGAGTCGAATCATCCGTCGCTAAATAAATTATCCAGTGGCGTGATGTTTTCGGCCCGTTATCGTGAAAATTATTTGAGGTCATTTAGTGTTTTATTTTTAAAAGTTTGACTAAGTCAAATATTTTACATTCATTTTTTAATTTTTTCTAAAGAACAAACTTTGAAATTATATATATAAATATATCATATATTAAATTGGTATATAAATATTTCATATATTAAATTGGTATATATATTAAATATACCATGCGAACCTGTAAGGTTTTGAGATTTTTGAAAATTATCTCTATGTGCATTGAGGTCGACCTTATCTAGTTAGGGGTACTATTGGAAATTTGCTCTAAATGAAACTTTAGGGTTGTTTTGGCCCCTAGACTGAACGACTCAGAGAAAACTTTCCCCTTTTCATTTTGTTTCCAGCGGCGGCGCTCCTGCTCCCGGCTCCCGGCAGTGCGGCGCTCCTGCTCCCGGCGATGCGACGCTCATGCTGGCTGGTGGGTCTCGGCCTCGGGGCTCGGGCTCGGGCTCGCGCCTCACGGGACGCGGCAACGCGCGCGACCGCGGCAAGCCGCGAGCGTCCACACCGAGTCGGCGAGCTACCGGCGATGCGACGCTCTAGATCCCGGCGGTAGGCTGTGCTGCTGCTGGCGAGTCGCGGGTTCGCAGTCGCAGCCTCGCAGAACACGGCATCGTACGTGACCGTGGCAAGCGGAGGGCAGCCGCGGCAAGCCATGGCCCATGAGCTTCCACGGCGAGCGCGCAGGCATCCAGTGCACGGCGAGCCGGTGAGCAACTGGCGTTTGCGGAGCTTGAATCCAGCATGCGGCGGGCGTGCAGATCCAGGCCTGCAAGCGTTCATTGCCTCCATGGGCCCTCCTGCTGTTCTTCAAGGAGAAGCATAGCGGCAGGTGAGCATTTAAGTTATCTCTGATCTATTGCTTTGTAGTTTGTAGTTTGTACTACTTTGTACACTGATGCTTGAGTGTAGTAGTGTTCTCTAGATCTAAATAGTTTAGGATTGTTTCTAAATTCTAAGTGCTAACATGTCTTCTCCATATATACAATTATACATATGTATGAATCTTATAGTTGTGCAATTAAACTTGTGTTGTTTATCATATTTTAATTATTAATTATAGCATTTTGTGTTGGATGCGAGAATGCCGTGGCATTTCCTTTCTTTTTGGACATTTTTGTTAGTTTCAGTGGACCGTGGAACCTATGAATAGTAGGCATTGCTAGCAAGCTTTTGGTAGAATGTGCCAAAGTTTGGTCTTTTTACCAGGAATGAAATTAGACTACTGCTCTACGTATTTCTTCTGTCTTTTTCCTGCAAATGTCATTTAATTAGAAATAGTAAAATATTCTGCTAAGAGTGGCAAAGAGACAGAGTGATCACTTCTACTGAATCGGTCTAACAAAGAGTAGTATTCTGTTTACTCTGTTCTGGATGGATACTTTAGGTTTATACATTGGACTGTGAAAGGCAGGAACTGATATTTGGCCGAAATACTTGAAACCATTTCTGGAATTTAGGTTTGAACTGATATTTGGCCGAAATACTTCAAACCATTTCAAACCAAGGCCTTTCTCCTTGCTGTGGTGTTTGTATTCAATTTCTCCTTTCATTTTGAATGCATATCTGTGTTGCTCTGGGATATTTAATTTTCAAGTGGTGATCTGTGATGTGTTCCAGAAATTAGAAGATACTAGGCATTAACATGTTTAGTTTATATGATATAACCATGTACAATTGTGAAACCCATCATCAAATGCTAGTTATATACTAGGCATAGCAGAAAGTCTGGAGTAGAGATAGAGATGATCAAAATATAACAACCTAACATGAGGTTTAGTAAAAATGTATGGGTGGGTCTGTATTGATCCTCTCTGGTTTGTGCAAAAGTTCTTTATGGCAGATCAAATGGTTTGGGAGACATAACATTTGACTTGACCCGTACATCTGATTTACTGACAATAATCCTTGGGGGTGGATAATGATGCCTAGATAAGCTGTTCTTTTCTAAAGTTTGTTCAGCAATTTGTTAAATCTCTAGTATGGCATGGCTAGATTATTTTTCACCAATATATTATGAACAAGAACAATTGATTCTCAGTTACTATCACAGCTTGATGCCTAAAAAGGTGTAGAGGAAAATCTGGAGTTTAGTCCGTGAATGTGTCCTGAATTCCTGATCATTAAGAAAACCATGTTTCCTAGAGAGAAGCCTACCCTTCAAACCGAAAACAACTCCTAATGCAGGCAGCTCAAGCATACCAGGTTGCTCTCAAGGTAGTAACTGAAGAAGACAGTAAACAATTGATGTAAAGGCATATTCTAATAGATGTGTATCTAGGTGTGCATTTTATTTGTCCTCACTTAGTTGTGCGTTATAACAAACATGTAGCTGCATAGTGCCAAAAGCTACTTCATTCAGTTGTTTTGTTTTGTAGCTATCCCTGCTAAATGGCATTAGACTATCCGGAATTCGTCGCGAAGCTTCAGACCCAGGTATGTTCTTTTCTGGAAAGGCTAATGTTCATTTGACACTAGTGCTACCTCTGTCTGGATTAATTGATTTTTCTAGATATTTTAGATAATGAGATAATCATATGGGCTAGATATCTTCTGGTGGTGGTGTTCATCTAGCTGGGAACTTAACAGAGTATAGCTCTGTGATTGTTTTAATACATTTAAATTGTATAAAATACCTGAGTGATCTACTCTTATGCTAAAGGTGGTTGTTGTTCTGCCTTAGCTGATGTGCATTTATCCTGTGTCTACGGTAAAACCCTTCTATTTCAGCATATGATTCTGCAATCTCATTAACTTGCGTGAATCATAAACATATCTGAGTGATGCTTAATAATTTGGCACATATGCCTTTTGGTTATTTGATTTGCATGGTTATATGCCTTTCGGATTGTGTGTCATAAATGCATTAGGATCTATGGGTGAGTTAAATTTTCATCTGGAGACTATCATATTATTTTGGATCAATTTGGACTCGTGCATCTTGTCATCATCGTTGAGTACTATCATTTTAGTTATAGTGCAAAATGCAATTGGGTAAGAGTGGTCCTCTCTAGTGCTATGGGTCTATCAATAATGGTACTGAATATATTCAGGTTTCAGTAAAAAGAGTGATGCTCCTTCATACTACTATCTTTCTTGTTTCTTTAATTGTTTACCTTGACATTTAGTTCTATCTATTTTGGTTGTGCAGGTGCTCTTCCTATCCATGTAGTGGGTCCTTGGCTACTAAAGTTTCACATTTTAGAAGGTGTAGAACACTTTGGTATTGTACAGTTTAGTGATTTCACTTAGCACTAGAGGATGGTTGTGTATTTTAAGCTTTCACCTAATTTTTGGTGGCTTCCATGGATATCAAGTGCTAGTTACATATGTGGAGACAAGCATAGTTTGATTTTTAAGTGACATGATTGCATATGTACTTGAGTTTGACAATTGATTGAGTATGTCTGATGTCATCGTTTGTGTATTAATCATTTTAATATTGTATTGGTGG

>SbPMEI42

TTTCCCTTTCTTTTTTTCAAATTCTTTTCTGTTTTCTTTTCAACACATTTTCTAAAAGAGCTTTTAAGCATAAAGTAAATAAAATAAAGATCAGCACAAAAATTATTATGCTCCAGCATGAAATGCACAACCATGTTTTCTAAACTTATAATAAATTTTAATTTTGACCAAAACTATTTTATTCACGATTAAATGCTTACCAAAAAAATTACTTGAATAAACCAAATTAAATCCTATTATTTAAAAATCAAATTTCGGGTGTTACAGCAAGGAGTTCCATAAGTTCATTATTGTTGAGTGAGGGTTAAGATTTGTTTGAGTAGCTCAGAATTTAGCTTGGGATTTGGTTTCAAGGATTTGGTCAGGAGCAAACTTATATTATTAGAGGGAGGCCTGGACTAGCAATGTGTGGAGTCTCTCTCAGGACATTGTTAGATTCATCATGCCTTCATATGGTTGTGGAAAAGCTTTCACCGGCCTAAGCATTAATTGTTCTTTTTGCTTCTTCTCAAAGATAGGCTAAGTACAAATACAAGTAATATTCTAAGAAGAAAGGATATGCATCTAGAAAGGGACAACAGTGTTTATGTCTCATAGATAATGAAGAGACTCAAGAAAACGTCTTTCAAGACTCCAGAACAACTGTGTTGCAGAAAAACTCAAAATCAATTCAAAATGAGCTCCAAAAGTTTTGAAACTTTCCACAAACCTTGACTAGGATGCAGGGATGAAATCGGTACGGATATTTTCTGACCGTATTCGAGATCGAATTTGTTTAAAGGGGAGATCTATCCATAACCGAATATTTAAATGTTATATTTATGATGTTGACATCCAAACGTATCTTATCCGATATGGTTGAAATTCTCCGTATTCAAATACGAATCCGACCAGAAATATGAAAACAAATATGATATTGATGATATCCGTCTAAATATTAGATGGCAGATTGAAAAACAAGCTCACTTTGGGGTTTTTGCGATATTACAGAGGACATGATGGTGCTTGTGGAAAATATGGTTAAGGCAAAGCCTTGACATATTCCCAAGACCTTATAGCGCCTACGAGTCACAACAAATCAATCTAAAAAGTGCAGCTTAAAAATCGCTAGCTAGCCGAAGGAAATAAACTAGGGATGGAAGAGATTGTATAGCCACTAGGGTGAAATGTTTCAATTTGGTTTCCAAGAATTGGATGGTTGTGGGCCCTTCATAAAGTCGTTTCGCCTCGAAGCAAGCCCTCCAATAATGCCACATGCCACCCCAAGATCGTCCCCCAGGTTTTGAGGCCCAAACTAGGCAAACCTACTGCCTGTGGTTTTGAGGACCAAACCTCTAAACCCACATGGAATCGGGTAGCCGATACATCTTCTCCATGATGTCAACATGTGTATGCTTGTATCGAGTGTACGATCCCAAATGCTTGGGCGCCCAACTTGACTTGGTCTGATGGTTGTCTTGACTTCAGTCAACACCACTCCATCACCATCCTCGCATATATGTGCTTGCTTTTCTATAGTCCATCCTTTGGCCTTTGGTCCCTCGGTCCAAGCCCTCGCGTCTACCCTTTACTACTCCCTGTCCATCCCGCGCTGACTCCTCGCTAGACCTTCTCCATTGCCATCGACCGTCTCATCCTCAACCCTGCACATCACAAAACCATGACAGATGTTGCGCACACAATTGATGGCAATGTTTGACTCATGCACACTTAACCAAAGCTCTAATCACCTCTTGACAATCACTAATCACTCAAACATGAGCATATATCAATCAAATCCTCCCTTGATGATGTTGTCAACATCGACACAAAGATATGAATCAAAAGCAAAAGAAAATTGTGAACTCATCCAATGACCAAATAAAAGCCAGTGAAAAGCAATGAACAAGGTCCCTTGAAATGAGAAAATTGGGTCCACAATGAAATGGTCAACGACTGACACAACCAAGAAGAAGCAAAGCTTGGTTCCCAAAATACATGGGCAAAGGCTCAACACAACCAAACAAGAGAGCCAGTCCTCAAGAAGAGGCTGAAAGGGCTCAACACCTATAGCATCAAGTCTTGTCCGTTATTTGCTAATAATTTTCTTCTAAAGCGTGCTCTTTTCCTTTGTTTGATAATCATCATTTATGTTGAAAAAAACTTGTAAGAGTTGGCTGTGTACCTTTGTTAAGGCTGGAATTATTCCTTTTTTCTAAAAGAAGGTCTTGTCCAATCATAGGTTTCTTAAGTAATCATAAGACATAACACTTTTTTTATTCTACGGGAACAAGACTTTATAAAACAAATTTTGTACAAAAATAATTAGCGCCGGCACAACATCAACGCTTCAATTTAATGAAAATTTTTTGTTTTCTTATAGCCAGATTAAACTGTGATGATGTCATTTCTTCTTTGATATATAGTTAGGGTCACTTGTGCAGAAGTTAAATCTCTAGCGCCTTTCACACTTTGTAGTTTGTACTATCACATATATAGTATCAACTAAAATATGAACGGAGATAGTATGACCCAATTCGAGGTTGATCATATGCCAATAGAAACTGTAGAACATCTGATGGGCAACAAGAATCGTGGGTTGTACTTGTATGTAATGTAGGCAATGCACCGGTTACATCGGTGGGAGCACAAAGGAAGAAAATGAAATGGTGTGTATGCTTCCCATTTCATGTATATCAGTTAATTCAATAAAACGATCTATTGCTTTGTCTGACACTTGTGCATTTTGATGTCATCTTGCTTAAAAGACAGTGCAAGAAACTTGGCCACCAAAAGCTGGTAAACTTCGAGATTATAGGTATATCTGACTACCAGCTTCTGAGCCGGCAGTAGCAAGCGCATCAACATTGTCAGTTGAGGCAAAGAGCCAGTAGTGCTCAAGGGTATATCATTGAACCGGCAGTGTTGCTCAAATCAACATTGACTGCTGGTGTAAAGAACAGATAGTGTTGCTCAAATTAACATTGTCGGTTTGTGGTGCAAATCAATAGTGAAGTTTGTTATTGCTGTCAGTTTTTCGAAACTGACGGCCGGTGATAGGAAAGGCACATTACTGCTGTTTTTCTTCCGCCGGTTTGAATTGTGGCAGTGATAGTGGGGTTTGAACCGGGAGTGAACGAAGGCTTACGTACTAGGCAGTGTAGTACAACATATAGCACCATTTATGGGCTAGCTAGAAAATACATTTTATTTTCATGATCACATTGCACATTATATATATATGAAATAATCATACATAATAAGTACTAACGAAGACATGCATGACTCCACTTGGAGGATGAATCTTATTTATATAGTAGGGGCATAGAGGGCCGTTGATGACTGACGAAATGGGATGGACGACGTACAGAGCCATAGACTCTTGTTTTGAGCACATATTGTTTTGAGTACATATACAATTACCTCTGAAAAACTAAATCCAATGTTTGGTCGAGTGCAATGATCATGTTTTTATTCTCTGTATGTAAATGTGGGTAGTAAAATATATACTTCTATAACGGTACATATAAATATATAATTAGGATCAAGCTAGGAAAGACATCATAATAAATCGTAGTACAAAGGCAAGATAATGTTTCCTTTCAAAATAAATTGTACTATAAGGCTAACTCTCCATATATACAGCAACATTTTCGGAATCATATCATGCATGCGAAATCACACAGTGAGCAGCACATATATTTATAGGTCCCTCAGTGCTTATTAGTTTCAACATCAAACTCAAAGCCTTGAGCAAATCAACAAAGATCAAAAGAAAGAGATCGATCGAAAAGCAGCCAAGATGGCCTACGTCGCTGCAGCAGTGTTGGCAGCAGTGTCTCTCACCGCCTTGTTGTTCGCCGGCGGCGAGGCCTGCGCCAATGTCCCGTCCATGACATCGACCGAGGCGTGCCAGCAGACGAACAAGTGGGAGCAGCTGTGCCAGCAGACGCTCCAGACGGCGCCGGACACCGCCGAGGTCACCGTGTTCGCGCTCGTCGCGACGAGGCTGGCCAAGAGCGCGTACGAGGACACCTTGTCGGCGCTGGACCAGATGCTCGGCCCCGGCAACCTCCCCGGCGCTGAGCGGTTGGCCATCGACAACTGCAAGGAGACGTACAGCACGGCGCTGAGCAAGATGGCCGGCGTCGTGGACCACATGTCCGCCTGCGACTTCTCGCTCGCCAGCAAGGAGTACATCGACGCCGAGGCCGGCGTCCGTTCATGCCTGGAAGGGCTGCAGCCGTACCAGTTCTTGCCGCTGTTCGGCAAGGTCTCTGCAGACCATGATTTGACCTTGGTCGCGTATTTGCTAGGCGCTATCATTGTTGGCAGGTAGACTCTCTCTAGAGTAGACAGCAGCAGCGGCAGGTTTAATTAGTTTGCGTTGCATTGCAGACGATCTTGTGCTTTTCAATAAAAAAGATAATGAGTGGTTCATGATCGACTTTTGTCATTAATTCTGATGAGTTAAATTTTGTTATGTGAACGTCGTTGCTTATAA

>SbPMEI43

AAAGCCTTGCAAAAGAAAAGATCGAATCGAAAGAAACAAGAAAGAGGAGCAGCCAAGAATAATCCAAGAATGGCCTACATCGCTGCAGCAGTGCTGGCAGTACTAGTGTCTCTCACCGCCTTGTTCGCCGGCGGCGAGGCCTGCAACAACGTCGCCTCCATGACATGGACGGCGGCGTGCCAGCAGACGGACAGGTGGGAGAAGCTGTGCCAGCAGACGCTCCAGGATACGGCGCCGGACACCGCCGAGGTGACCGTGTTCGCGCTCATCGCGACGAGGCTGGCCAAGCTCAGGTACGAGAACACCTTGTCGGAGGTGGACACGTTGCTGCGGCCCGGCAACGCCCCCGCCGAGTCGCGGGCGGCGCTTGACAACTGCAAGGTGAAGTACGGCTCGGCGCGGCGGCTCTTGGCCGGTGTCTCGGACCAGATGTTCGCCTGCGACTTCTCGCTCGCCAGGCAGGAGTACATCGACGCCGAGGTCGGCGTCCGTTCCTGCCAGGATGGACTGCTGCGGCTGCCGAACCAGTACCAGAGCTGGCCCTTGTTCCGCAAGGTCTCTGATGATCATGAGTTGACTGTAGTCGCGTATTTACTAGGCGCTATCTTTCTTGGCAGGTAGACTCTCTTGAGGAGACAGCAGCGGCATGGTTAATTAAGCTGCATTGTGCTGCGGACGATCTTTGTGCTTTTCAAAAAAGATAAATGAGTGGTTCATGATCCATTTCGTCATTGATTGTTGATGTGTCACTATATTATATGTGTTATGTGAACATTGTTGCTCTTCATAATTGGAGTATGCAAATCATAACATTTTC

>SbPMEI44

ATGACGCCGCCGTCGCCGCCGCCGCCGCTGTTCCTCGCCTGCATTCTCCTCACCCTCCTCCTCGCCGCCGCGGTGGCGCCACCGGCGGTCGCCGTCTGCGTTCCGAAGGGTCACAGCAAGCCAGGGGCGCCGGCCAAGGCGAAGGCGAAGGCGAAGCCCAAGCCGAAGCCGGCGCCGCCGAAGCCGACGGCGATCCCGATCGCGCCTGGCGCCGACATCGTGCGGAGCCTGTGCGTGAAGACGGACTACCCTGACCTGTGCACGTCGGCCATCACGAAGCAGCCGCAGCCGCAGCTGCCCGCCGGGAAGCGGCTGGACGGCGCCGGCGTGCTGCGGCTGGCCATGTCCGCCGTGCGCGCCAAGGCCGCCGAGGCCAAGGCCGCGGCGGGCGCGCTCGCGAAGGACCCCAAGACGCAGCCGCTGGCGCGCAACCCGCTGCAGGACTGCGTCGAGTCCTTCGACGACATCGCCTACAGCCTCGACCAGGCCCAGAAGGCGCTCGCCGGCGGCGACCGCGACACCACGGGCACCATGCTCGACACCGTGCGCACCGACGTCGACACCTGCGACCAGGGGTTCGAGGAGCGCAAGCAGCTCACGCCGGTCATGTCCAAGCACGACGCCGAGCTCGCCAAGCTCTCCAGCAACTGCCTCGCCATCGCCACCGCCGCCGGCTTGCGCTAGATCGACCGGCCGGCCACACATATCTATCGTCCGGTCATCTCCATAGCTGTGCATGTGCTAGCTAGCTGCTAGGCAACAACAACAACAACAATATATACCGCCGCCACATTATAATTAATTAACTCTCCACACATCAGAGAAGCTTCAGTGATGCAACAAACTTGTGTATCCATGGATATCCAGGAAGAAGATGTAGTTAGTGTGCAAGCGTTCTGGTTGTAATGTTGCAGTGCCTTGTTTTTCTTGATTTCGTTTTCTTTTTTTTCATTCCTCCTCTTCATTCCTTCAACCATCACGAAGAACTGATTGTTGACTGACGGCAACATGGATAGAAAACTTGATCATGTTTAGACCAACTGCGTAAAACTTAGATCTAAGAATAATACTTCTC

>SbPMEI45

ATGGCCGTCGTCTGCGGCGGCTCCAGCGCCGCCGTCGGGGCGGCGAACTCGGCGCTGGACCAAGTCTGCGAGTCCGTTGGTGGTTCCTACGTCACGCCGGAGCTCTGCGCCTCCGCGCTCTGCTACGACGCGGCGTCGCCGTGCCGCGACGCGCGCGACTACGCGGCGGTGGCGACGCTCGCCGCCGGGCTCCTGGTGCGCAACGGCACCGCGACCAGGGACACCGTGGCCGTGGCCGCGGCGGCGGCCAACGCCACCGCGGGCCTCAAGTCGTGCCTGCAGCTGTACGACGGCCTCTTGCCGGCGCTGGAGTGGGCGGCGGGGTCCGTGGCCGCGGGGCGCGCGTACGGCGCCGCGCGGGAGCTGATGCAGGCGACGCAGTTCGCGCAACGGGCGTGCGCTGGCATGGTCGCCGGCGCGGAGATGCCCAGGGAGAACGGCGGCTTCGTCACGATGGCCAGAGTCGCGCACGCCGTCCTCTCCACCTCCGTTCCAAAGACTGACTGA

>SbPMEI46

TGAGGACCTTGCTTGTCGCCGCCGCCGCCGCGGCCCTCATCCTGTCCGCCGCCGCCGGCGCGCTCGGCGTCGCGCCGGCCGACACCGTGGCCGACTCGTGCCACGCGATCAGCGACTTCGTGGACATGGACTTCTGCACGTCCCGGCTGCGGTCCGTGCCGGGGGCGGCCGCCGCGGACCGGTTCGGCCACCTCCTGATGGCCACGGACCTCGCCGTGGCGAGCGGCGCCAAGGCGCAGGACCTCGCGGCGGCGGCGGCGCGCGACGATGGCAGCGTCGTCGTCGACCCGGGCGAGCGGGACGCGATGCAGGCGTGCGCGTTCCTGTACGGCGCGGCGTCCGTGCCGGGGCTGCGGCTCCTCCGCCAGTACGCGGCGGCGCGGAACTGGGCCCCCGCCCACGCGCTGGTGATGCTCACCATGGACGCCGGCGACGGGTGCGACGCCGCCGTCGGGGGATCTAATGGCAGGATGGCCGGGCCGAACCACGAGTTCGACCAGCTTAGCGCCATGGTCACCGCGCTGCTCAACAGCATCAACTTGTACGATGAGTAG

>SbPMEI47

CACCACCGCAGCCAGCCGCCGGCCGTTGAGAAGCAGCACGGAACGGAACGGACACACACCGTGCGCGTGCGGCGGGTCATCATGATGATCTGATCGTCCAGCAGAACCAGAAAAACCAGCGCCGCCCGCGGCCCCCGCGCGCATGCTCCCGCACTAAATGCATGCACGGCAACGGCGGCGCCACCATCACTCTCCATGAACATCCGGACCCTGTGAGACCGTGCCTCTGCGCTAGCGCTACCAACAACACCTTACCGGAGACGACCAACAGCTGTAATGGCGGCCGGGTCGTCCTCCTGCTCTCCGGTCCTCCTCCTCGCCCTACTCCTCGCCGTCTCGGCCGGCGCAGGCGCAGGCGCAGGCGAGGCTTCGGCGCCGGCGCTGGACCAGGTGTGCGGGAGGCTGGGCAGCTACTACGTGACGCCATCCCTCTGCATCTCCGCGCTCTGCGCCGACGCGTCGACGTCGACGTCGACGTGCCGCGCCGCGCGGGACGCGCCCGCCGTGGCGGCGCTCGCGGCCAGGCTGGCGGCCGACAACGCCACGGCGGCCAGGGACAGCATCCAGGCCGCCGTCTTCTCCCCGTCGTCCTCGTCCTCGTCCTCCGCCACCGCGGCAGCGGCGGCCGCGCGGTCGTGCCTGCAGCTGTACGCGGGCGCGGTGCCGGCGCTGCGGTGGGCGGCGCGTGCCGTGGCCGCCGGGCGGTACCGCGGCGCGCGGGAGGTGCTGCAGGCGACGCAGTACGTCGCCGCGGGATGCGAGGGCATCGCGGGCGACGCCGCCGCCGCGCTGCCTCGCGAGAACGACGGGTTCGCCGACATGGCCTTCGTCGCGCACGCCGTCGTCGCCTCCATGTCCGCCGACTGA

>SbPMEI48

ATGATGCATTCATTGTTAGTTCAACCTCCAAACGGCCACCGTTATTGCGACGAGAACGCAACTCCGGTGATGCACGCAGGCGTCGCGTGCGTGCATTGATTAGCTCACCTACCCCTGTCTCGCCTACCTCCTCCTCTGCGTGCATATTGATAACAAAACCAGGCTTGCAGCTGCAGGTCGCACAGCAAACGTCCACCAACAAGCAAGAGCCATGGGCAGGACCACCGCCACCACGCTCCTCGCCGTGGCCGGCGCCGCGCTCTGCTTCTTCTCATGCTGCTTCTACGGCGGCGCGGCGGCGGGGGACACGGTGGCCGAGTCGTGCGACGCGATCCGCGACTTCGTGGACGTGTCCTTCTGCGCGTCGCGGCTGGGGTCCGTGCCGGGCGCCGCCTCCGCGGACCGGCACGGCCACCTCCTGATGGCGGCGGACCTGGCGGCCGCGAGCGGGGCCTCGGCGCGCGACGCCGCGGCGGGGATGGCGCGCCGGCGCCGCGACGGCGAGGGCGAGGGCGAGGGCGACACGGACGCGCTGGAGGCGTGCGGCATCCTGTACGGGGCCGCGTCGGTGCCCGCGCTGCGGCTCATGCGCGGCTACGCGGCGGCGCGCGCCTGGGGCGCCGCGCGCGCGCTGCTGCCGCTCACGGGACAGGCCGGGATCGGGTGCGACGCCGCGCTCGAGGGCTCTGCGACGGCCAAGGCGCGGATGGCCGCCGCTAACCGCGAGTTCGACCAGCTCTCCACCATGGCCACCGCGTTCCTCAACAAGCTCACCTTAGTCACCTAGGCCTCCGGCGGCGCGCCATGCCACTCGTGCCGCCGTGACGTACCCTGCGTGGGCGTGCCATGCGTGCTGCGGTGACAGTGATGCGGAAGCGGCTGCTTCTTGGGTCGAATCGATCAGACAATCAGTAGTGCAATGAACCACTGATTTCTGTGTGTTTCTTTGTTGCAGTAAGTAGCCTGTACAATCTACAATGCCGTTGTTAAAGTCATGTTGGCATATACGTATTACTGTGAACTCCAGTTGCTGTTTTCCGTTCAGCAGCAGCTCTTTCTTGTCAAAAACAAAAGATTTTCCACATGTCAGCTGCCTTGCCCAAAAACTTCTCGATCGCCATTTTTTGTGCTATAGAGGAGCCAAAACATTCCGTAGAGTTTATTCATCTTCCATCGAAATAATCTGGATATCAAATATCTTGAGCTCGACAACTCTTGAACCGATTTGGGAACTCTTTCCACCAAATCTCTTCATTTTAATTCACATCTGGCAAGCTTTAACAATTACTAGTAGTACTTGATTCCATTTTCAGTGAACAACAGCCAATTGTGAACCAACGCTTGCCAATATCATATTCCATGCATGCATGCACACCAAACGCATGGCATCCCG

>SbPMEI49

ATGGCCGCCTCAGCCTCAACTCCCACGCTCTCTGCTACCCTCGTCGTCTTCGTGTCCGTCGTCATCGCCGTCGGGGCGACCACGGCGCTGGACCAAGTGTGTGGCGGCCTGGGCGGCTACTACGTGACGCCGGAGCTCTGCGTGTCCGCGCTCTGCCCGGACCCGTCGCCGTCGTCGCCGTGCCGCGCCGCGCGCGACGCGCCCGCGGTGGCGGCGGTGGCGGCCAGGCTCGCGGCGGCCAACGCCACCGCGGCCAGGGACAGCGTCCAGGCCGCGCTCTCGTTCTATGCCGCCGCCGCGGGGGACGACGACGCGGCGGCGGGGAAGAAGGCGGCCTTGCGGTCGTGCCTGCAGCTCTACGGCGGCGTCGTGCAGGCGCTGCAGTGGGCGGCGGGGTCCGTGGCCGCGGGGCGGTTCCCCGGCGCGAGGGAGGTGATGCAGGCGGCGCAGTACGTGCCTGCCGGGTGCGACGGCATGGTGGGCGGCGGCGTGGCGCTGCCCTCGGAGAACGAAGGCTTCGCCACCATGGCCTTCGTCGCGCACGCCGTTCTCGCCACCCTGTCCAATGGCTACTGA

>SbPMEI50

CTTGCTCGCTGGCCCATAAATATATACTCATCGCCACCGAGCATATATTCTCTATAACAGAATAAACCATGGGGCTAGCCTACTACTACCACCAACGGCTCGTCCTCCTGGCCGTCGTGGCGTTCCTATGTGCTGGCCTCTTCCCGCAGGCATTAGGGAAGGGGCATGGCGGTGCTGTCAACCCGGCGGTTGCCGGCATCTGCTCTCGCACCCCATTCCCTGAGGTTTGCAAGTCCACAGCCGGGAGGCATGCGTCCAAGTACCCGGTCATCGACAACTTGGCCGTGCTCAACATGCAGGTGGAGGCGTTCTCCAAGCGCACTGCGCAGGCGCGGCAGCACGTCGCGAAGTCGGCCCGCACTATTCCACCGGCGCAGAAGCAGGCCCTTACATTCTGCGACACAATGTACATGAACACACAGGACACCATCGGTGCGGCGCAGCGGGCCATCACGTTCAAGGACACGAGCACCGCAAAAATCATGCTGCAGCTCGCCGTTCAGGACTTCGACTCGTGTGACCGTCCGTTCACCCAGGCTGGTATCCCCAACCCCATGGGGAAGTTCGATAAGGAACTAAACCAGATGGCCAACAACTGCATGACGCTTGCAAACATGATATGAACCAGGCATGCGGAGTCAGTTAGGCATGCTTGCACCCCCCAGCGTGAGGTGGCACCGCATGCAGGCAATGCTTGGACGAGAGGACTTGATTTTTCACTTAATAACTCACTGAAATCATGAGATATATTGAGCAACTTTATGTATTTGTTCCAGACCTACATTTATTCAGTAGGTCATGAAAAGAGGTAGAGGGCCTTTCATTGCTTTTCATCTTTTGATTGCTAGAGGTTGCTTTCCTTTGGTTGTTCAGTTTTCTAACATACCACGTCCTTGGCTTGAGACAATTGTAATTATGTAATTAGAAGCTCCCCAAAGTATACTACAAAAAAATATTTTAGGAGATACCTCCGTTTATTTTTAGAGACGGACAAAAAAATTGCAGCCTCTGGGCAACTTACCTACTTGGCACTAAAGAAAGTTCTTAATTCCTTATCTAGCACAAAAAAACATTTGGCTCCTACGGTCCAACA

>SbPMEI51

CCTGTTGCGGCCAACTTTGGTTTTCAGTATTCCTATACACTTCCAGCTAAGACTGCTAGGTATATCTTTCTGTGATTAATGACGCTAAATACCATTTTTGTGTATGATTCAGATTCTTGTGATAGGGCATGAATACACGTTCAGAGCATATATTGGACTAGCTCAGTAGGCTTCAATGGACCGATGATACACATCTTTCTGCTGTCAAGCTGGCAACGCAAGCAGCTATGTTTAGGGGGGAGCCTAAATAAAACAAGGTGAGAGACCATGGCGGGGAACCACGAAAACGAATGGGAGAGACCATGGCGGGGAACCACGAAACCCAAGGAAAAGCCGAAAAGCCATGGCCGAACCTGTGGCCTCCCCCATTCGTCTCTCCTATATAAACCACACGCCGTTTGGAAGCAGAACCATCAGCCTGTGCATTCTCTCGGCCTTCGCTTCTGTTTCTTATTAGGCCGCCCTCTACACTCCTCGAAATAACAATCGAGGGCGGTCAAGAGAAACGAGAGCGGCAGACACCATGGAGAGCTCGAGGATCATCGTCGCCTCCCTCCTCCTCCTCCTCCTGGCCTTCGCAGCCACCGCCGAGGCCCGCGTCGTCCGCGAGCTGATCGGCGAGAACGCATGCCAGCAGACATGCAACCAGGTAGGCACCGCCGCACCACATCCCCTCTGTATTGTGCTGTGCTGCAATGCATGAGAATTTTTGCATAGGTCGATCGATCTGTCTGTGGATGGCACTGGATTTGCTGATCGATGCTCCGCATCGCGCGCATTGCAGGTGCACTTCAAGAAGATGTGCCAGAGCTTGACGAAGCTCCCGAAGGTGACGACGCCGCGGGAGCTCCTGCTGGCGTCGATGCGCGTCGCGGCGGAGAAGGCGAAGGAGGCCAAGAGCCGTGTGGACGAGTACGCGGCGAGGTCCCACGAGGGCCGGCCGATGGAGTCTATCCTCAGTTCCTGCAGCTCCGGGTACGACAACGTGGTGCAGACGCTGGAGGAGACGGAGAAGATCGTCGCCACGCAGGGAACCCAGGTGGACTTGAACACCAAGCTGTCGGACGCCGTCACGAGCGCCGGCGACTGCGACAACGCCTTCCAGGACTTCCCGGAGATGAAGGACCCCTTCTTGGCCATGCAGCGGAACGTCTGGCGCCTCGTGGACAACGTCCTCAACATCGCCGTCGTCGTCAAGCAGTCGGGGGACGCGCACGCCCACTAGCCGCGTGCGCGCTGTAAAAGCAGGGGTGGTGTTGTATCGATCTCCCGCACTTGAATATATATGAACAAGGCTTGGGAAAATTCTGTATTACTAGTAGTGTAACACGGAATTACAGGTTGAGCTTCTATCATGTACTTTTTTGTTCTTGGTTCAAAAAAAAAAACAAAGTCCCCATCTTGCTTTTTCAAAGGCTAACTAATTTCAAATTTAACCAAATCGATATTAAAAAATGCTAGAATTTATGATCTACTGATCTTCCTCCCTCATCCTCCTCCTGTCCGTGCGCATTTTAGACCTGTGAGCATCATGGTGAGTCTTTTATTACGACTTGTTATTGGGCATTTGATTACTCGGTCCTTGGATTGTTCTCACCTTGTTGTTGATCGATTTCTTGCTCGTCTCGGTGTGGTGATCCTTGTAGGAGGATGGTGCGCGATATGTGGAGCGGTCGTCGGAGGGGAAAGTGGACCCTGGACCGGAGGAACTGACAGGTGACCAGGGACCAGCACGGTGTGGCATGCTTGCCGCCTTCGTGCTTGGCGAGAGAGCGGTTGGGGGAGACGAGAGCTCGAGGAGAAACAAAGAGGAAGAGGGAAGAGAGGCGCCACTACCTTTTACCATGCTCCGATCTATGTCAAGCCACGCAAACAAG

>SbPMEI52

ATGGGTGGTGTCACCGTGACGACGCCGCTCTGCTACCTGGCAGTGGCCGCGGCCTCGGCCCTGCTTCTGCTGAGCACCGCCGTGGCGCCGTGCAGCGCGCAGGTGGTGGTGACCATCGAGGAGGCGTGCCGGATGGCGACGAGCGGCGGCGCGGGGAAAGTGAGCTACGACCACTGCGTGGCGTCGCTGGCGTCGGACGCGCGGAGCCGCGACGCCGCGGATCTGCACCACCTGGCCGCGCTGGCCGCGCGGATCGCGGTGGAGCACGCGGCGGCGACGGAGGCCAAGATCGAGGACCTGGGCGAGGTGGAGGAGAGCCCCCACGCGCGCGCGCGCCTGCACCACTGCCTGGACCTCTACAACGCCGCCGCCGACGTCCTGCGCGACGCGCTCGACAACCTCCACGCCCGCGTCTACGGCAAGGCGTCCCAGCAGCTGGCCGCCGCGCTGGGCGCCGCCGAGAGCTGCGAGGACGTCTGGAAGGGCGAGGAGCACGTCCCCGTCGCCGCGCACGACAGGGAGTACGGCCGCATGGCGTTGGTCGCGCTCGGCCTCACCAGCGGCATCGCTTGATCGCGCCTACTACGCTAATGCTGTCGTCTCATCGATTCATCATCGCGTCGATCGATCGGTCCATTACACTTGTCAGCCCGAACGCCATTGACGACGCCATGGCTGTCGTCAAGTCGTCATCATAAAAGCTATATAAAGTCGCATCATCTGTCTCCTTGGAAGTTGGAAATATAATCCTGACATGCATGTATGTAGAAAGGGCATGCGTGCATGTATGTAGGGCGATACATATACACTCCTATCTGTTGTTGTTCGTTCTTTGGGTTTGTAGGACTTTGAACGGTTGCATGGTGGTAAGTATATAAATCACATCTTGAATGAACTACCACACAAGATCTTGCAAGGGCTAATTCCCCCCTCTATTTCTGTTTATCTTATCATTTCTATTGTCATATGATCTTTTTATACTATTACTAGCATGGCTTG

>SbPMEI53

AACTGATCCAAGCTACTAGCTATACAATGGCAGCTCGATCGATGGCCATGGCGTTCCTGACCACCGCGCTCGCCGTGGTGCTCCTCCTCGGTGCCTGCGCGGCATCAGCACCCTGCCTCACCACCGACGCCGCCGCCGCCGCTGTCTGCCAGAACCAACAACGGCACGACGACGACGACGGCGACCTGGTGGCGACGGCGTGCGAGAGGGCCAAGGGCCACGAGGCGCACCACTTCCGCGGGCTGGGTCTGACGGCGCTGACCAAGGACTTCTGCGAGACGACGCTCCGGTCGGACAACCGGAGCGCGGCGGCGAACGACACGCGGGAGCTGGCGCTGGTGGCCATGGACCTGGCCAGCACCGCCGCCGCGAGCGCGAGCACCAAGGCGCGTAGCGCGCTCCGGTCGTCGGGGGGCAGGGGCGGCAAGGACAGGGACACGGAGTTCTCGCTCCGGTACTGCGTGATGGACTACGGCACGGTGGCGGCCGTCCTCCCGGCGTGCCGCGTGATCGTCGAGGAGTACAGCCCCGGCGACTTCCAGGCGCCGTTCGACTACCTGGAGTGCGCCGGCAGGGTGATGGACGCGGCGGGCGACTGCTGGCAGCGCGTGTCGTACGAGGATGGCGAGTTGAAGAGGGCGCTGTGGAAGGACGCCGTCGACGTCGCCAACCGGGCGAACCTCGCCCAGGCCTTGGTCGAGCAGATGGTCGATTTCCCCGACGATCATCACTGAATAATCATCATGTTAATGTACAATAAGAGAATTACCAATTTTTCTCACAACAGCTTGAGTTTTGGTATGAAATTGCTTTGGATCAGTGTATGCAAATCAAATCAAATAATACGAATATAGTGGTGATCAGTCAGAGCTGCTACTTTGTTC

>SbPMEI54

ATGGCTCGCCTGCTCCTCCTCCTCGCCGCCGCCGCCGCAGCGGCGTTCCTGGCCGTGGAGGCCGCGTCGCCGGTGGCCAGCGACTTCATCCGCAAGTCTTGCCGCGCGACGCAGTACCCGTCGGTGTGCGAGCAGAGCCTGGCGTCGTACGGGGGCACCCCGCCGCCGCGGAGCCCGCGGGAGCTGGCGCGCGCCGCGCTGTCCGTGAGCGCGGACCGCGCGCGCGCCGCGTCCGCGTACGTGGGCCGCCTGTGCGGCGCCGGCACCGGCGCGAAGAAGGGGTCCGGGTCGCGGCCGGCGGCGGCGGGCCCCGTGCGCGACTGCCTGGAGAACCTGGCGGACAGCGTGGGCCACCTCCGCGACGCGGCGCAGGAGATGGGCGGCGCCGGGATGAGCCGCTCCGGGACGCCCGCGTTCAAGTGGCACCTCAGCAACGTCCAGACCTGGTGCAGCGCCGCGCTCACCGACGAGAACACCTGCCTCGACGGCCTCTCCTCCCGCGGCGTCGACGCCGGCACGCGCGCCGCCATCCGCGGCAAGGTCGTCGAGGTCGCGCAGGTCACCAGCAACGCGCTCGCGCTCGTCAACAAGGTCGGGCCTGGGTACTAG

>SbPMEI55

CATTCAAGAGAAACAACCAAGAGCCCGCACTCTCACTGTGACTCCGTGAGCACACTCGCCGCACCAGCTCAGCTCAGTAATCATGGCTCGCCCGGGTAGTAGTGCCGCTGCTCCCCTCCTCCTGCTGCTCGCCGCCGCGGCGGCGTCGATCCTGGCAGCGGCAGCCGCGTCGCCGGCGCCGAGCGACTTCGTCCGCAAGTCGTGCCGCGCGACGCAGTACCCGTCGGTGTGCGAGCAGAGCCTGGCGTCGTACGGGGGCTCCCCGGCGCCGCGGAGCCCGCGGGAGCTGGCGCGCGCCGCGCTGTCGGTGAGCGCGGACCGCGCGCGCGCCGCGTCGGCCTACGTGGGCCGCCTGTGCGGCGGCTCCAGCAGCTCCGCCGGCCACAAGAAGGGCGCTGCTGCGAGGAAGGGCGGCGCGCCCGGGTCGGCGGCGGGACCCGTGCGCGACTGCCTGGAGAACCTGGCGGACAGCGTGGGACACCTCCGCGACGCGGCGCAGGAGATGGGCGGCGCCGGGATGTCCCGCTCCGGGACGCCCGCGTTCAAGTGGCACCTCAGCAACGTCCAGACCTGGTGCAGCGCCGCGCTCACCGACGAGAACACCTGCCTCGACGGCCTCTCCTCCCGCGGCGTCGACGCCGGCACGCGCGCCGCCATCCGCGGCAAGGTCGTCGACGTCGCGCAGGTCACCAGCAACGCCCTCGCACTCGTCAACAAGGTCGGGCCAGGGTACTAGCCCAGGCCCAGGCCCAGCCCAAATAGCCCTCGCTACTTAGTCCATACACGTATTATACTTCGGTGCGTCGGTTAAGTACGTAGACCAGTTTTCCACGTGTTGCTTCTGCTTATGATTCCTCGTGTTTGTAAGATGTCGTTAAGGGCGACAATGATAAAGCTTGTAATATTATGGACTTATGACAACAAGTACAGTAAGCATGTACTACAAAATAAGTCTCATCGTTACGTTCATATGGTCTTCGAGAAACGAAAATTTTCTTTTACTACCGATCTTTCTCCAATGTGGGGGTGTCACTCATTTTTAACAGATGGAGTAAATATTATTGTTC
